# Tyrants, democrats and the first silver ‘owl’ coins of Athens

**DOI:** 10.1007/s12520-025-02229-z

**Published:** 2025-05-09

**Authors:** Gillan Davis, Janne Blichert-Toft, Liesel Gentelli, Francis Albarède

**Affiliations:** 1 Faculty of Education and Arts, Australian Catholic University, North Sydney, Australia; 2 Laboratoire de Géologie de Lyon, Ecole Normale Supérieure de Lyon, CNRS, and Université de Lyon, Lyon, France; 3 Australian Catholic University, Tenison Woods House, 8 Napier Street, North Sydney, NSW 2060 Australia

**Keywords:** Lead Isotope Analysis, XRF, Athenian coins, Silver, Owls, *Wappenmünzen*

## Abstract

Late in the sixth century BCE, the ancient Athenians overthrew the previous tyrant rulers and adopted a new democratic form of government militarily establishing themselves as a regional power. They constructed a fleet which played a crucial role in defeating the Persian invasion of 480/79 BCE and led to their creation of a naval-based empire. A key funding source for the ships was silver from the domestic mines at Lavrion situated in the south-east corner of Attica (Hdt. 7.144; *Ath. Pol*. 22.7). The current consensus based on earlier metal analysis is that the Athenians must have found and exploited the rich, so-called ‘third contact’ silver mines in 520–515 BCE, a discovery that led to the introduction of the larger tetradrachm monetary unit (Kraay, 1956) and the iconic ‘owl’ coin ‘type’ (Picard, 2001) as a branded medium for exporting silver. Here, we present new lead isotopic and elemental abundance data for 52 archaic Athenian owls (pre-479 BCE) combined with previously published legacy data for 12 other owls. The results show that only a small proportion of the coins were wholly struck from Lavrion ore or other discrete sources while most of the coins were struck from Lavrion ore mixed with ore from a single or homogeneous, geologically older source. Crucially, this was not the multitude of external sources used for the preceding *Wappenmünzen* series. We deduce that the discovery of the third contact only occurred shortly before 483/2 BCE, precisely as claimed in the literary sources, at which point Lavrion silver came to represent the bulk of the supply. This forces a reappraisal of the current paradigm since it seems that there was no abrupt and complete change of ore source to Lavrion, and therefore this cannot be connected with the introduction of the owl coin type. It also impacts our understanding of the very low gold content of Athenian owls compared with the earlier *Wappenmünzen* series, which should be attributed to the replacement of ore sources.

**Supplementary Information:**

The online version contains supplementary material available at 10.1007/s12520-025-02229-z.

## Introduction

In 2001, Olivier Picard influentially wrote that the ‘discovery’ and exploitation of the immensely rich, so-called ‘third contact’ veins in the silver mines of Athens situated at Maroneia-Camariza in the Lavrion district of south-east Attica must have taken place around 520–515 BCE (Picard 2001). This was despite literary evidence about a debate in the Athenian assembly which seems to clearly show that the discovery took place in, or at least closely preceding 483/2 BCE (the archonship of Nikodemos), since the debate concerns whether the state’s share of the funds should be distributed to the population or used to build a fleet (*Ath. Pol*. 22.7; Hdt. 7.144). Picard based his conclusion on three propositions: first, that it would have taken many years of mining to reach the ores post-discovery; second, that Lavrion ores are low-grade and hence amassing the quantity of 100 talents of silver available to be distributed could only have happened over an extended period of time; and third, based mainly on elemental analysis, in particular gold contents, that the change of coin type from the earlier so-called *Wappenmünzen* (literally ‘heraldic coins’) to the owl was linked with a complete change to the Lavrion ore source. As Picard pointed out, this was not entirely a new idea. Ardaillon's (1897, 136) work on the Lavrion mines propounded the theory of the first and third contacts, and Conophagos (1980, 94), who oversaw re-mining of Lavrion until 1978 and put in writing what he had learnt about ancient Greek mining at Lavrion, had both proposed 500–495 BCE as the starting date of renewed intensive mining at Lavrion. Picard’s insistence on an even earlier date stemmed from his correlation of exploitation of the mines with a monetary reform of the tyrant Hippias (Ps-Aristotle, *Economics* II, 2, 4b). At the heart of the paradigm was the earlier understanding that exploitation of Lavrion in the late sixth century BCE was strongly linked with the production of the new series of large denomination, overtly Athenian, owl tetradrachms for export of silver under the tyrants (Kroll and Waggoner 1984; van Alfen 2012) based on the lead isotope analyses of Gale et al. (1980; supported by Nicolet-Pierre et al. 1985). This understanding has formed the basis for discussions about the rule of the Athenian tyrants and the new institution of democracy at Athens. Critically, the effects that the silver would have had on the economy from the late sixth century BCE has major implications for understanding Athenian finances in the vital transitional period to empire from the last quarter of the sixth century to the first quarter of the fifth century BCE.

In this work, we present new lead (Pb) isotope data for 52 late Archaic period owls and three later Classical owls, the latter included for diagnostic reference purposes only. The new Pb isotope data were combined with legacy data for 12 additional owls, all of which we compare with our lead isotope database of c. 7000 silver ore samples from ancient mining districts spanning locations across Europe and around the Mediterranean from Spain to Iran (10.5281/zenodo.14879274, also accessible as document 15119912 on the repository http://zenodo.org/). We also present new major and trace element data for the coins we analysed for Pb isotopes. We build on our recent findings about the ore sources of the *Wappenmünzen* coins (Davis et al. 2025) and on the geology of the Lavrion silver sources (Vaxevanopoulos et al. 2022; Ross et al. 2021). The results allow us to shed new light on the development of Athenian coinage and its ore sources and hence on finances in the late Archaic period.

## Materials and methods

We analysed 52 Archaic owl coins for their Pb isotope and major and trace element abundances together with three later owls for comparative purposes (Table 1). The Archaic owls are all from the collection at the Numismatic Museum of Athens (NMA), Greece. The later owls of low numismatic value were purchased from a certified dealer and are held by the lead author.

**Table 1:**
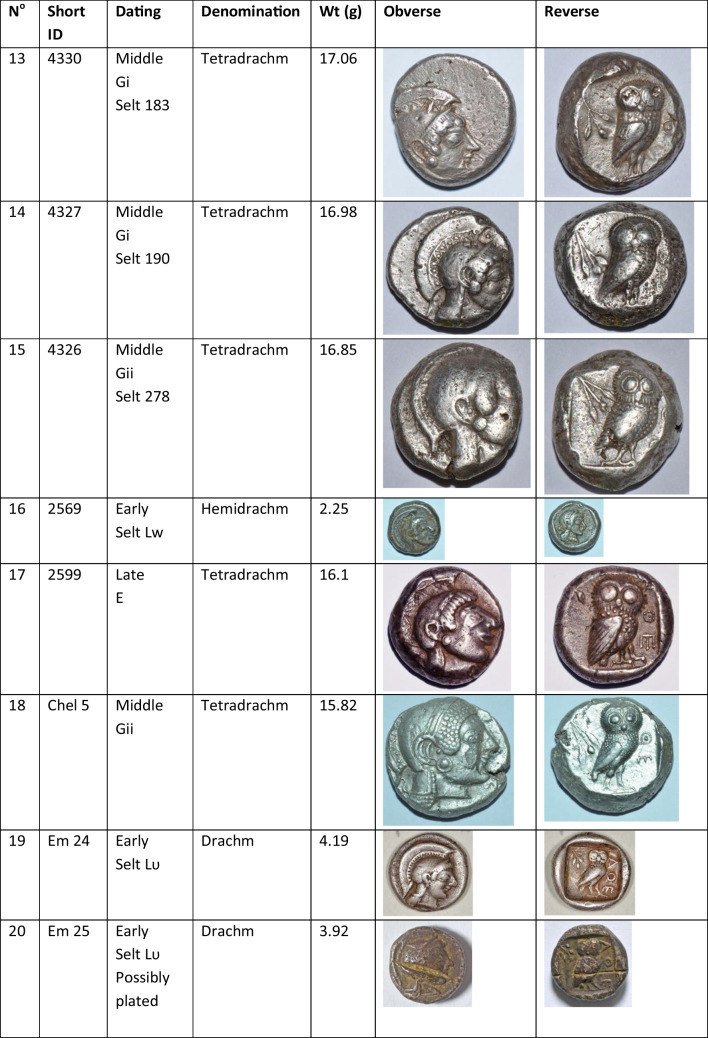

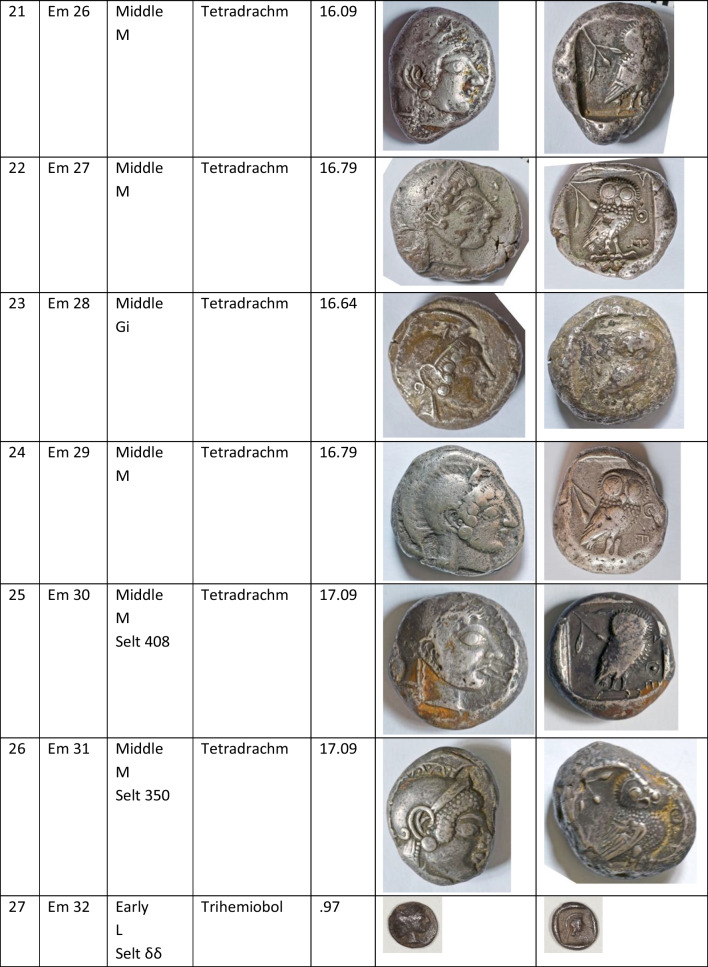

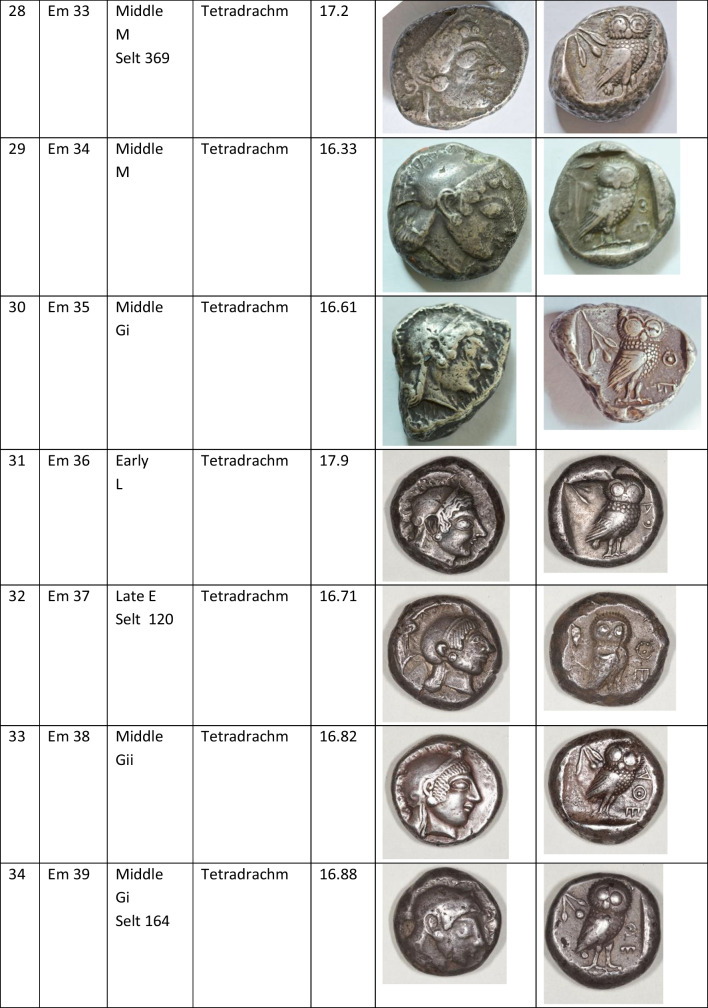

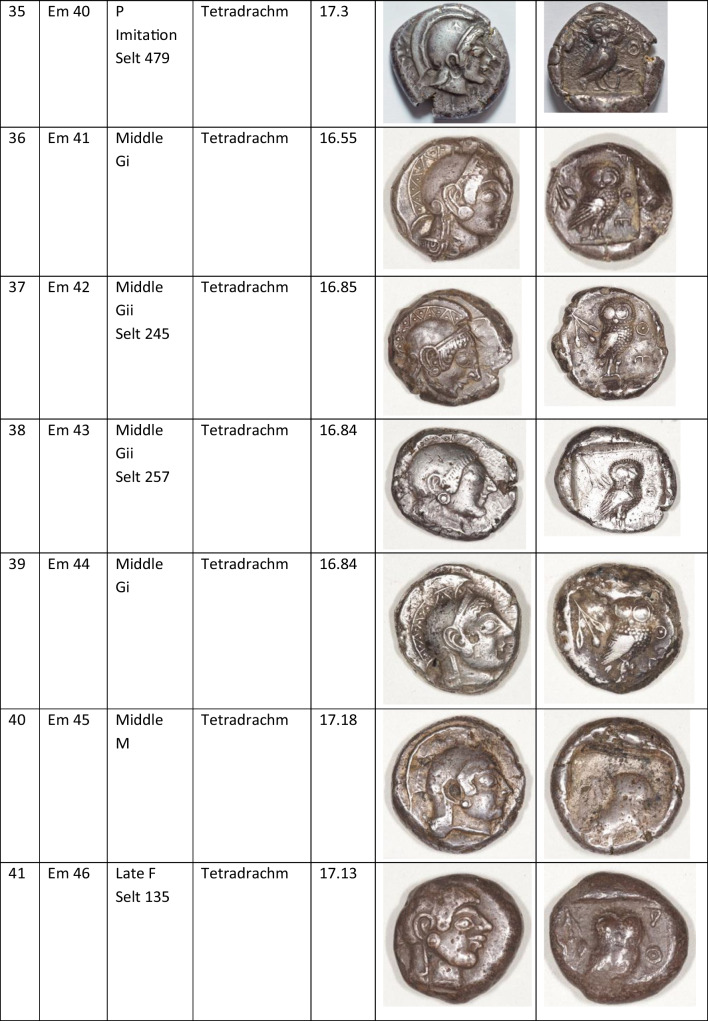

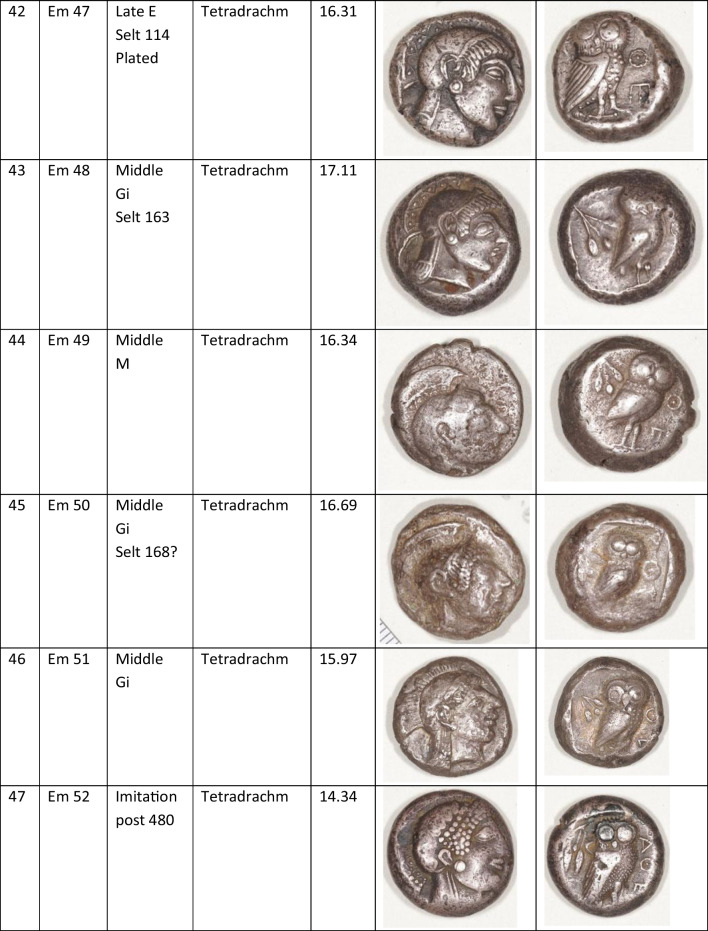

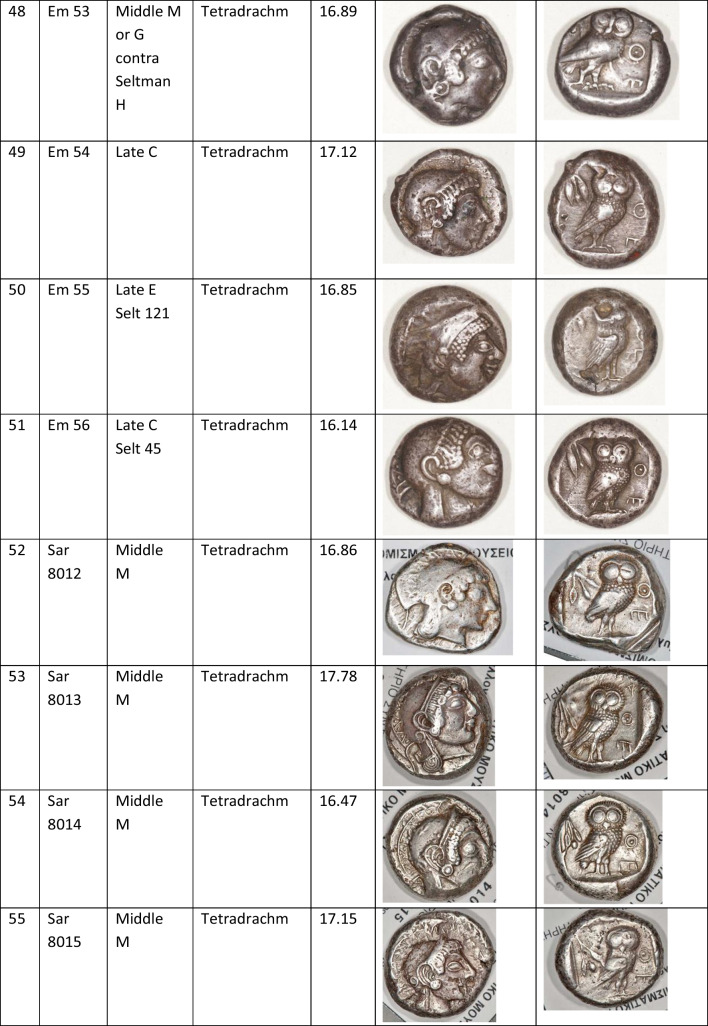

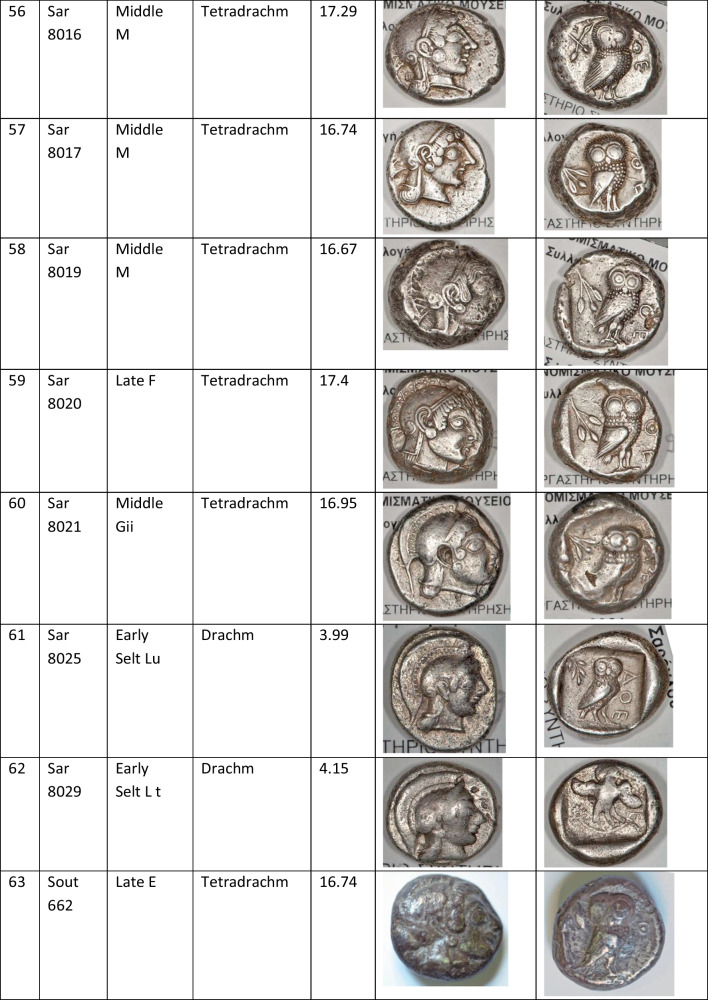

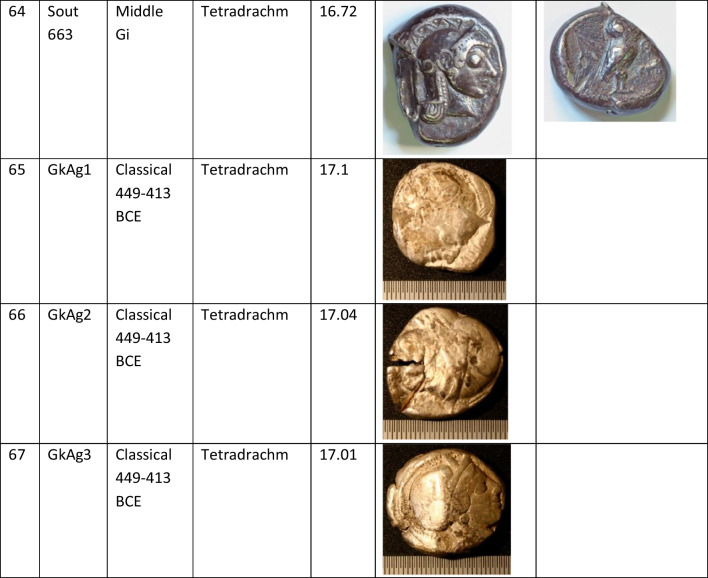
Table showing the coins (not to scale) sampled at the NMA with permission from the museum’s director

Photos by the authors. Coins 1–12 are legacy data from the OXALID database comprising unidentified coins supposedly from the Asyut Hoard previously analysed by Gale et al. (1980) and therefore cannot be shown. ‘Selt’ is for Seltman (1924) with the letters corresponding to the types he described with the order modified by Kraay (1956): Early = H & L; Middle = M, Gii & Gi; Late = C, F & E. Although very dated, this is a broadly acceptable chronological scheme but one requiring a new study especially of die links to establish the extent to which dies were used concurrently rather than sequentially. The archaic owl series commences in the late sixth century (the date is contested, see discussion below) and ends at 479 BCE.

The 52 coins from the NMA were sampled for Pb isotopic analysis at the NMA, while the Davis coins were sampled at the Laboratoire de Géologie de Lyon at the Ecole Normale Supérieure de Lyon (ENS Lyon). After sampling at the NMA, the samples were transported to ENS Lyon, where Pb separation and isotopic analysis were carried out for both the NMA and Davis samples. The coins were sampled using a virtually non-destructive technique in which only a few micrograms of material were removed from each coin. Details about the sampling and Pb separation methods as well as Pb isotope analytical precision and accuracy are given in Milot et al. (2021). Elemental analyses were conducted at the NMA using a PANalytical Epsilon3 benchtop XRF with 50 kV Rh anode tube. Details are in Gore and Davis (2016). The Pb isotope data are provided in the Supplementary Material. The major and trace element data are listed in Table 2.

**Table 2:**
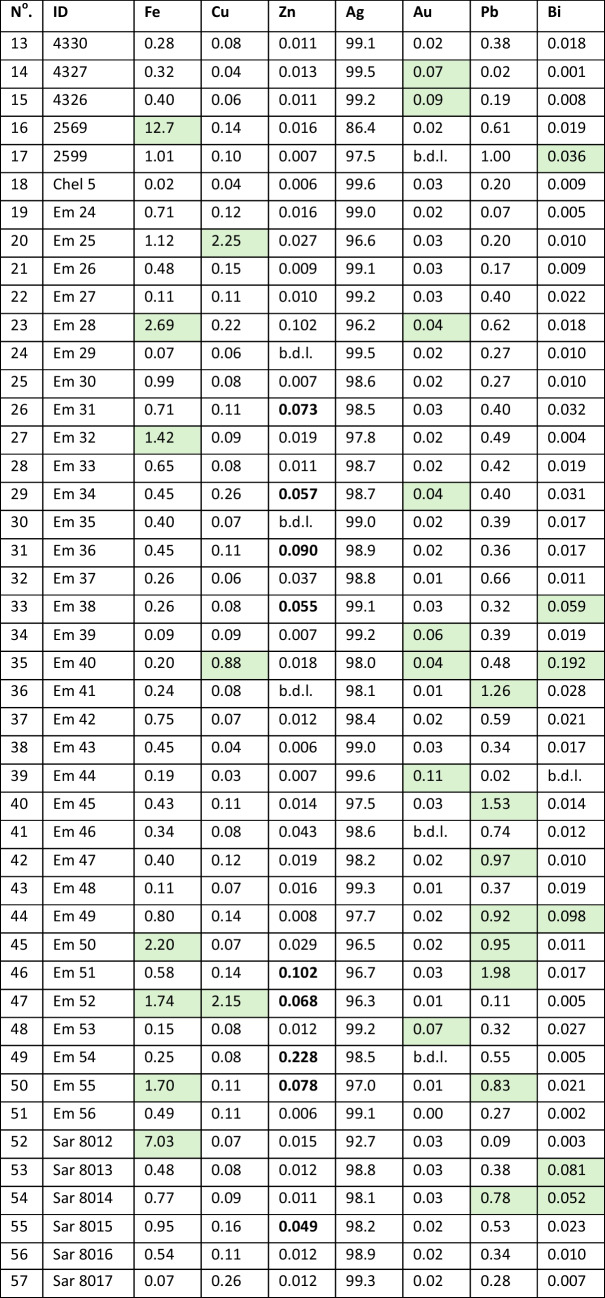

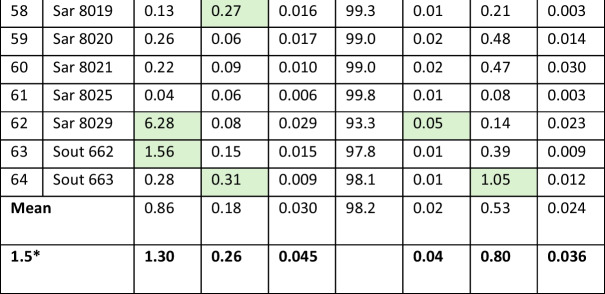
Elemental concentrations of the coins analysed in this study. Results are in weight percent. The cells shaded in pale green show coins that equal or exceed 1.5* the arithmetic mean concentration of an element. Zinc has values below the detection limit (b.d.l), but coins exceeding the arithmetic mean concentration by 1.5* are in bold

We reevaluated the Pb isotope data for the 12 coins published by Gale et al. (1980; re-examined in Stos-Gale and Davis 2020) for which analytical data are provided (as not all of the coins in Gale et al. 1980 have data). They are part of the Asyut hoard (*IGCH* 1644), but the information provided is insufficient to unambiguously identify them as such, even though five are coin fragments in the British Museum (see Supplementary Material).

## Results

**Fig. 1:**
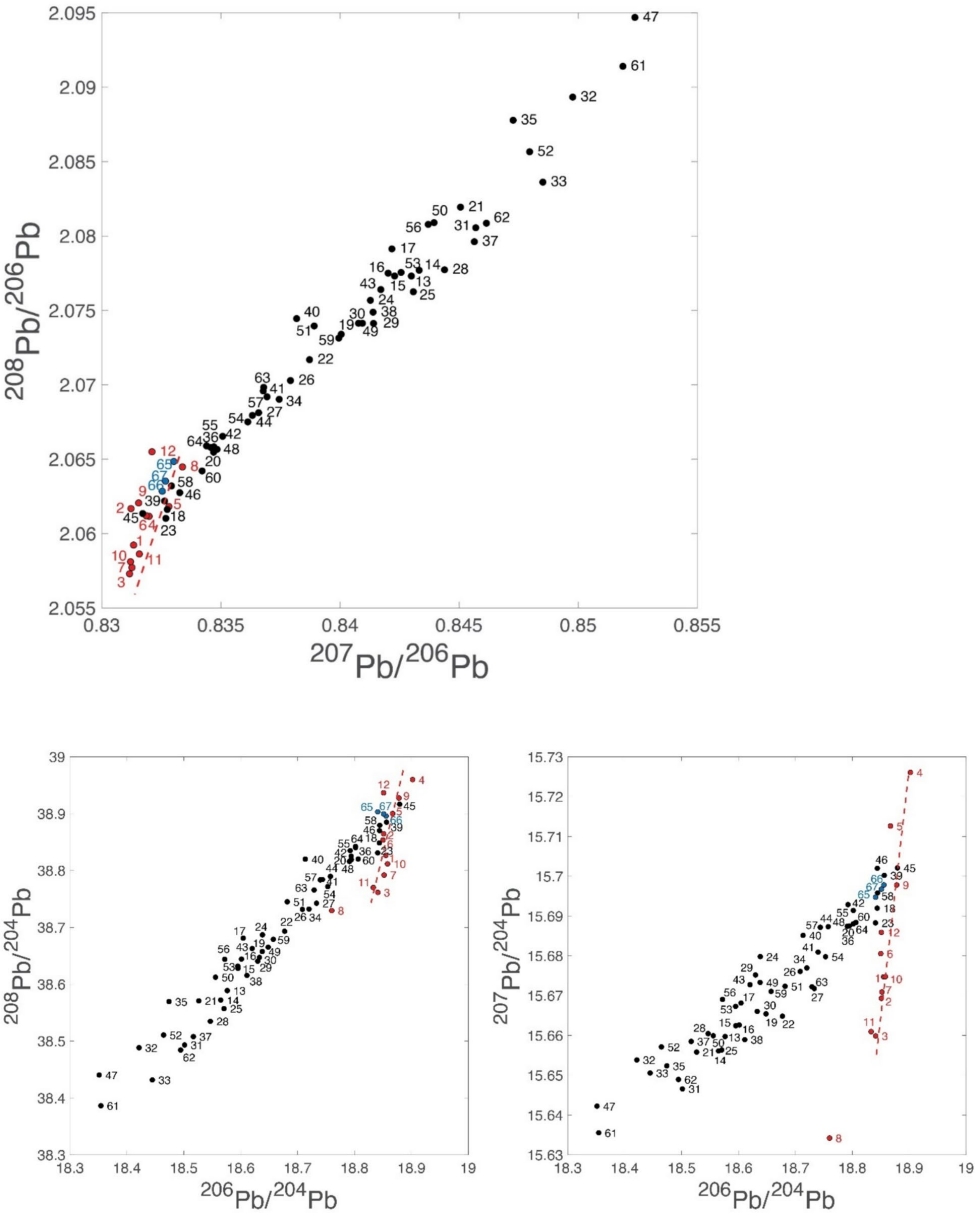

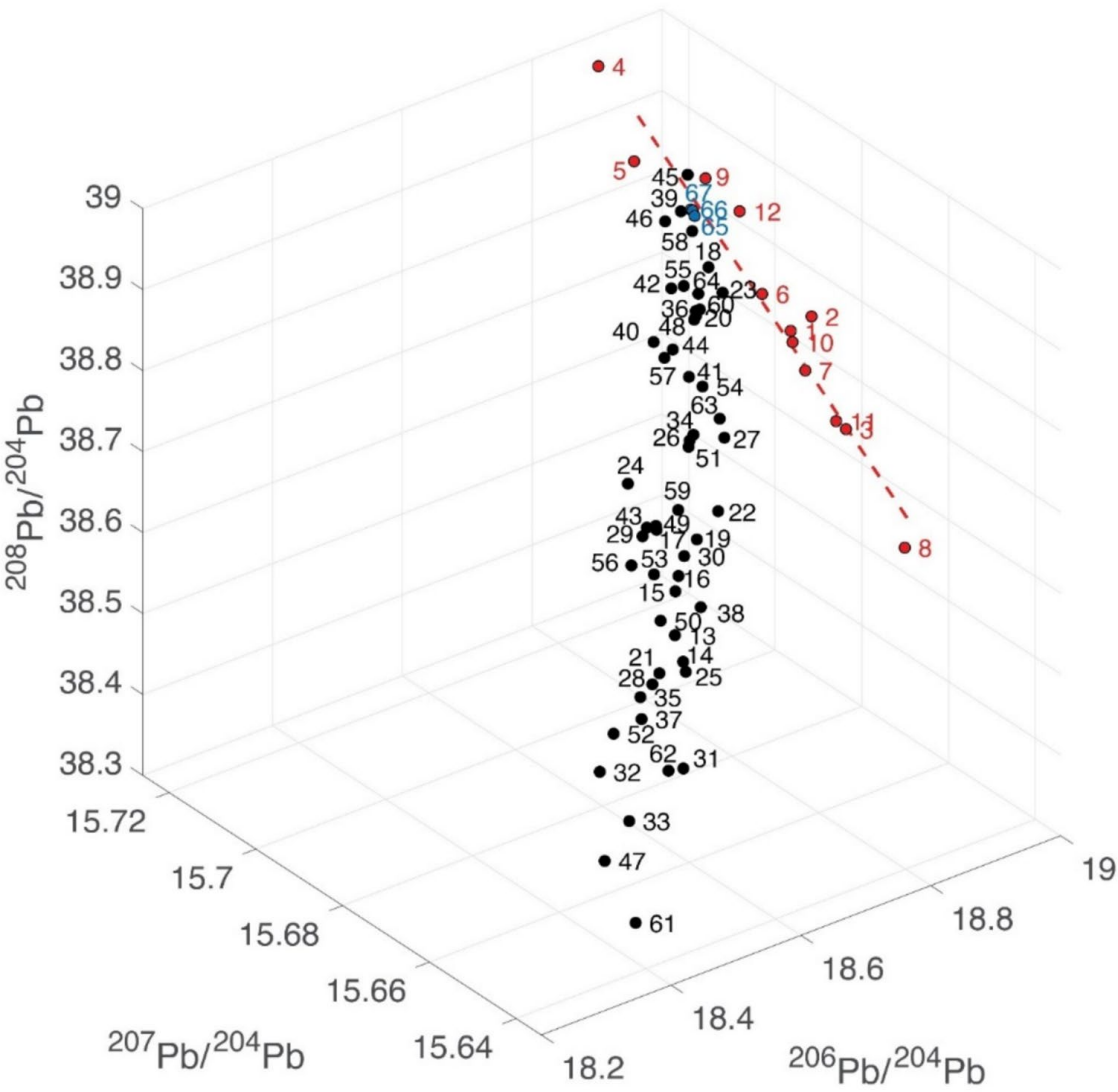
2D and 3D plots of the new Pb isotope owl data from this study (black dots), the three Classical owls (this study: blue dots) used as reference and the Asyut hoard (legacy data from Gale et al., 1980: red dots). The bottom left-hand corner of the ^208^Pb/^206^Pb–^207^Pb/^206^Pb plot represents the geologically young Lavrion ore field. The upper right-hand corner is the geologically much older Sardinia mix. In between these two end-members stretches the mixing array. The red dashed line shows the theoretical mass-dependent fractionation line going through the red points representing the Asyut hoard coins demonstrating that their spread is due to analytical issues

Figure 1 provides 2D and 3D plots of the Pb isotope data with the numbers corresponding to the coin numbers in Table 3. The data points form a remarkably binary mixing line between Lavrion values, which are well documented, and those of a second ‘end-member’. This end-member may represent either a single unknown source or result from a nearly homogeneous combination (mixing) of different sources, something which we have previously referred to as the ‘Sardinia mix’ (Albarede et al. 2024a, b). The slope of the red dashed line going through the Asyut coin hoard data set (the 12 legacy data) in all Pb isotope spaces is identical to the theoretical slope expected from mass-dependent Pb isotope fractionation. Since the 52 new Pb isotope data do not show this effect, we conclude that the spread of the Asyut data points is of analytical origin. All the Asyut coins seem to have been minted shortly before 480/79 BCE and are from Lavrion ores.

**Table 3:**
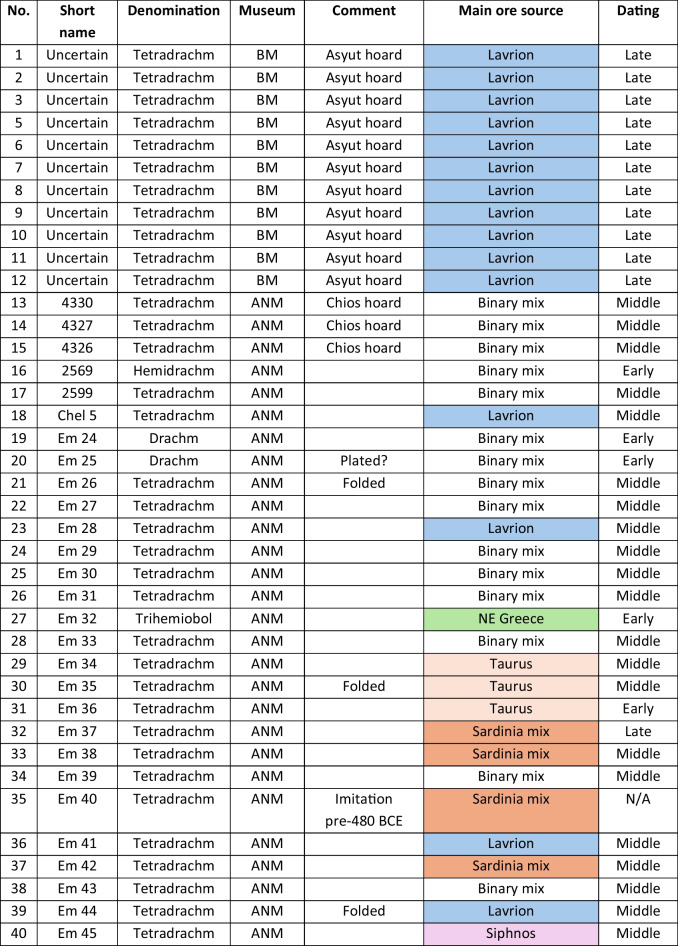

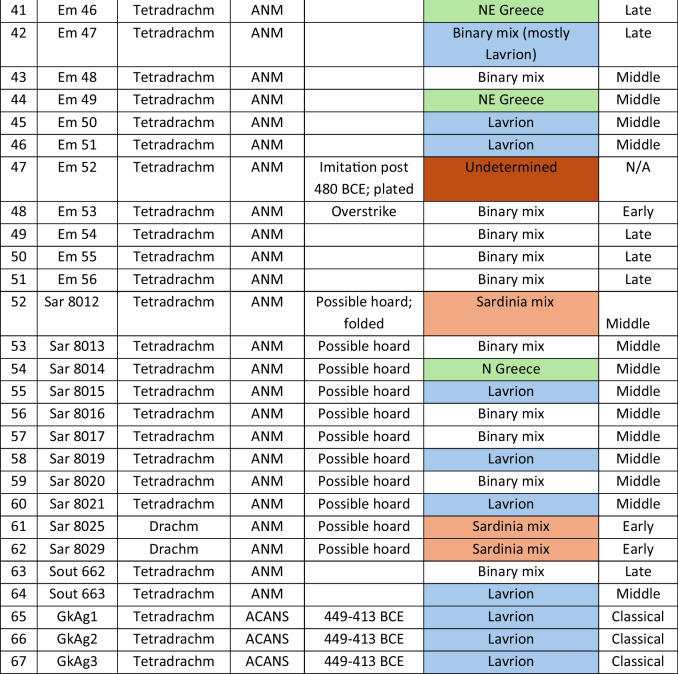
Summary of the results and interpretations of the hit map for each coin correlated with the relative dating from early to Classical. ‘Mix’ under ‘Main ore source’ means a mixture between ores from Lavrion and the ‘Sardinia mix’

**Fig. 2:**
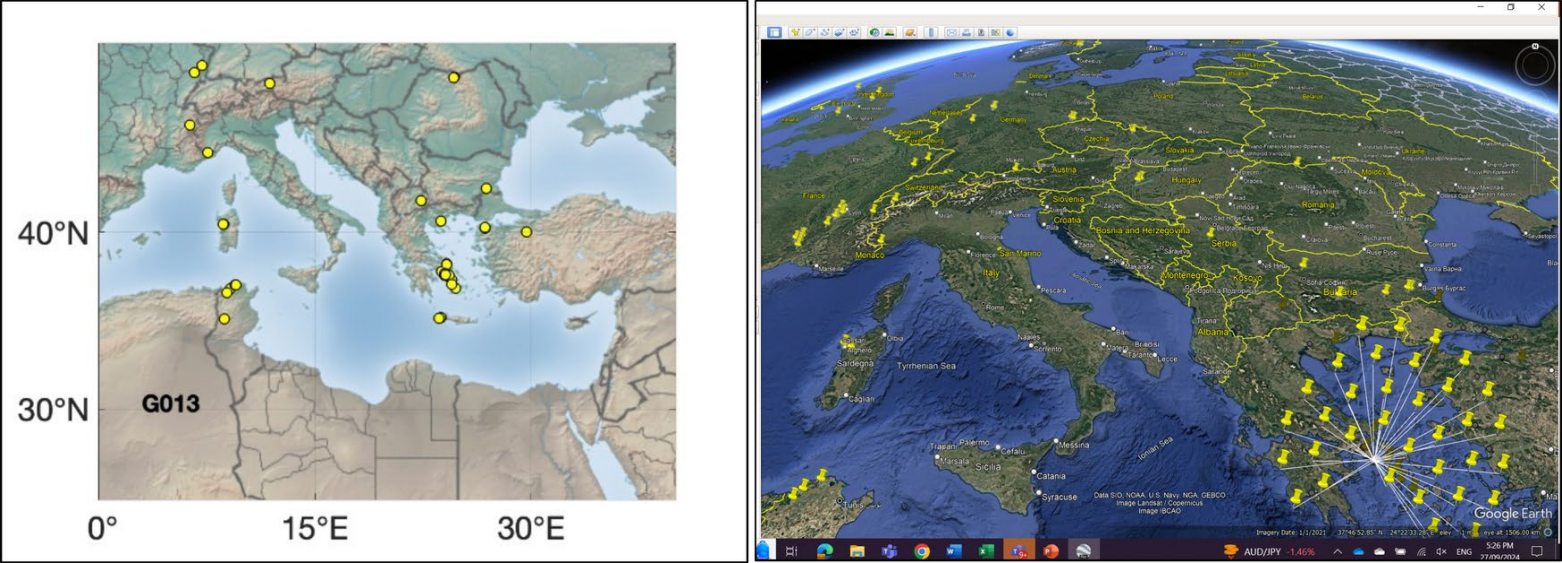
Left panel: Hit map of coin #65 showing possible sources with a cluster around Lavrion. Right panel: The same coin geolocated using Google Earth maps unambiguously points to Lavrion as the silver source

Figure 2 is an example of an indicative hit map with its corresponding Google map. The maps indicate that the ore source of coin #65 (GrAg1) is Lavrion. Individual maps for the other coins analysed in this study are provided in the Supplementary Material.

As already mentioned above, Table 2 presents the elemental concentrations of the coins analysed in this study.

As also mentioned above, Table 3 summarises the ore source attributions together with the likely date of manufacture of the coins. It shows that there is very little correlation between the two except for the coins from the Asyut hoard and the three Classical coins which are entirely from Lavrion ores. Some of the newly analysed coins are from Lavrion ores and some are solely from the Sardinia mix. Three are primarily from Northern Greece, three are from Taurus and one is from Siphnos. However, the majority of the coins is a mix of the two dominant end-members (see discussion). One coin (#35) is a pre-480 BCE ancient imitation (not to be mistaken for a forgery), and another (#47) is a post-480 BCE ancient imitation.

Table 4 compares the arithmetic mean of four diagnostic elements – Cu, Ag, Au, Pb – between the earlier *Wappenmünzen* series analysed by Davis et al. (2025) and the owls analysed here. The results show that the former have almost six times more Cu and more than 22 times more Au than the owls, but their purity of Ag is lower, and they have minimal Pb contents.

**Table 4:** Comparison of the arithmetic mean of four key diagnostic elements between all the *Wappenmünzen* analysed by Davis et al. (2024) and the owls analysed here

Element	Cu (%)	Ag (%)	Au (%)	Pb (%)	Pb/Cu	Au/Cu
*Wappenmünzen*	1.03	97.5	0.54	0.11	0.11	0.52
Owls	0.176	98.2	0.024	0.531	3.02	0.14

## Discussion

The results of this study are not consistent with the conclusions of previous studies (Gale et al., 1980; Nicolet-Pierre et al. 1985) which suggested that most Archaic owls derive from pure Lavrion ore. In 3D Pb isotope space, the new data reveal a conspicuous mixing trend which is projected on both the ^207^Pb/^204^Pb vs ^206^Pb/^204^Pb and the ^208^Pb/^204^Pb vs ^206^Pb/^204^Pb binaries as linear arrays. At one end is a young, in geological terms, Lavrion end-member (^206^Pb/^204^Pb = 18.85, ^207^Pb/^204^Pb = 15.70, ^208^Pb/^204^Pb = 38.85). At the other end is a second, geologically much older, end-member (^206^Pb/^204^Pb = 18.35, ^207^Pb/^204^Pb = 15.64, ^208^Pb/^204^Pb = 38.4) of exogeneous silver which we have previously referred to as the ‘Sardinia Mix’ (Albarede et al. 2024a, b). This second end-member may not be a pure source. From the minor apparent scatter, it rather seems to be the result of a nearly homogenized combination of different sources mostly from Sardinia, Spain and the Balkans.

A few coins are from pure Lavrion ore. This isotopically unmistakeable source is recorded in the Pb isotope composition of the three Classical period owls included in this study as a reference for Lavrion ores (coins #65, #66 & #67), one of which is shown in Fig. 2. The 12 legacy coins also are from Lavrion (hit maps provided in the Supplementary Material). These plot at the upper end of the ^207^Pb/^204^Pb*–*^206^Pb/^204^Pb and ^208^Pb/^204^Pb*–*^206^Pb/^204^Pb arrays (Fig. 1). They come from the Asyut hoard which is dated by Price and Waggoner (1975, Asyut Group IV) in their publication of the hoard to 475 BCE. However, they are overwhelmingly Seltman groups G and M dated down to c.482 BCE meaning that the coins will have been in circulation for some years or, more likely, that there were many dies concurrently in use at the end of the sequence when minting was massively ramped up. This is the time when the major third contact ores were the predominant ore source and large quantities of coins found their way to Egypt and the Levant (Sheedy and Gore 2011). These legacy data are clearly mass fractionated. The fractionation array intersects the mixing line at precisely the Lavrion value identified by our data on the three Classical owls.

The bullion used for some coins have specific sources including the Sardinia mix, Northern Greece, Siphnos and Taurus; two are ancient imitations, one of which is a post 480 BCE plated coin with a clearly different source plotting beyond the Sardinia mix. Most coins, however, fall in an intermediate position along the mixing line with a greater or lesser proportion of Lavrion and Sardinia mix ores. Crucially, none of these intermediate samples can be undeniably assigned a single source. If the mix included more than two components, the data would scatter, not define the remarkably linear mixing array we find here.

We have literary evidence regarding one major event that resulted in a great deal of foreign silver coming to Athens from a fresh source. Herodotus (5.77) recorded that the Athenians received over 23 talents, equal to 140,000 drachmas of silver in reparations from the Boeotians in 507/6 BCE and another probably similar sum from the Chalcidians to ransom prisoners from a defeated invasion. This would have put sufficient non-Lavrion silver to strike 70,000 tetradrachms into the hands of the authorities if they needed to mint it. We may also assume that the Athenians continued to access silver from wherever they could (Davis et al. 2025; Stos-Gale and Davis 2020) in addition to a regular influx of foreign currency and bullion through trade and harbour dues (Davis 2014a, 273–4). Unfortunately, we do not have sufficient analyses on the silver used by other archaic Greek states which would enable a comparison of sources.

Picard (2001) contended, based largely on Gale et al. (1980), that elemental metal analysis data indicated that Archaic owls have a homogeneous composition consistent with Lavrion ores marked by low gold content, and thus clearly distinct from the preceding *Wappenmünzen*. Our analyses (Table 2) confirm that the gold content is indeed uniformly low, with 49/52 (94%) being < 0.05% Au (the figure we set in Davis et al. 2020). However, the low percentage of gold cannot be correlated with the Pb isotope data and needs to be examined separately to understand the phenomenon. Part of the answer is the extraordinary purity of silver of the coins at a mean of 98.2%, significantly higher than the *Wappenmünzen* with a mean of 97.5% (Table 4). Twenty one owl coins (44%) are over 99% pure silver which is remarkable considering that Lavrion ores are relatively low-grade (Conophagos 1980, 85; but note that Ross et al. 2021 show how grades varied and especially that third contact ores were three times higher on average than first contact ores; moreover, it is worth noting that the high-grade ores were mined out). The purity is indicative of extreme levels of cupellation to the point where a significant proportion of silver would have been lost in the process (Rihll and Tucker 2002, 278–9). But silver purity cannot account entirely for the very low percentage of gold in the owls since gold remains at its initial concentration through the cupellation and minting process. This begs the question why, if external silver was used, which in the *Wappenmünzen* results in a relatively high gold content, do the owls not have more gold?

We wondered whether the answer might lie in extracting gold from silver, but this cannot be correct. It is possible to extract gold from silver by cementation, but only where the amount of silver extracted during gold purification is smaller than the total amount of gold processed, and therefore very small and particularly expensive to produce. To further test this proposition, Albarède et al. (2023) measured platinum-group elements in Greek silver coinage and concluded that the lack of Ir and Rh correlation with Au is not consistent with silver produced by cementation.

Table 4 shows stark differences in composition between the *Wappenmünzen* and owls. In particular, the former have almost no Pb but six times more Cu. In Davis et al. (2020) we demonstrated that Cu was added to the metal mix after cupellation, presumably to make the coins harder and more durable. We suspected that some gold may have been added to the *Wappenmünzen* through the addition of Cu, and that this was no longer the case for the owls. However, given that Pb solubility in Cu is less than 0.65 wt‰ (Teppo et al. 1991), addition of 0.176% Cu (Table 4) to the owls would only add 11 ppm Pb to the 5310 ppm Pb (2‰) already present in the *Wappenmünzen*. Lead isotopes, therefore, are insensitive to the addition of Cu to Ag. Likewise, Au may be present in porphyry copper deposits —the main source of copper— but with the Au/Cu ratios of these ores being less than 1 10^–4^ (Kesler et al. 2002), Cu addition cannot account for the abundance of Au contents in *Wappenmünzen*. It is therefore most likely that the high Au contents of the *Wappenmünzen* were inherited from Au-rich silver ores, which were no longer being used for the owls.

The metal analysis data reveal other interesting information. Two coins have over 2% Cu. One (#47) is an imitation and a plated coin, proven by our analysis of the reverse (not shown here) which has 4.7% Cu. This is confirmed isotopically as it plots beyond the Sardinia mix end-member. It is likely to be from the east, but post-480 BCE, as indicated by the crescent moon on the reverse. The other coin (#20) is an early variety of owl drachm with Lavrion mix ore which may be plated, confirming Seltman's view (1924, p.216, number 9), though not that it was a forgery. Coin 35 is also eastern, possibly from Egypt, but pre-480 BCE. It is die-linked with Asyut 422 (Sheedy and Gore 2011) and plots in the Sardinia mix with elevated levels of Au and Bi. Eleven coins (26%) have < 98% Ag, two of which are < 90%. The main reason for these relatively low Ag contents is the high percentages of Fe, mostly on the surface, and this is probably from circumstances of deposition (Gore and Davis 2016). Many of the coins have elevated Zn. Coins with a high percentage of Pb are associated with Lavrion and, in one case, Siphnos (#40), the latter’s elemental composition being consistent with expectations (Sheedy et al. 2020). Coins #49 and #51 have the same reverse die. Isotopically they plot close together along the mixing line. Coin #49 has significant impurities of Zn. However, though coins #34 and #43 share the same obverse die, they plot well apart on the mixing line, the former being close to Lavrion, the other further away. Chemically, they are almost identical.

The numismatic evidence adds some nuance to the discussion. The analysed coins include three coins from a Chios hoard (#13, #14 and #15). They plot almost identically, and their compositions are similarly uniform with relatively high percentages of Au. These coins have impeccable Athenian credentials. They must have been minted from a batch of silver that naturally contained elevated Au, but from different dies. It seems possible that the 11 Saroglou coins may also come from a previously unrecognised hoard. Coins #61 and #62 are from one of the earliest groups in the series and have Sardinia mix ore provenance. Coin #52 also is from the Sardinia mix. The remaining coins are Seltman group M or thereabouts and plot in or near Lavrion.

Although improved geopolitical circumstances under the tyranny and then the democracy enabled mining to resume after a probable hiatus through much of the sixth century when the polis was politically unstable (Davis 2014a), the yield from the discontinuous low-grade first contact ores was meager. This offers a compelling explanation for why most of the Archaic owls contain only a small Lavrion ore component. All agree that mining of the first contact silver led to the discovery of the much richer third contact silver which spurred investment. However, there is a fundamental misunderstanding in the scholarship quoted earlier about the nature of investment. Investors (excluding major corporations nowadays) cannot afford to wait decades for a return. Mining in ancient Athens was carried out by a complementary mix of small operators (Acton 2014). And although the third contact ores were mostly located at deeper levels than the first contact ores (Ross et al. 2021), there is no need to posit that it would have taken decades to access and exploit them. The find literally was a bonanza for the land owners, entrepreneurial miners and the state (Davis 2014a). All were invested in the success of mining which drew the attention of ancient commentators to 483/2 BCE when funds were amassed for state distribution.

The tyrant Hippias brought in the use of the tetradrachm, double the weight of the previously used didrachm, for the gorgon type, but we have previously shown that these coins were not struck from Lavrion ores (Davis et al. 2025; contra Kraay 1956). Thus, exporting silver from Lavrion could not have been the driver behind the new denomination. Deprived of this rationale, there are reasons to suggest that the change to the owl type was instigated by the new democracy. First, owls are not found in hoards until the fifth century and then initially only from the relatively rare group H (Kroll and Waggoner 1984). Second, we might reasonably assume that the tyrant’s existing trade networks were disrupted by the regime change and no longer accessible to the Athenian democrats, especially the sources from Asia Minor found in the *Wappenmünzen* (Davis et al. 2025) which were controlled by the Persians who were backing the exiled tyrant’s return to Athens. As noted above, the change in ore sources was absolute. Third, the substantial differences in composition and manufacturing technique seem to indicate a new minting authority. Fourth, in addition to the gorgon type, the tyrant continued to mint other types, especially the wheel and pomegranate. Finally, the owls contain overtly Athenian symbolism complete with a written message: ‘[coin] of the Athenians’; a rare phenomenon on archaic Greek coins. A reasonable assumption is that this was a message on behalf of the citizens of the Athenian democracy recently freed from autocratic rule. In contrast, when Hippias did strike obols in exile, he put his own name (ΗΙΠ short for Hippias) on them (Konuk 2024). The contra argument is that Hippias’ later use of the owl type demonstrates that he had come up with it originally and that there is no need to associate a political change with monetary change, especially since the old *Wappenmünzen* fractional coinage continued to be used for some years under the democracy (Davis 2014b) and the method of making the flans for the owls was initially the same as for the *Wappenmünzen*. But there is no actual proof that Hippias struck the first owls, and it is impossible to fully resolve the matter with the information we have.

The discovery and exploitation of third contact Lavrion ores finally gave Athens a reliable ore source. It allowed the state to move from ad hoc minting based on variable supply to larger-scale minting which in turn drove increased monetisation of the economy setting the state on course to become a naval empire. The timing was fortuitous. Had the discovery of the third contact ore source at Lavrion not been made when it was, the ships would never have been built, the Persian invasion probably would not have been defeated, and world history would have been different.

## Conclusions

The extended sampling and improved analytical techniques used in this study, including chemistry, mass spectrometry, and statistical analysis, have demonstrated that the current paradigm which situates the discovery and exploitation of the rich ‘third contact’ mines at Lavrion in Attica late in the sixth century BCE and connects it with the introduction and export of owl type tetradrachms is incorrect. The Pb isotope data show that exogenous silver from a single new or, more likely, homogeneous mixed source was used after the change in type from *Wappenmünzen* to owls combined with gradually increasing quantities of Lavrion ore for most of the coins, though some individual coins had discrete silver sources. The sources for the owls were completely different from those used previously for the *Wappenmünzen*. There are also major compositional differences, notably the near absence of copper and gold and more lead in the owls compared with the *Wappenmünzen*. The new elemental and Pb isotope data of this study confirm evidence derived from primary literary sources that exploitation of the third contact mines occurred shortly before 483/2 BCE, and that it was this event which supplied the funds to support building the fleet. It was only then that Lavrion started to supply most of the silver used for the owls. It is correct to note that owls mostly have a very low percentage of gold, and this must derive from the geology of the silver sources. There is no basis for linking the change in type and ore source with the tyrant Hippias’ alleged monetary reform. There are arguments to suggest that the new owl type was instigated under the democracy. The main significance of this study for understanding Greek history is that Athens did not have access to major funding from domestic Lavrion silver until shortly before the Persian invasion.

## Supplementary Information

Below is the link to the electronic supplementary material.

Supplementary file1 (ZIP 46197 KB)

Supplementary file2 (XLSX 19 KB)

## Data Availability

Data are provided within the manuscript or supplementary information files or in a link to a publicly accessible data base.
