# Supplementary figures and images for: Tyrants, democrats and the first silver ‘owl’ coins of Athens
Source: Archaeol Anthropol Sci. 2025 May 9;17(6):122. doi: 10.1007/s12520-025-02229-z (PMC12064638; doi:10.1007/s12520-025-02229-z)

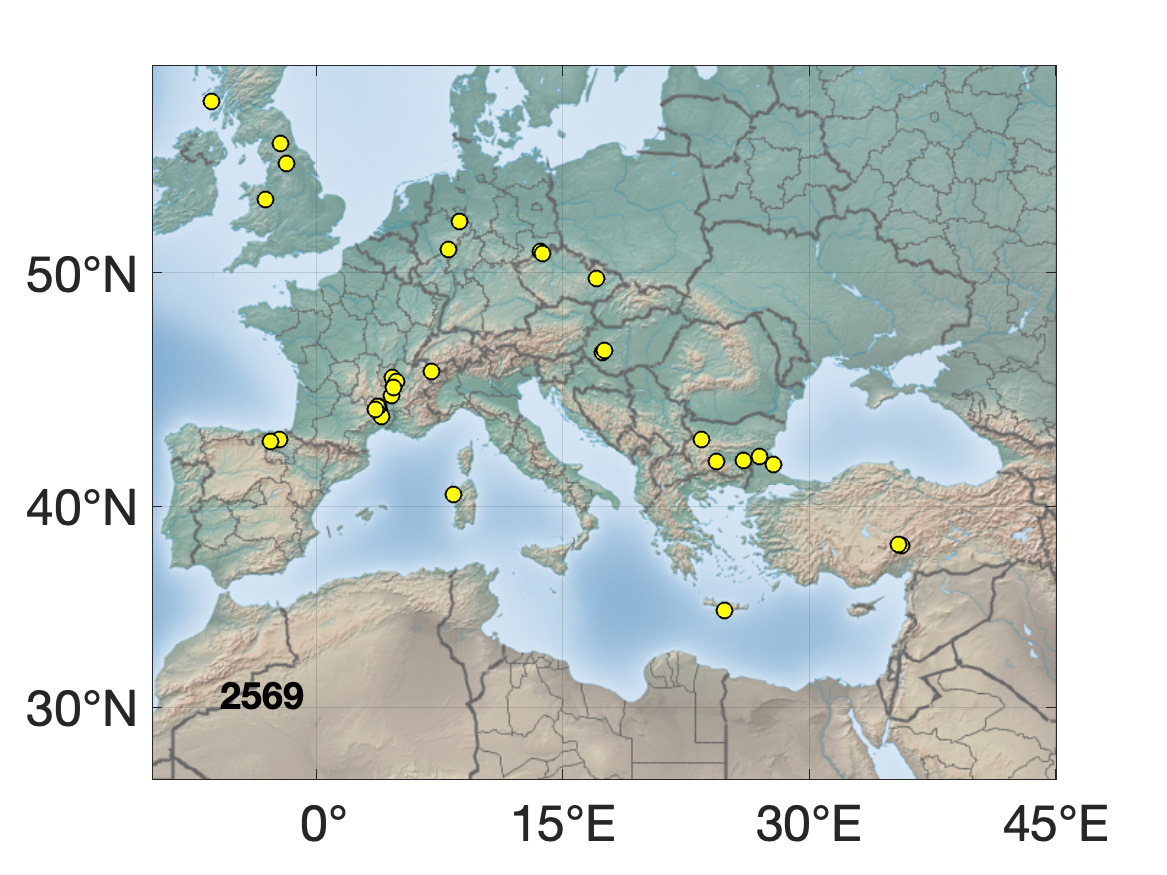

Supplement: Supplementary file 1 — Supplementary file1 (ZIP 46197 KB) [file 12520_2025_2229_MOESM1_ESM.zip › 2569_map_jittered.png]

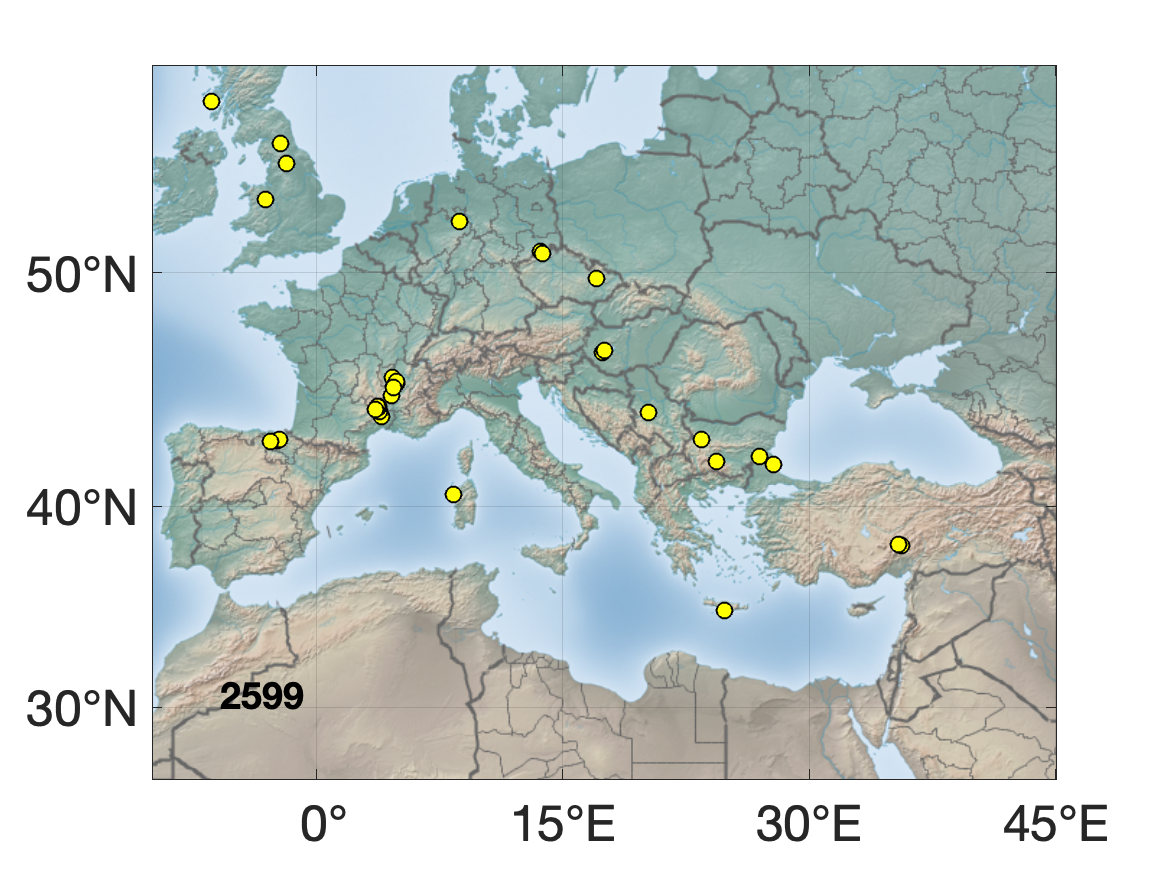

Supplement: Supplementary file 1 — Supplementary file1 (ZIP 46197 KB) [file 12520_2025_2229_MOESM1_ESM.zip › 2599_map_jittered.png]

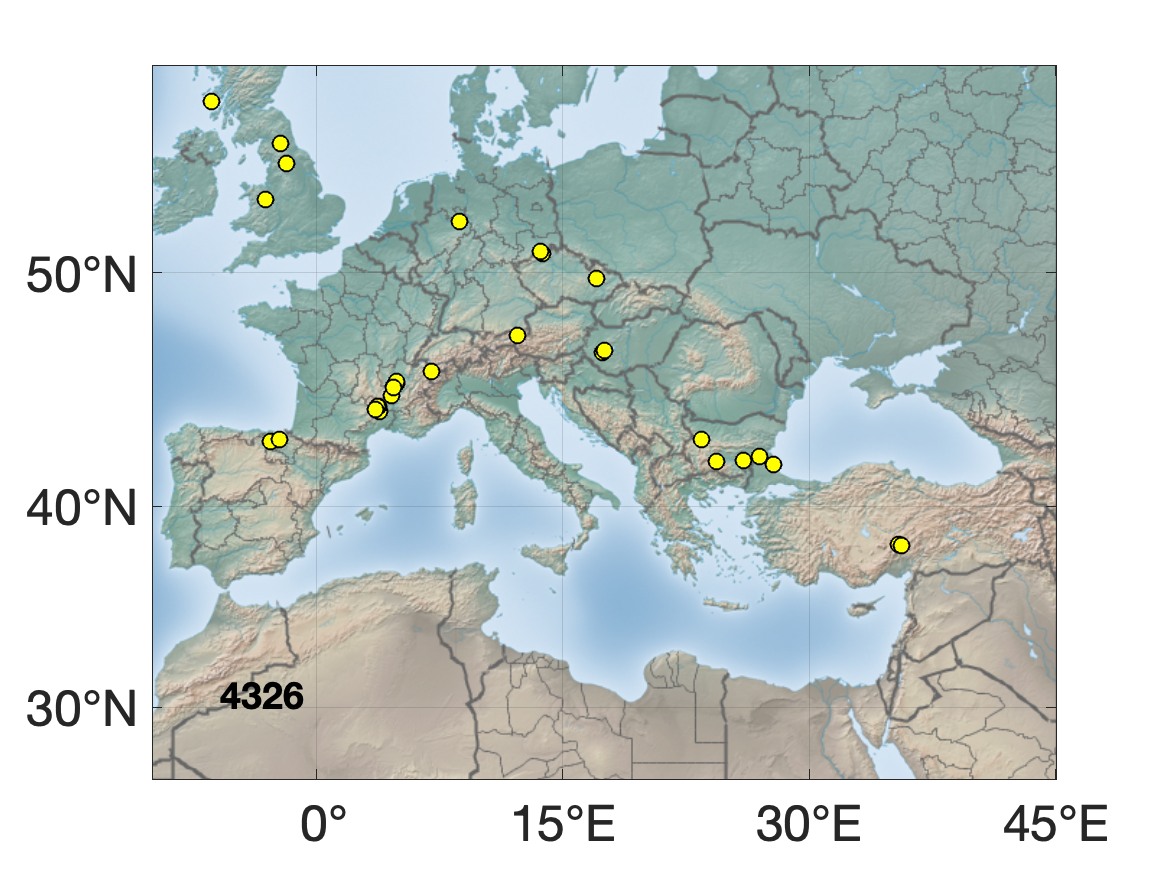

Supplement: Supplementary file 1 — Supplementary file1 (ZIP 46197 KB) [file 12520_2025_2229_MOESM1_ESM.zip › 4326_map_jittered.png]

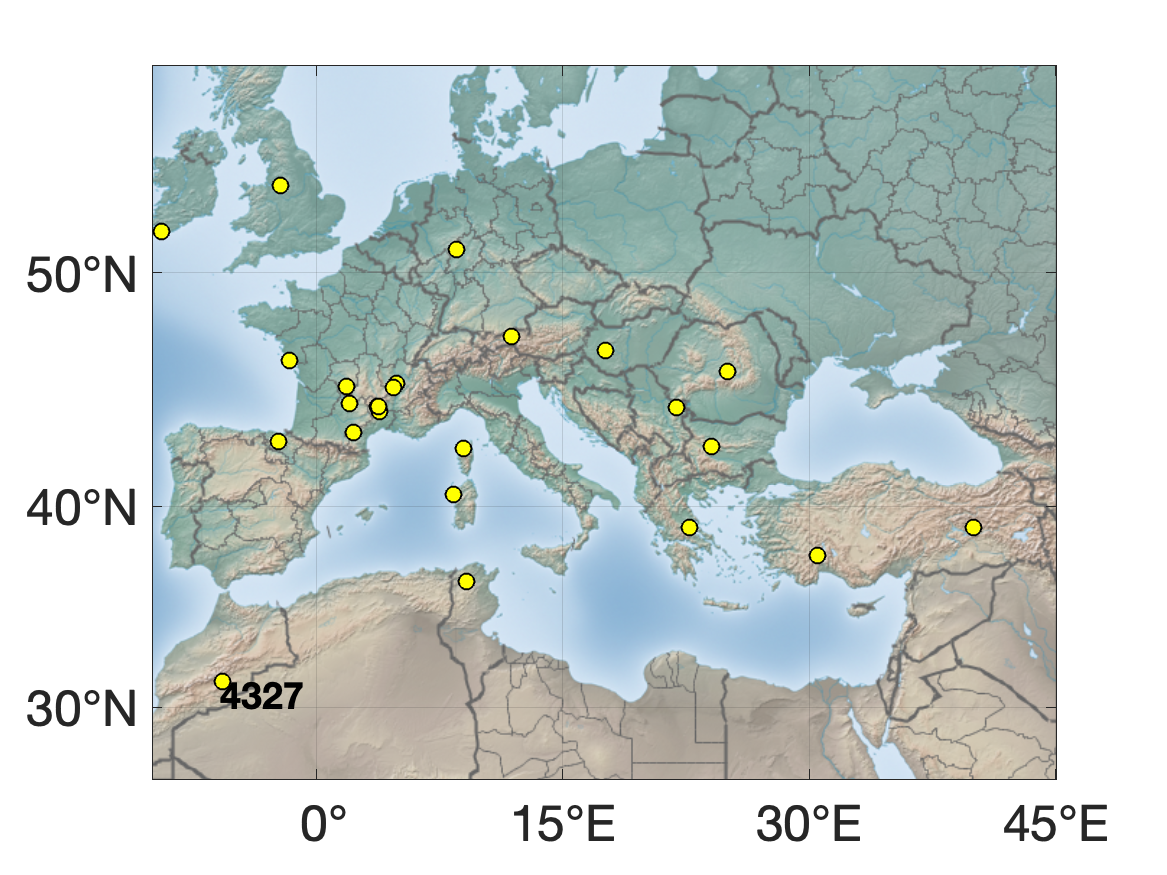

Supplement: Supplementary file 1 — Supplementary file1 (ZIP 46197 KB) [file 12520_2025_2229_MOESM1_ESM.zip › 4327_map_jittered.png]

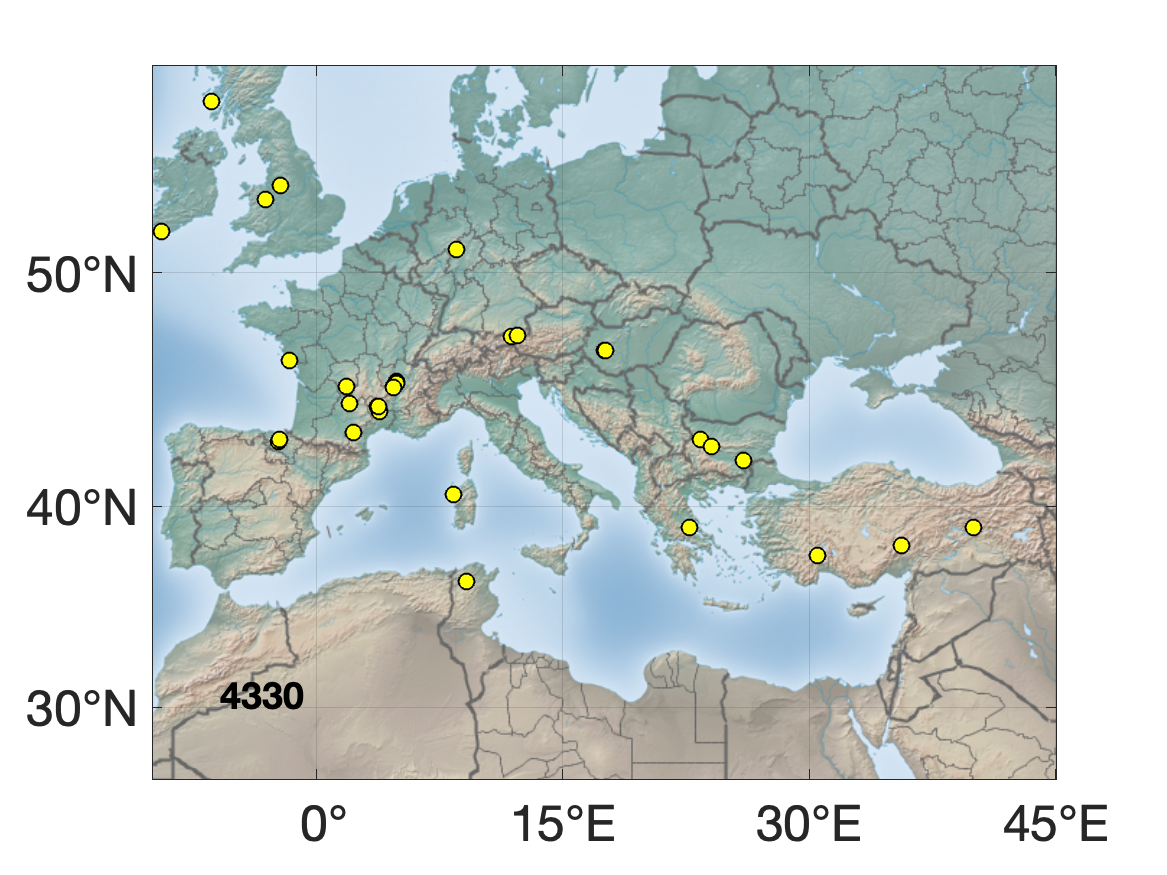

Supplement: Supplementary file 1 — Supplementary file1 (ZIP 46197 KB) [file 12520_2025_2229_MOESM1_ESM.zip › 4330_map_jittered.png]

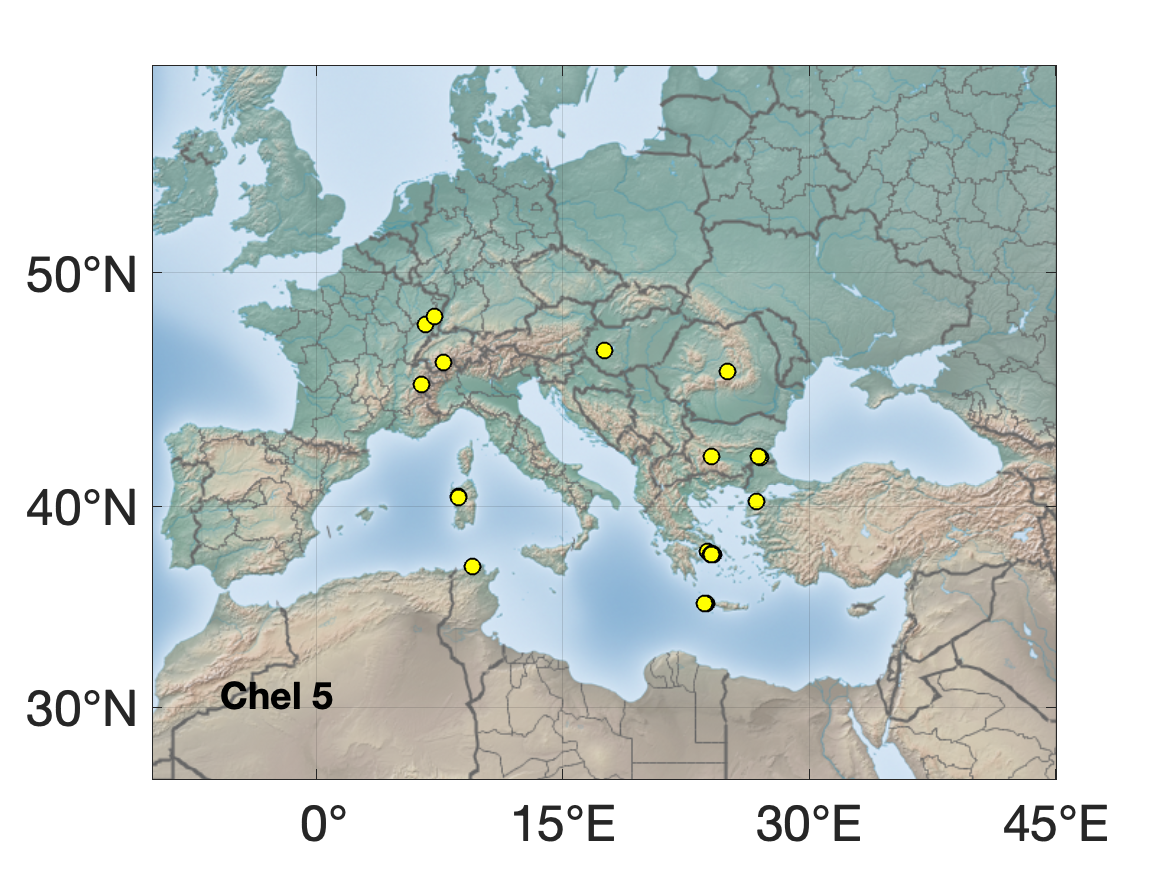

Supplement: Supplementary file 1 — Supplementary file1 (ZIP 46197 KB) [file 12520_2025_2229_MOESM1_ESM.zip › Chel 5_map_jittered.png]

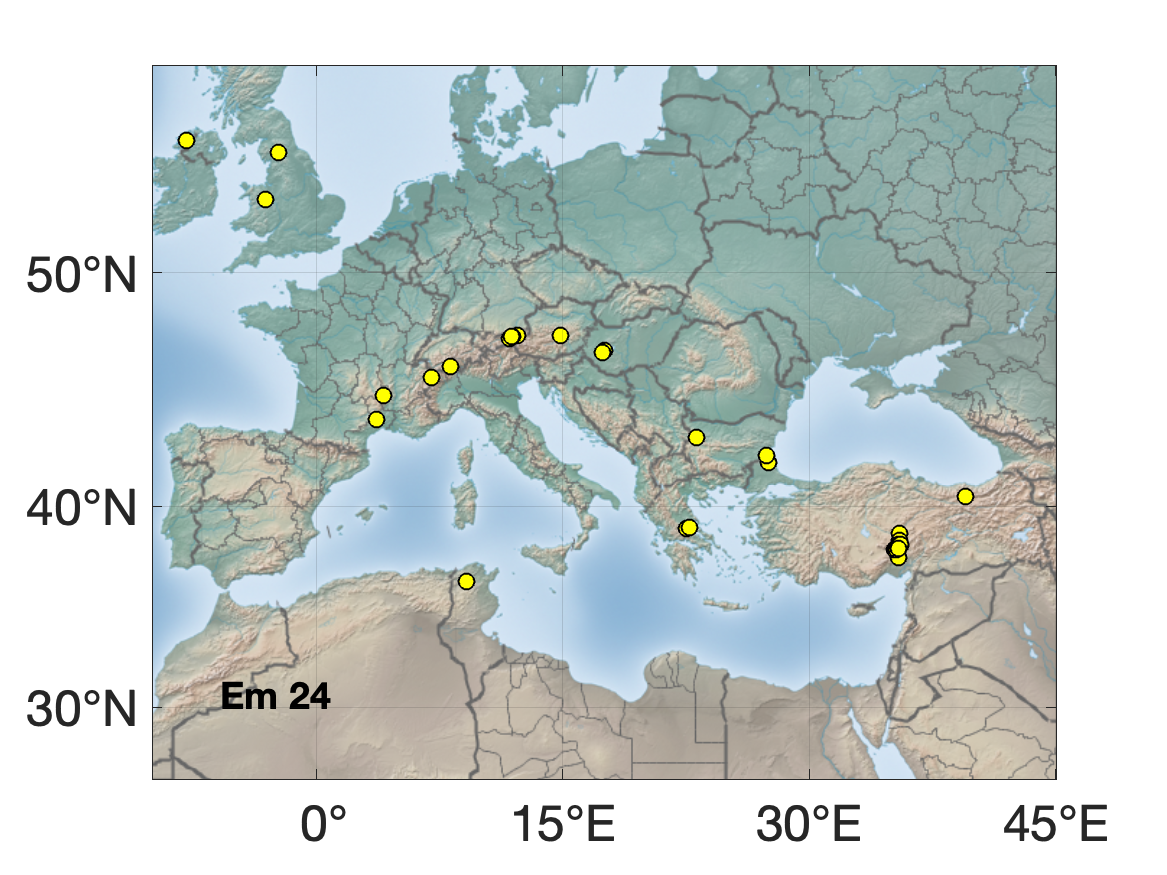

Supplement: Supplementary file 1 — Supplementary file1 (ZIP 46197 KB) [file 12520_2025_2229_MOESM1_ESM.zip › Em 24_map_jittered.png]

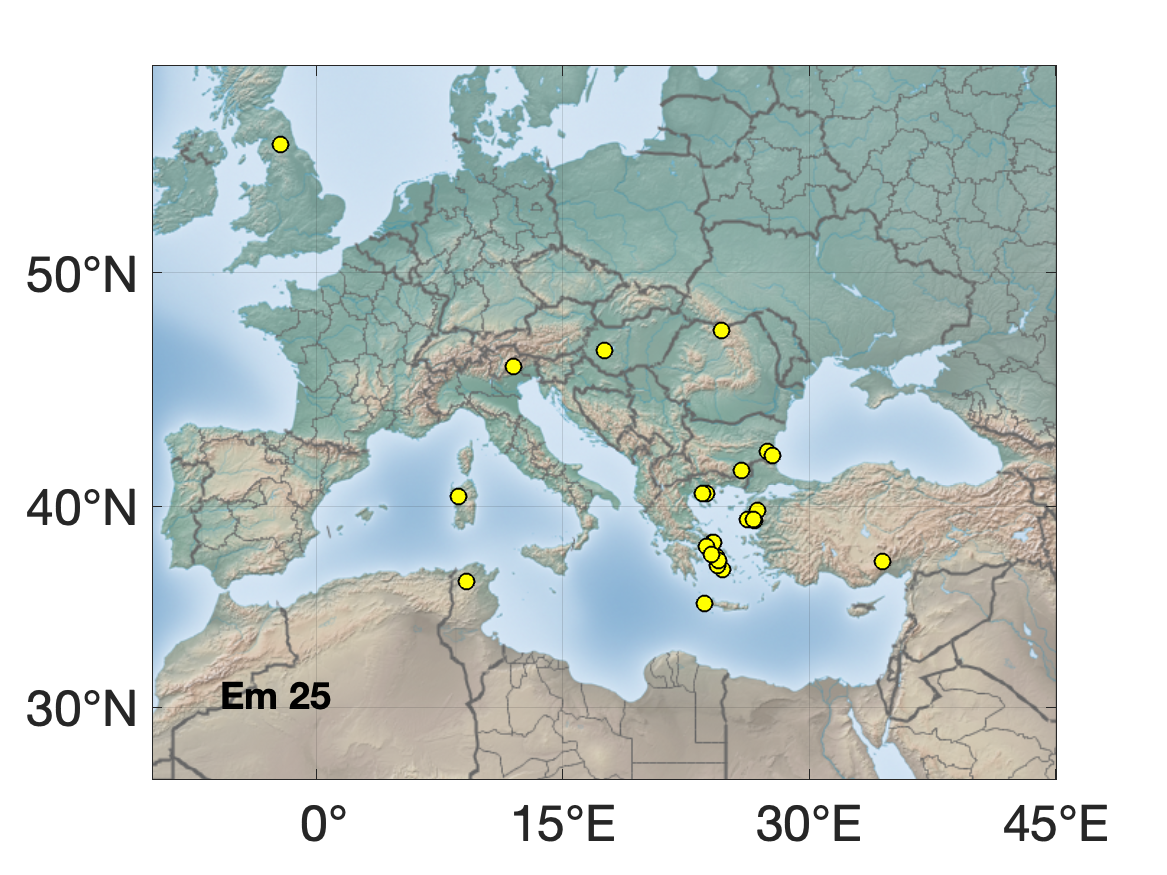

Supplement: Supplementary file 1 — Supplementary file1 (ZIP 46197 KB) [file 12520_2025_2229_MOESM1_ESM.zip › Em 25_map_jittered.png]

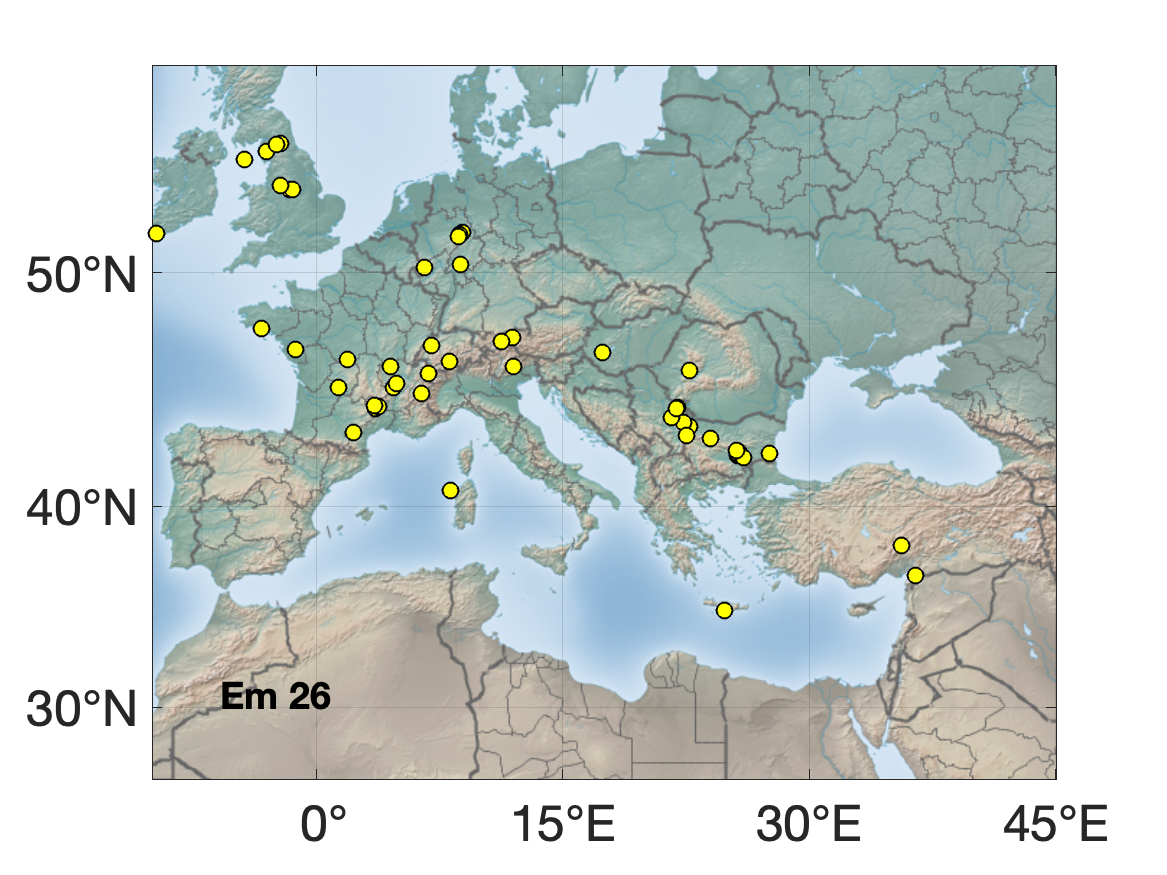

Supplement: Supplementary file 1 — Supplementary file1 (ZIP 46197 KB) [file 12520_2025_2229_MOESM1_ESM.zip › Em 26_map_jittered.png]

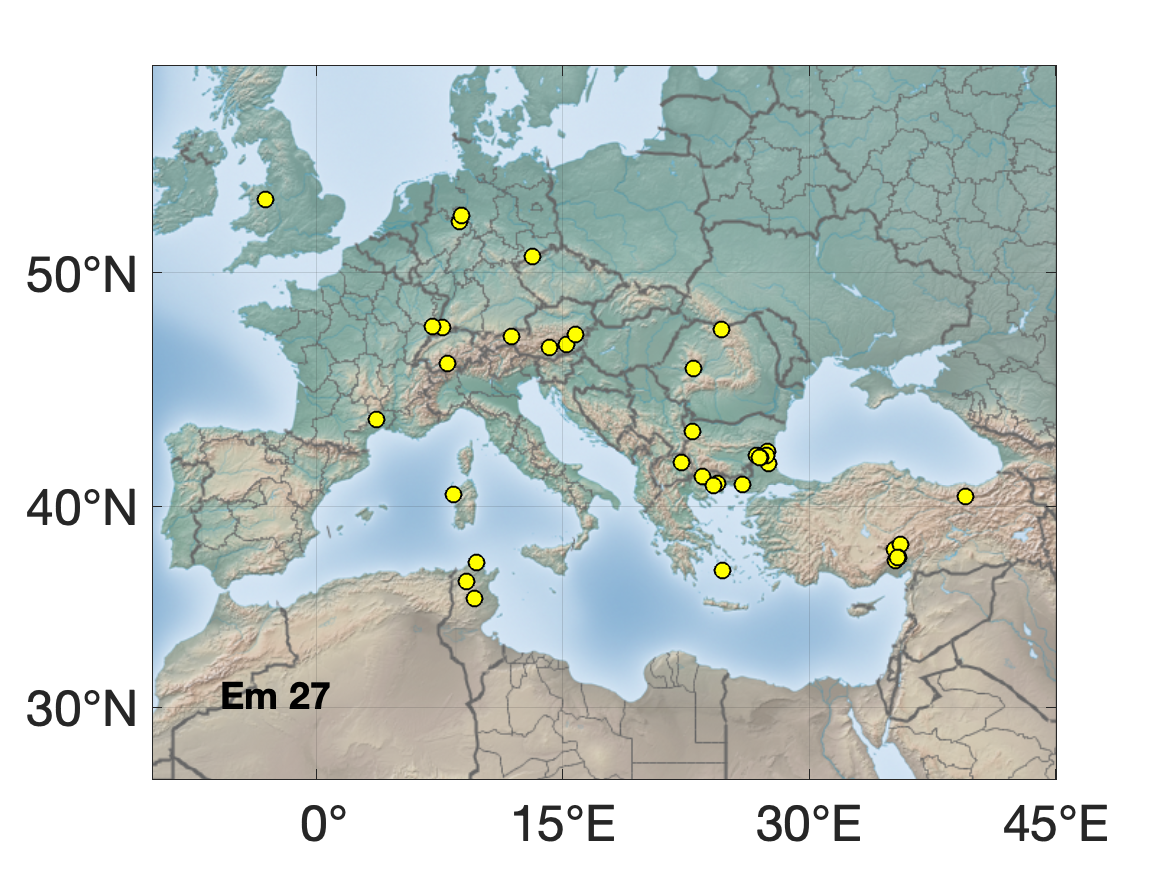

Supplement: Supplementary file 1 — Supplementary file1 (ZIP 46197 KB) [file 12520_2025_2229_MOESM1_ESM.zip › Em 27_map_jittered.png]

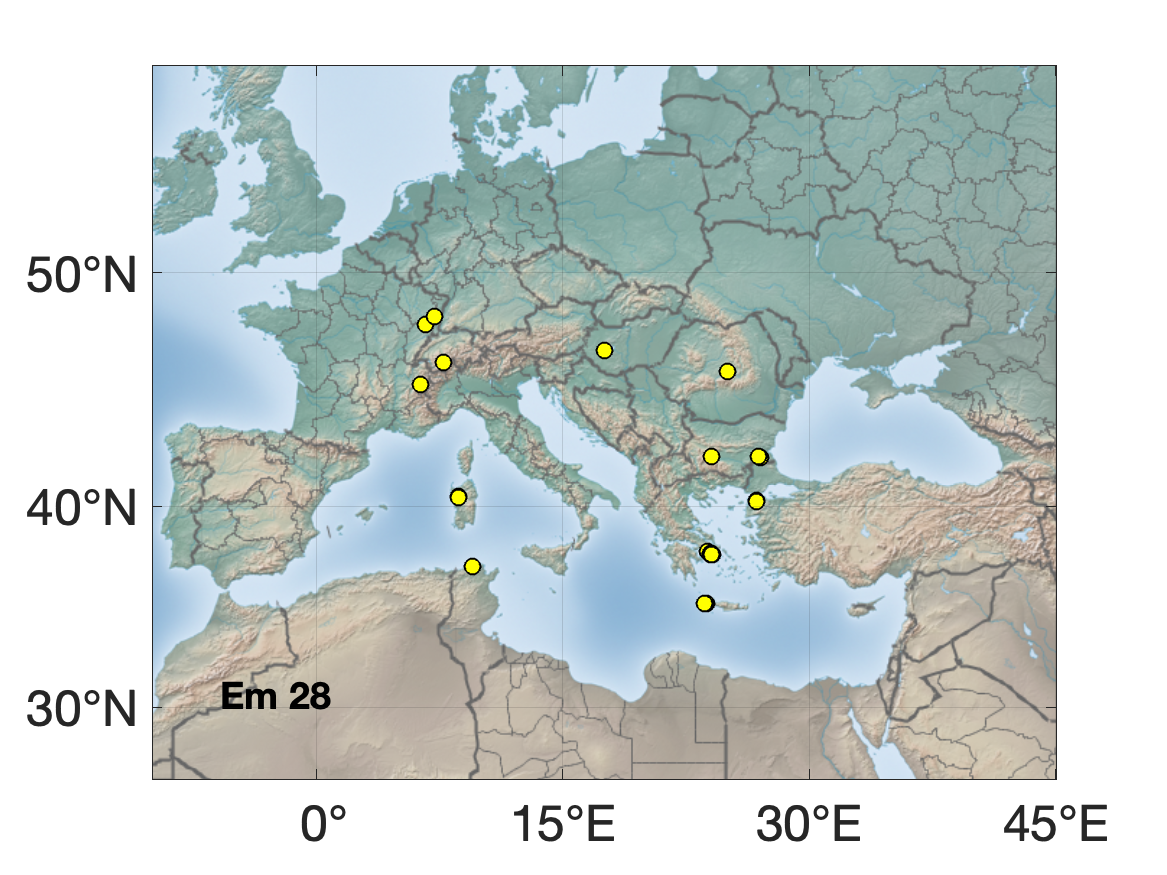

Supplement: Supplementary file 1 — Supplementary file1 (ZIP 46197 KB) [file 12520_2025_2229_MOESM1_ESM.zip › Em 28_map_jittered.png]

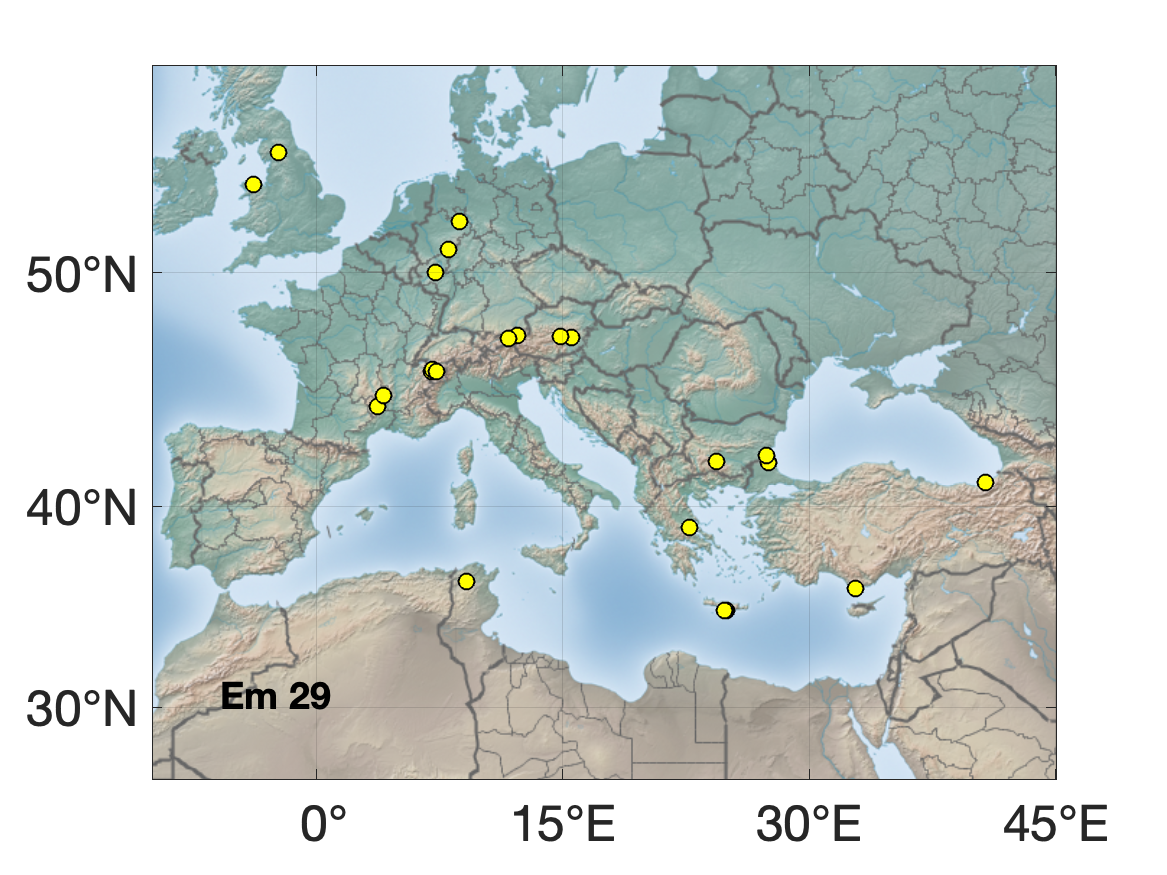

Supplement: Supplementary file 1 — Supplementary file1 (ZIP 46197 KB) [file 12520_2025_2229_MOESM1_ESM.zip › Em 29_map_jittered.png]

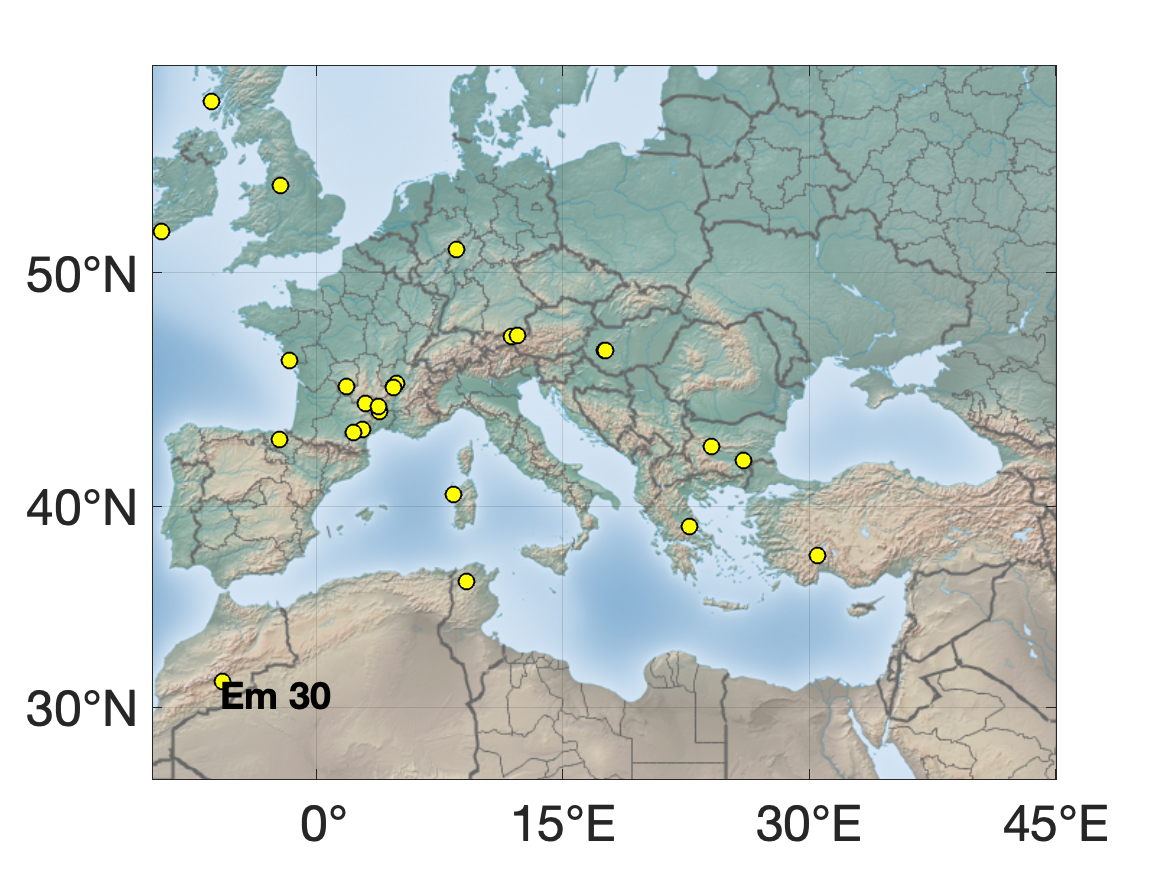

Supplement: Supplementary file 1 — Supplementary file1 (ZIP 46197 KB) [file 12520_2025_2229_MOESM1_ESM.zip › Em 30_map_jittered.png]

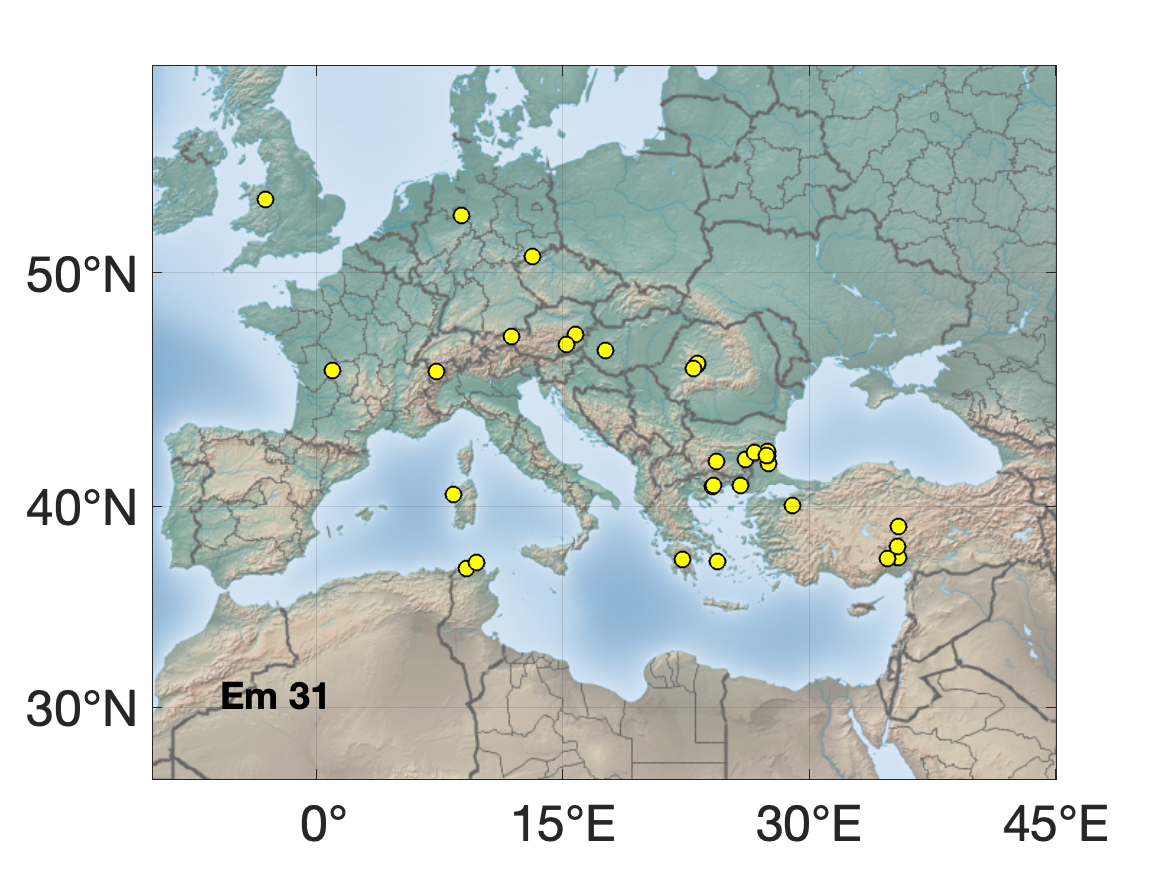

Supplement: Supplementary file 1 — Supplementary file1 (ZIP 46197 KB) [file 12520_2025_2229_MOESM1_ESM.zip › Em 31_map_jittered.png]

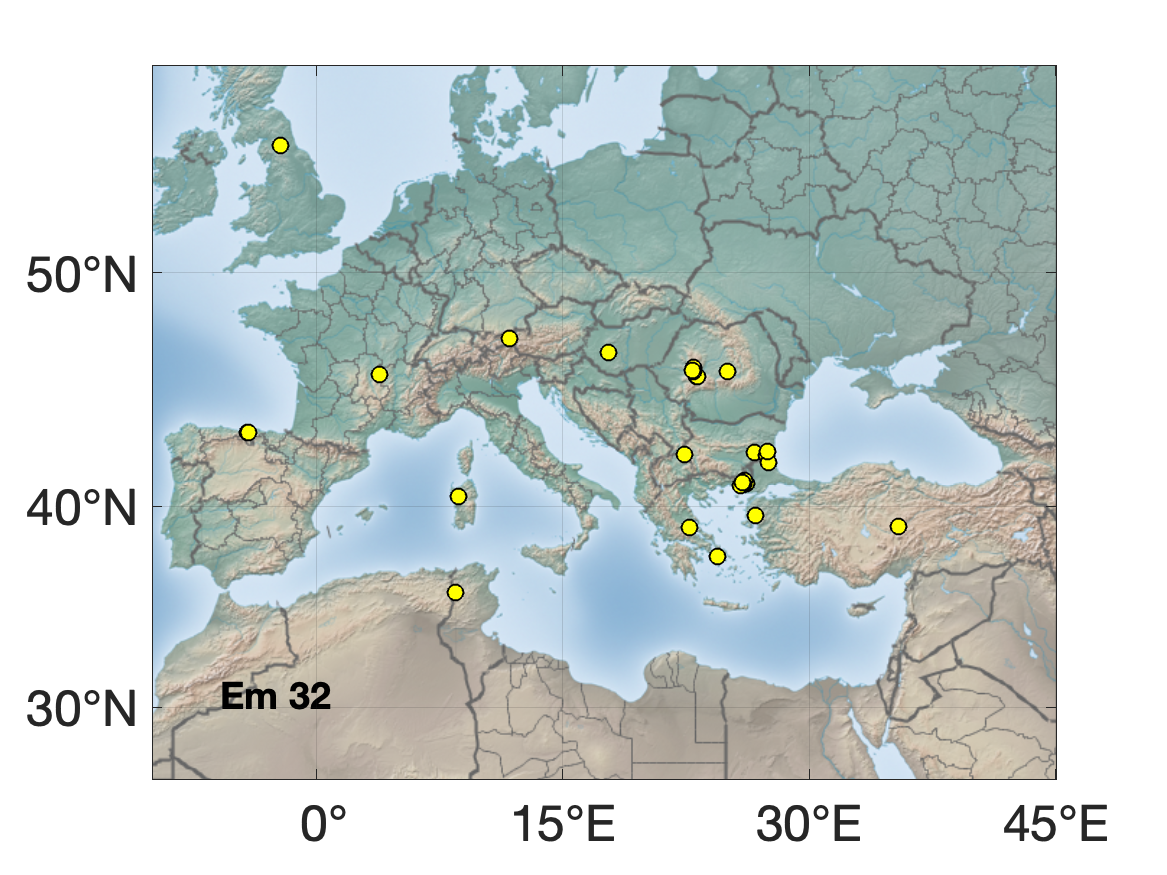

Supplement: Supplementary file 1 — Supplementary file1 (ZIP 46197 KB) [file 12520_2025_2229_MOESM1_ESM.zip › Em 32_map_jittered.png]

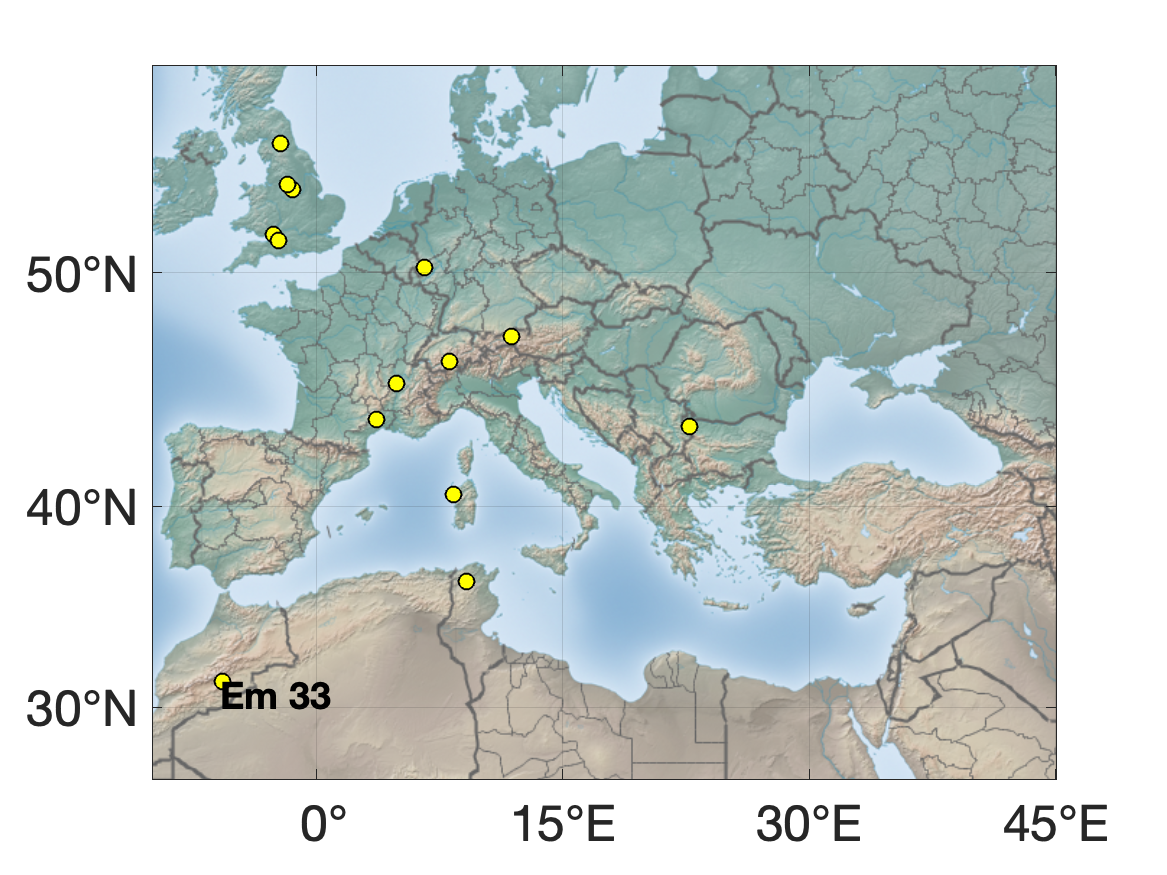

Supplement: Supplementary file 1 — Supplementary file1 (ZIP 46197 KB) [file 12520_2025_2229_MOESM1_ESM.zip › Em 33_map_jittered.png]

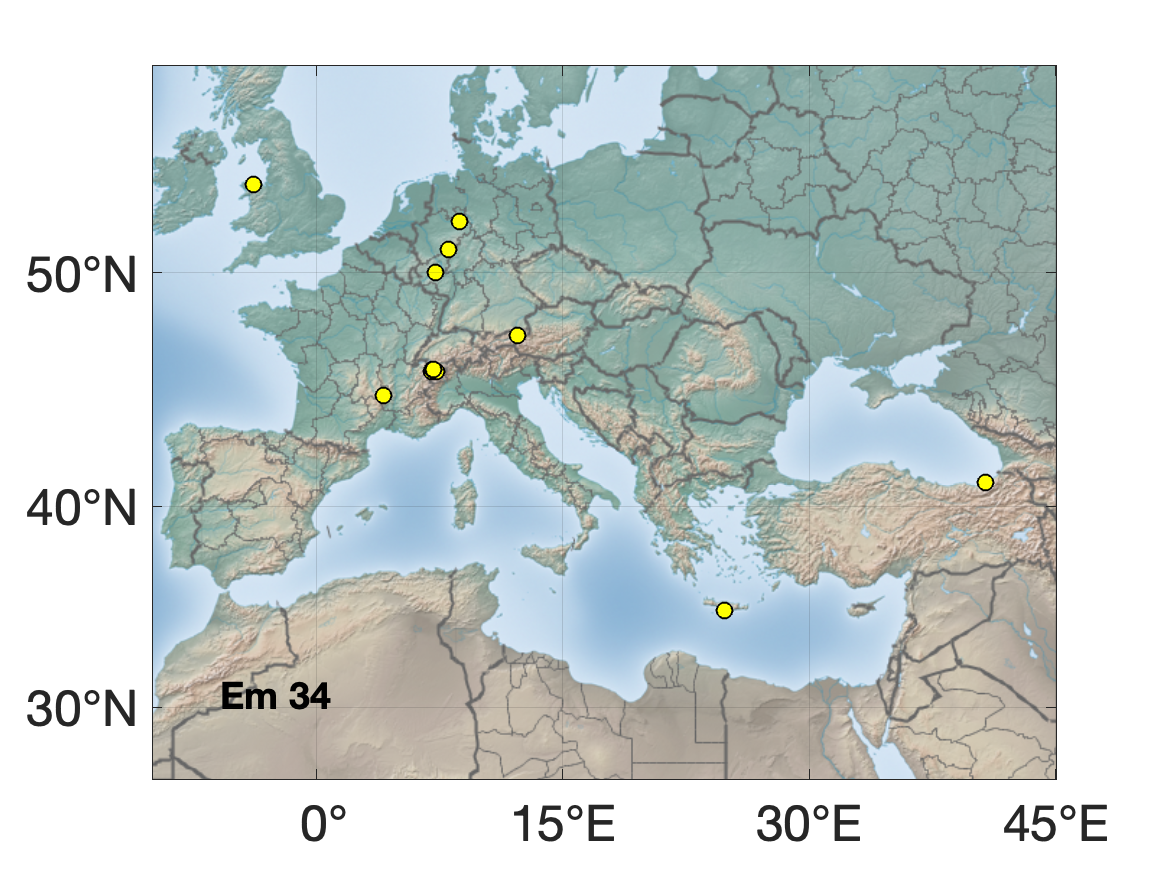

Supplement: Supplementary file 1 — Supplementary file1 (ZIP 46197 KB) [file 12520_2025_2229_MOESM1_ESM.zip › Em 34_map_jittered.png]

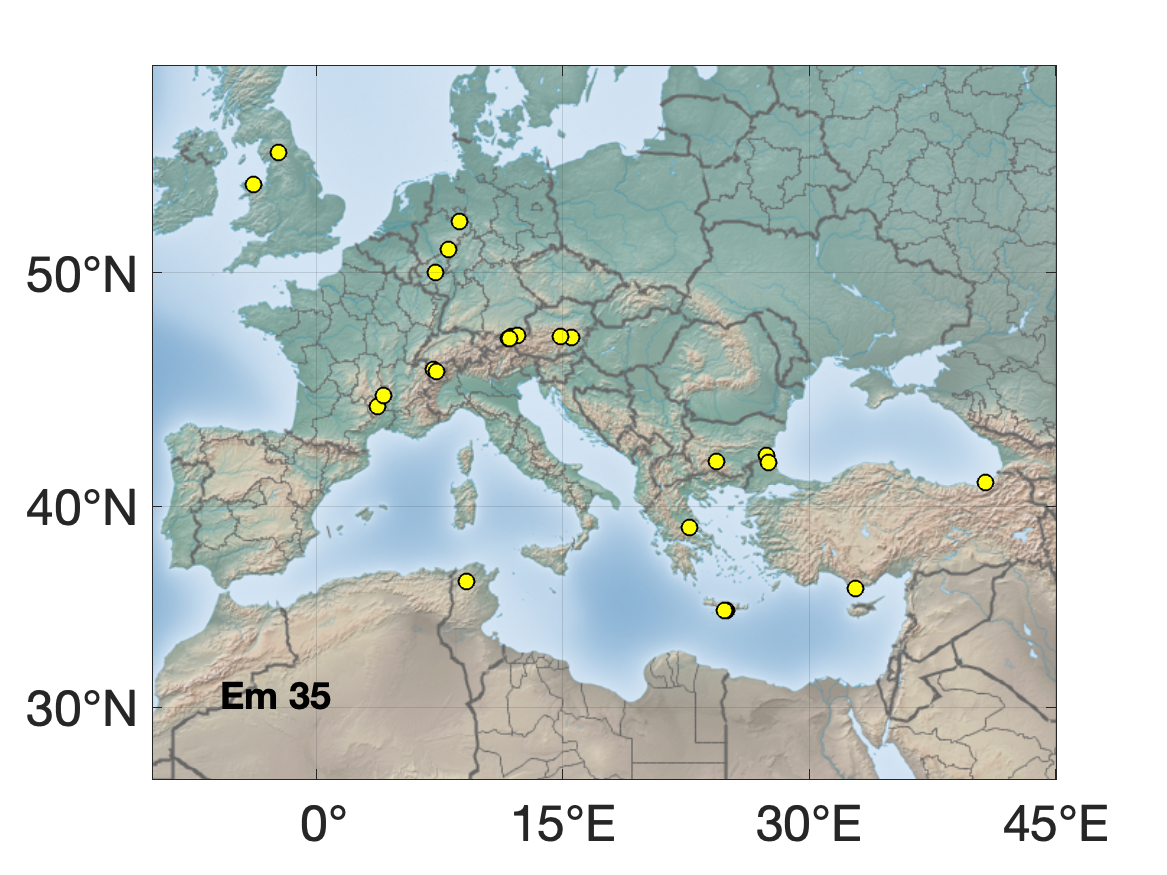

Supplement: Supplementary file 1 — Supplementary file1 (ZIP 46197 KB) [file 12520_2025_2229_MOESM1_ESM.zip › Em 35_map_jittered.png]

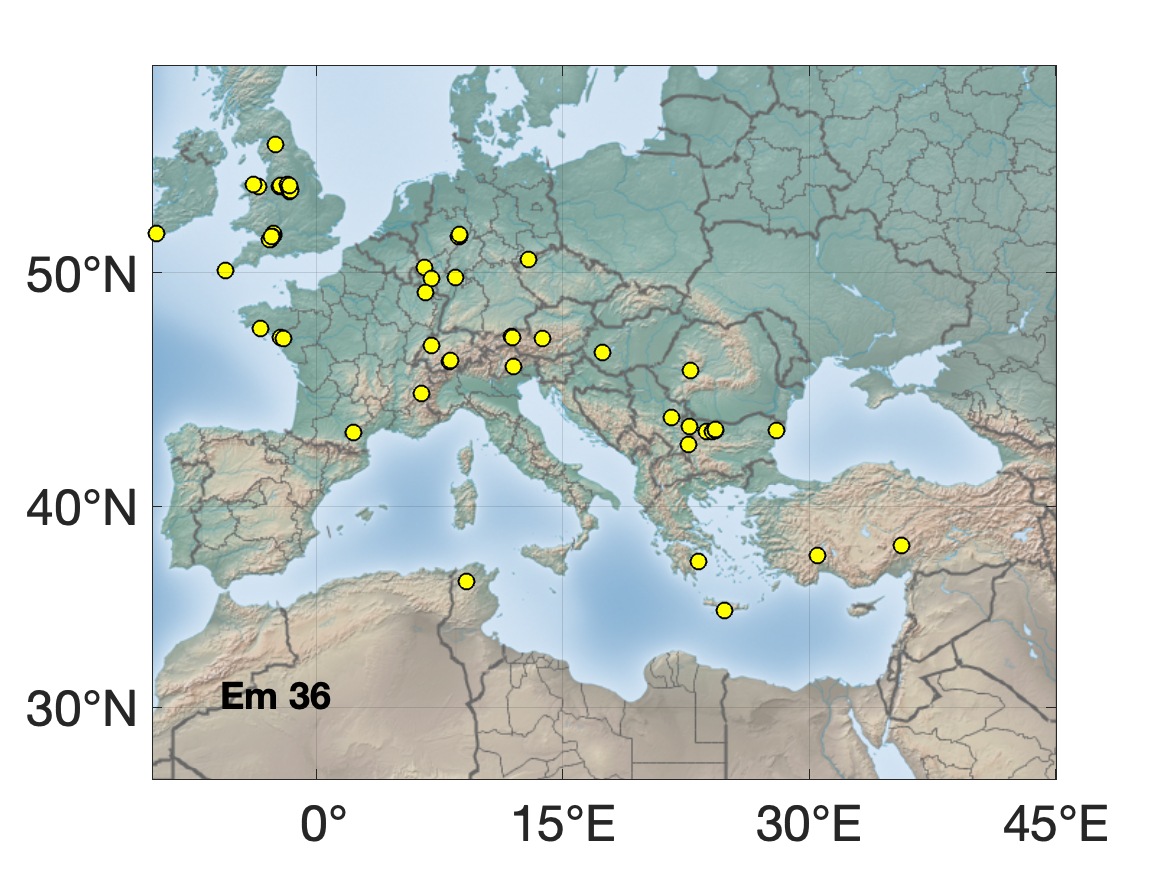

Supplement: Supplementary file 1 — Supplementary file1 (ZIP 46197 KB) [file 12520_2025_2229_MOESM1_ESM.zip › Em 36_map_jittered.png]

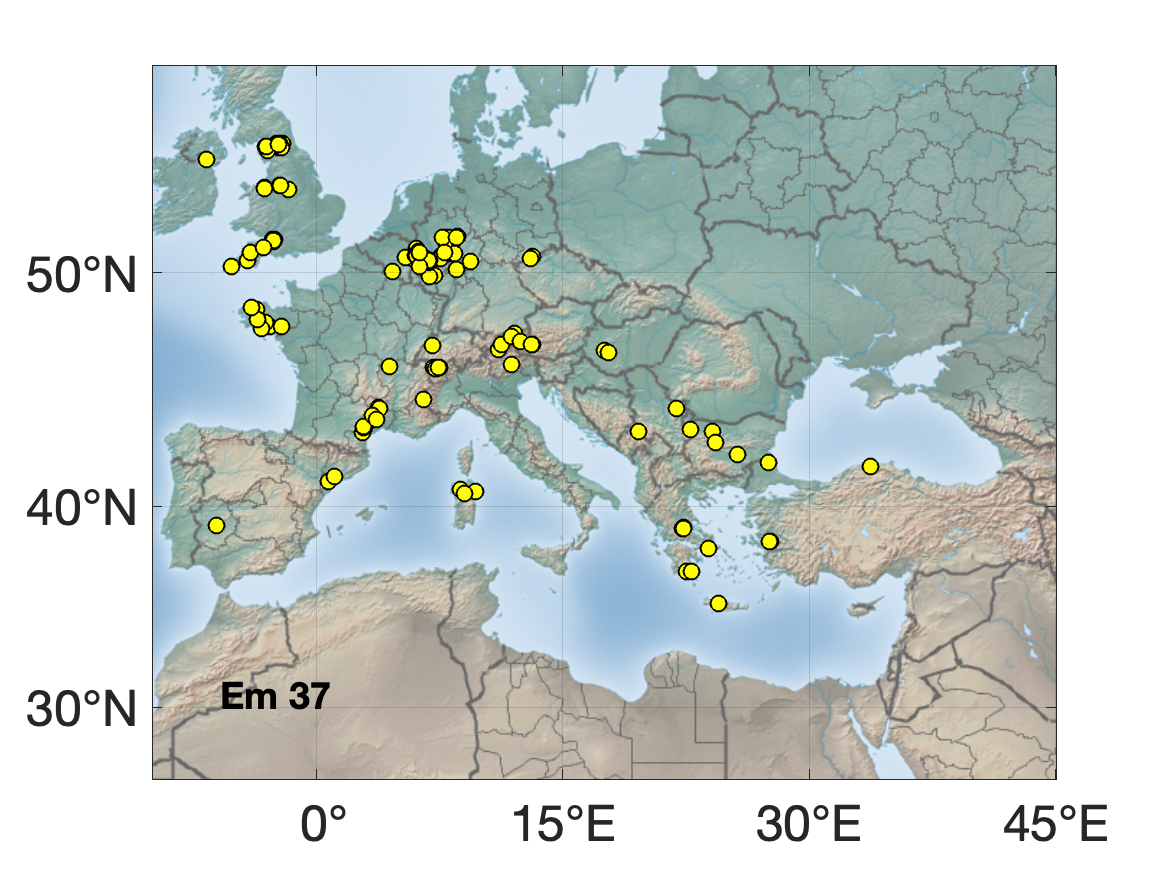

Supplement: Supplementary file 1 — Supplementary file1 (ZIP 46197 KB) [file 12520_2025_2229_MOESM1_ESM.zip › Em 37_map_jittered.png]

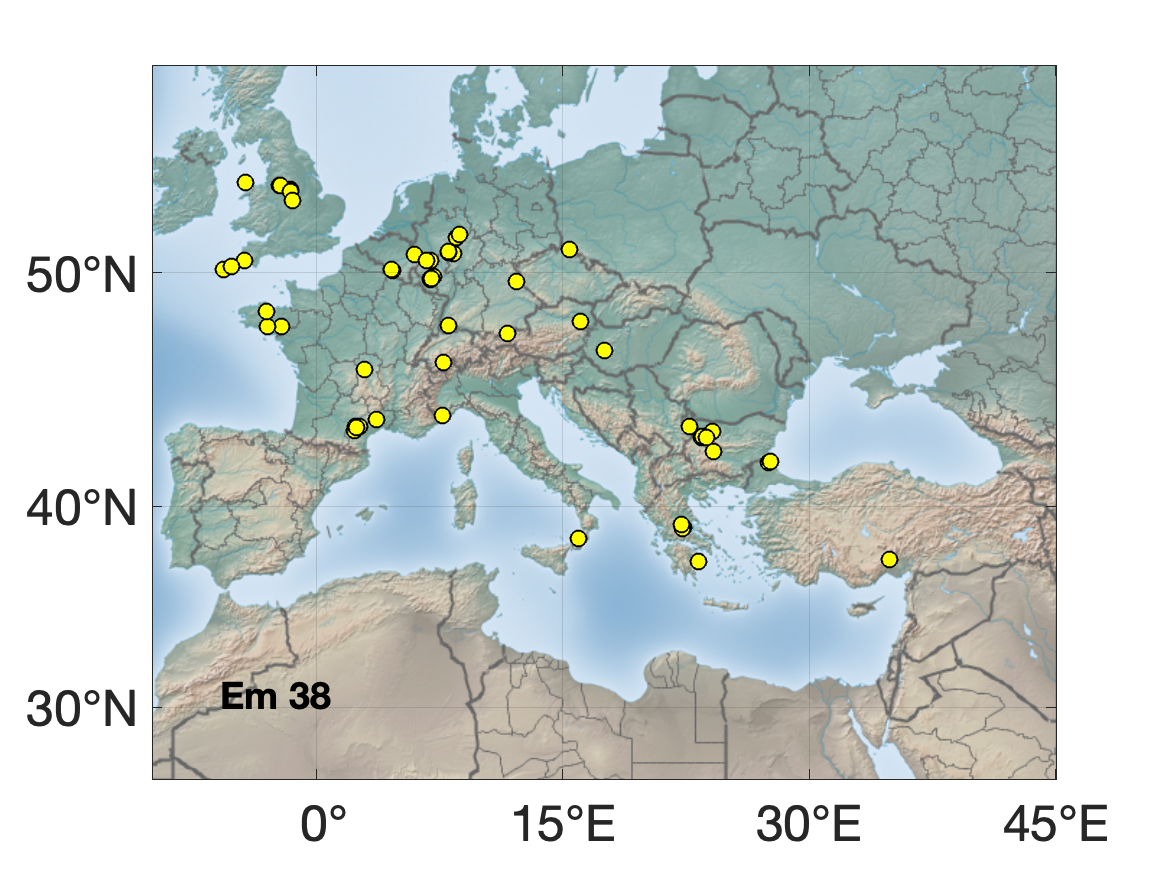

Supplement: Supplementary file 1 — Supplementary file1 (ZIP 46197 KB) [file 12520_2025_2229_MOESM1_ESM.zip › Em 38_map_jittered.png]

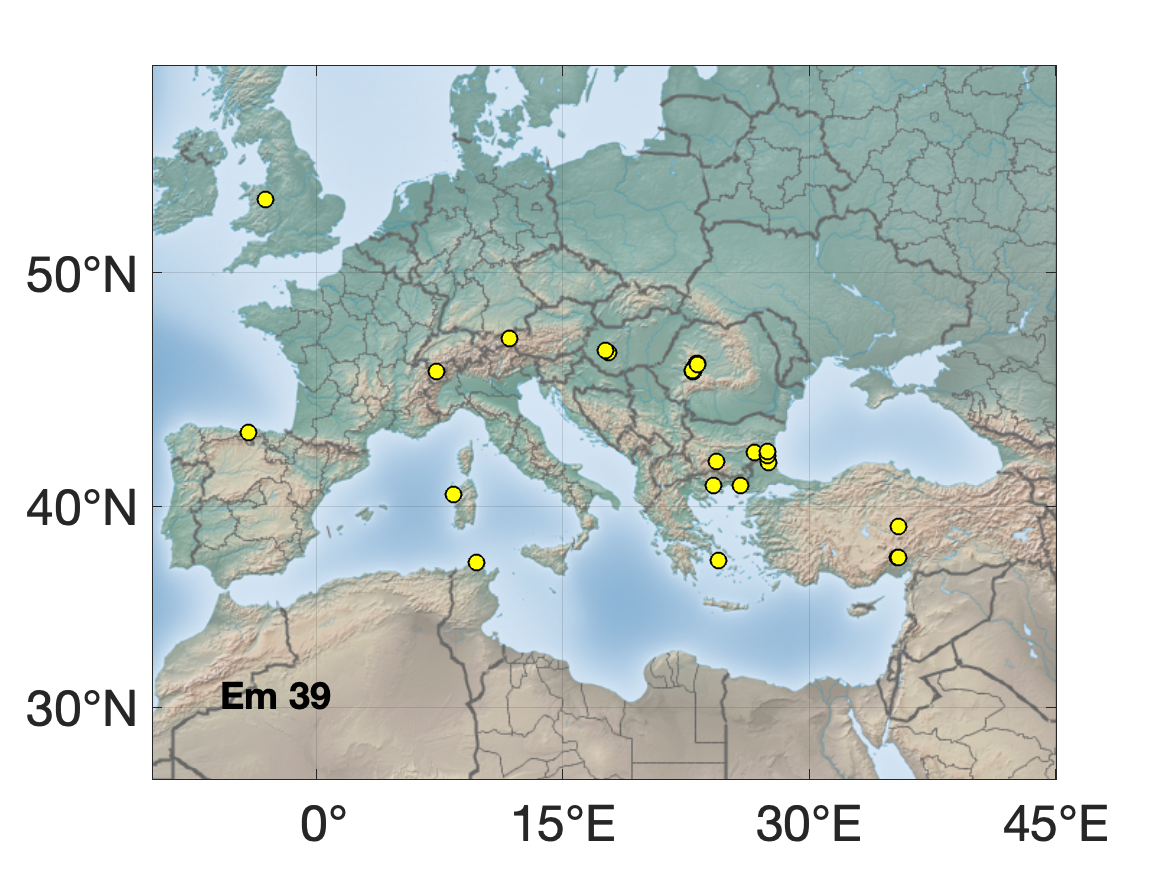

Supplement: Supplementary file 1 — Supplementary file1 (ZIP 46197 KB) [file 12520_2025_2229_MOESM1_ESM.zip › Em 39_map_jittered.png]

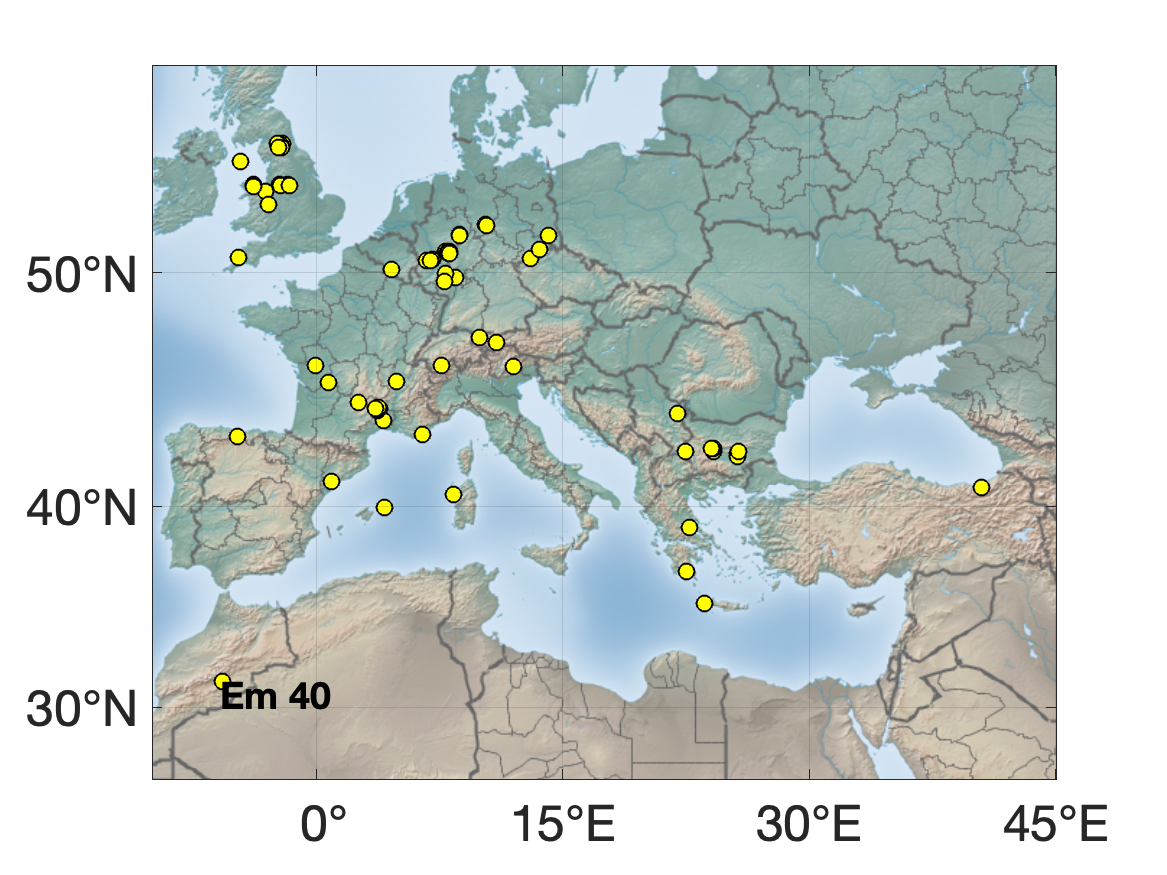

Supplement: Supplementary file 1 — Supplementary file1 (ZIP 46197 KB) [file 12520_2025_2229_MOESM1_ESM.zip › Em 40_map_jittered.png]

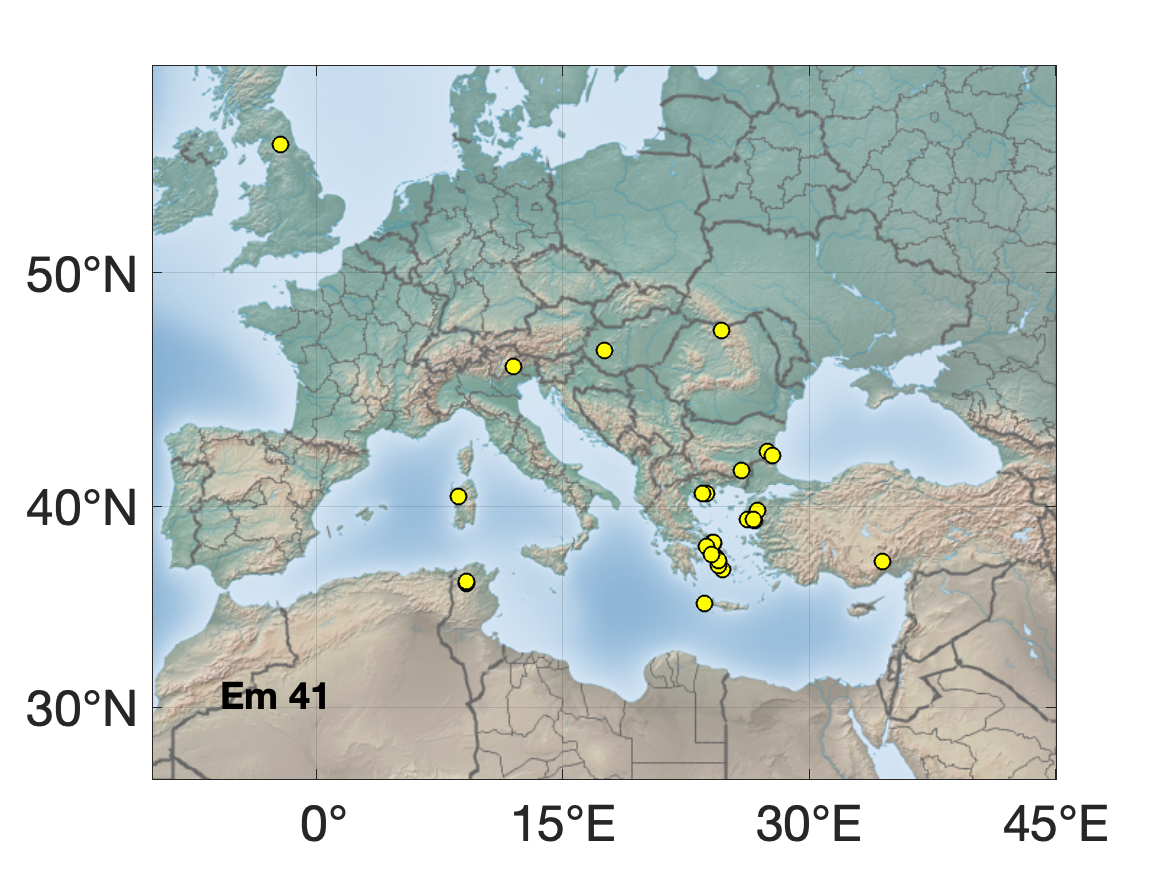

Supplement: Supplementary file 1 — Supplementary file1 (ZIP 46197 KB) [file 12520_2025_2229_MOESM1_ESM.zip › Em 41_map_jittered.png]

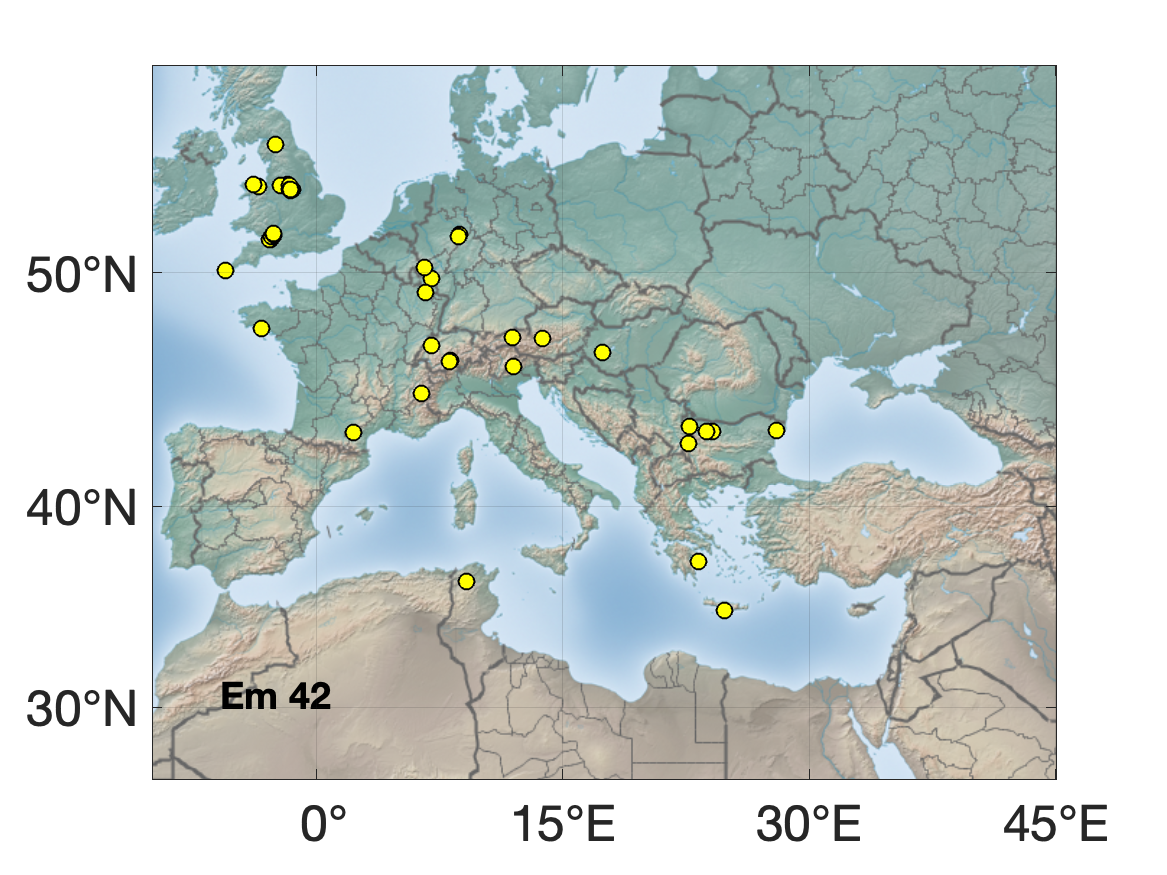

Supplement: Supplementary file 1 — Supplementary file1 (ZIP 46197 KB) [file 12520_2025_2229_MOESM1_ESM.zip › Em 42_map_jittered.png]

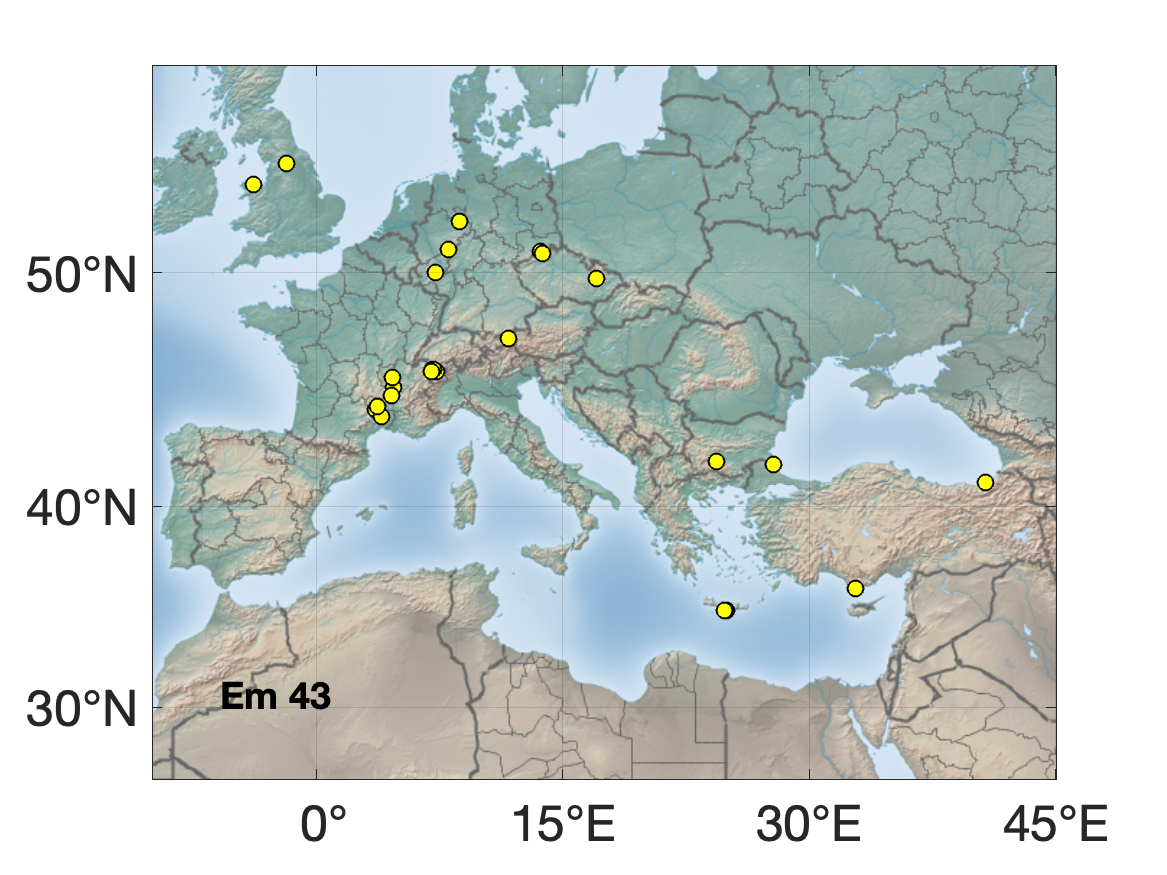

Supplement: Supplementary file 1 — Supplementary file1 (ZIP 46197 KB) [file 12520_2025_2229_MOESM1_ESM.zip › Em 43_map_jittered.png]

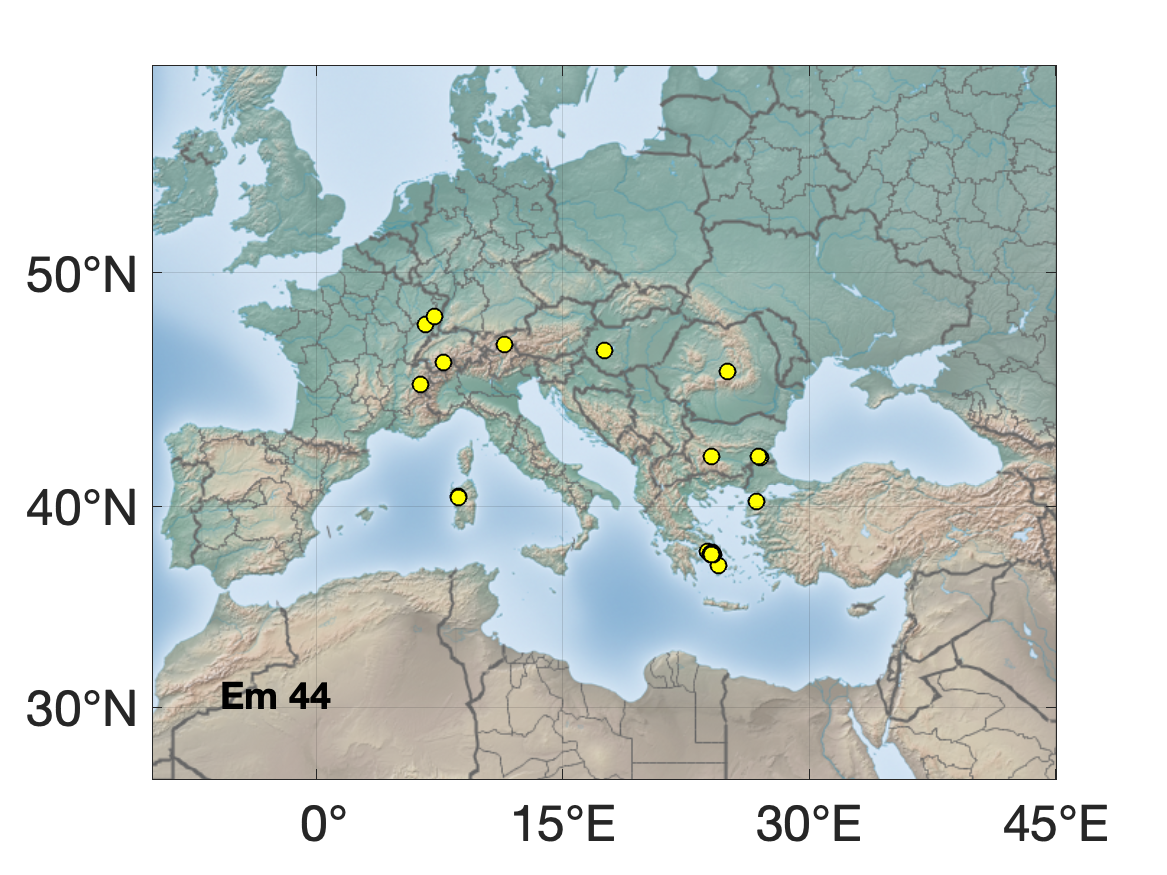

Supplement: Supplementary file 1 — Supplementary file1 (ZIP 46197 KB) [file 12520_2025_2229_MOESM1_ESM.zip › Em 44_map_jittered.png]

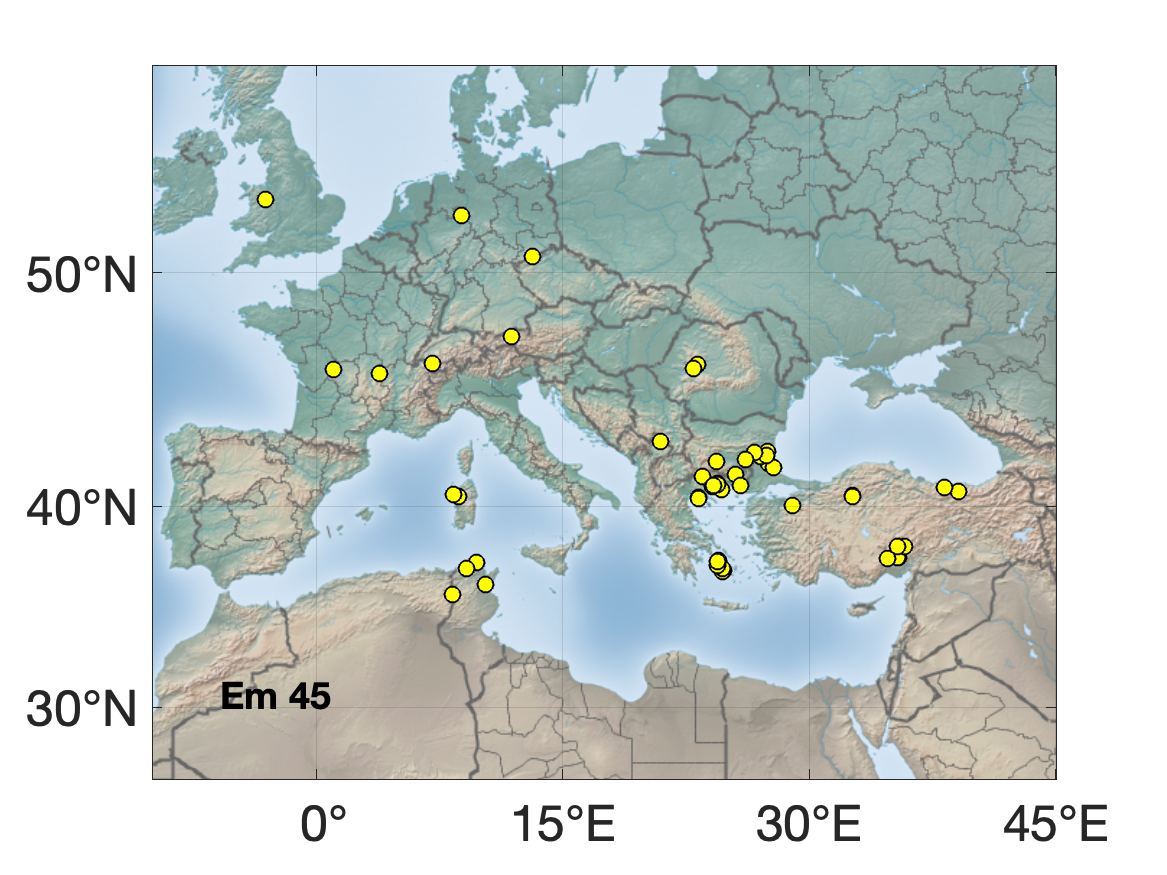

Supplement: Supplementary file 1 — Supplementary file1 (ZIP 46197 KB) [file 12520_2025_2229_MOESM1_ESM.zip › Em 45_map_jittered.png]

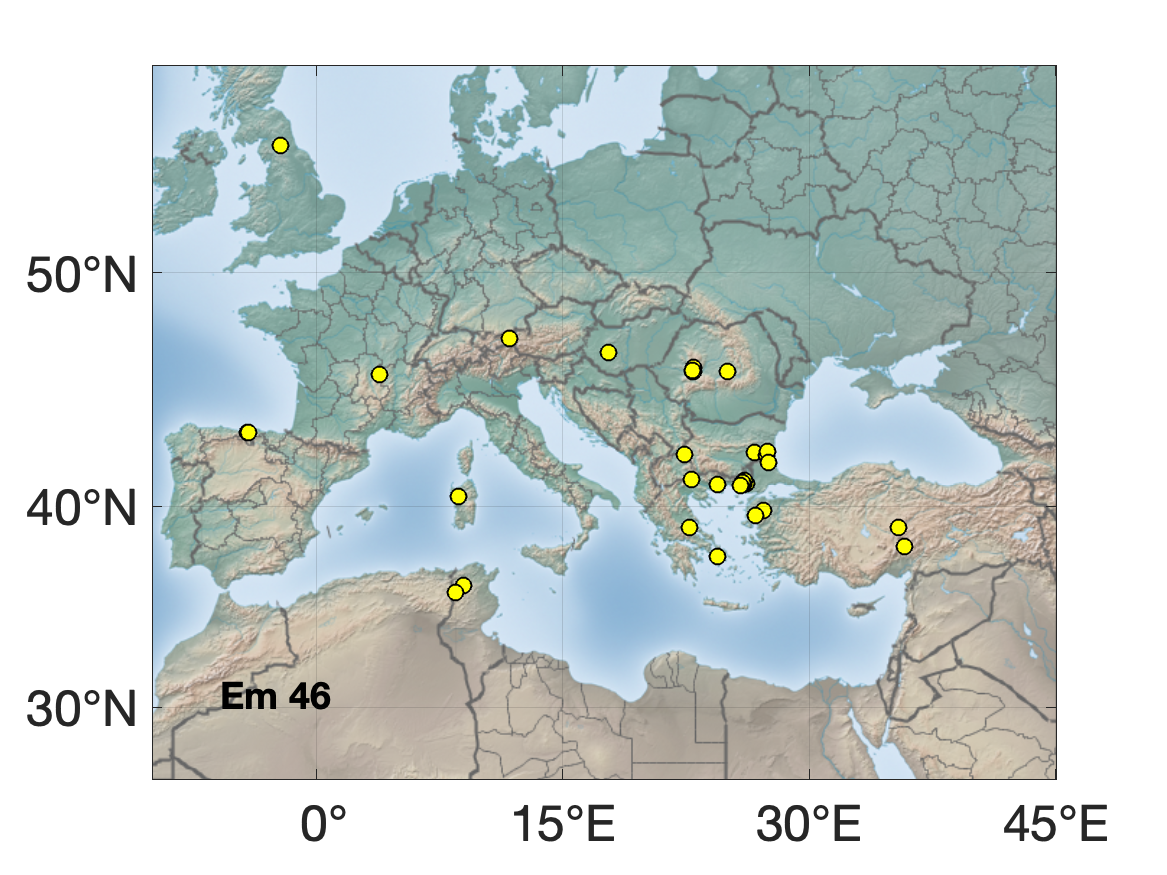

Supplement: Supplementary file 1 — Supplementary file1 (ZIP 46197 KB) [file 12520_2025_2229_MOESM1_ESM.zip › Em 46_map_jittered.png]

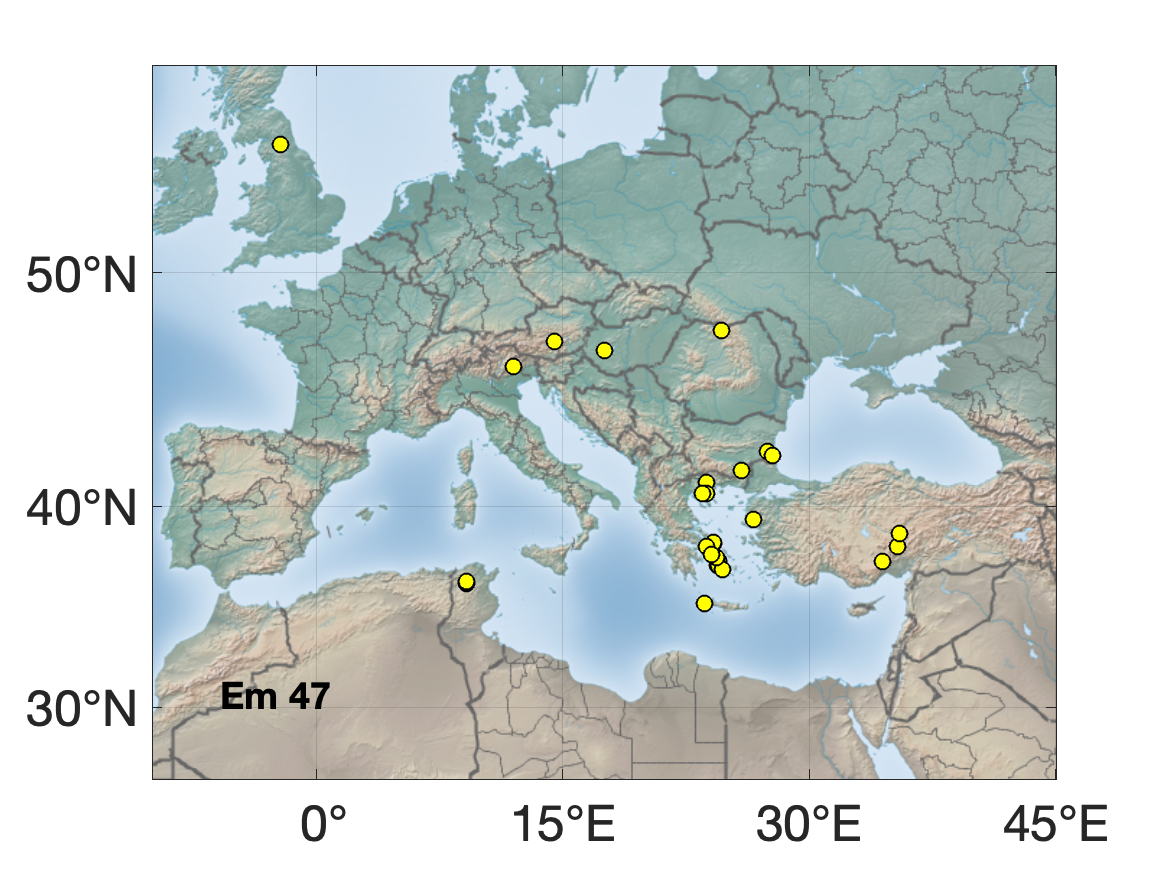

Supplement: Supplementary file 1 — Supplementary file1 (ZIP 46197 KB) [file 12520_2025_2229_MOESM1_ESM.zip › Em 47_map_jittered.png]

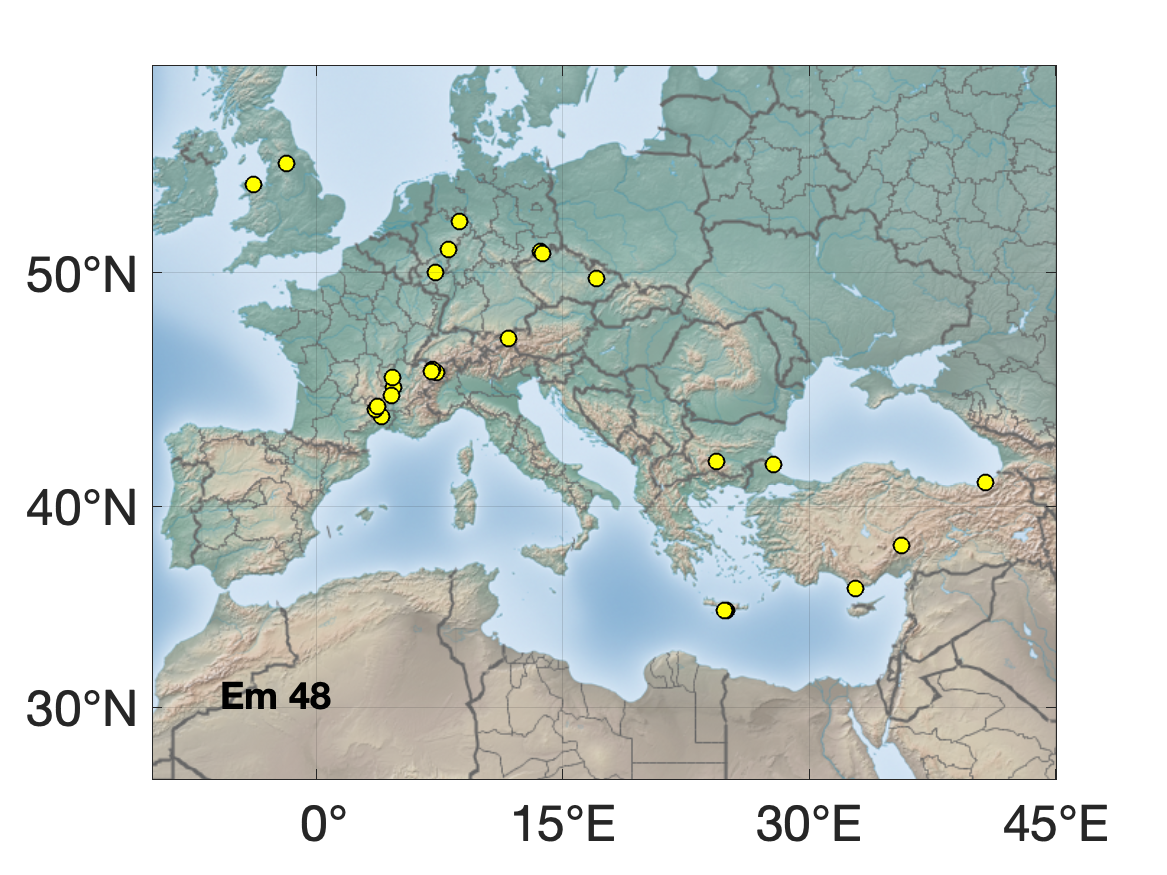

Supplement: Supplementary file 1 — Supplementary file1 (ZIP 46197 KB) [file 12520_2025_2229_MOESM1_ESM.zip › Em 48_map_jittered.png]

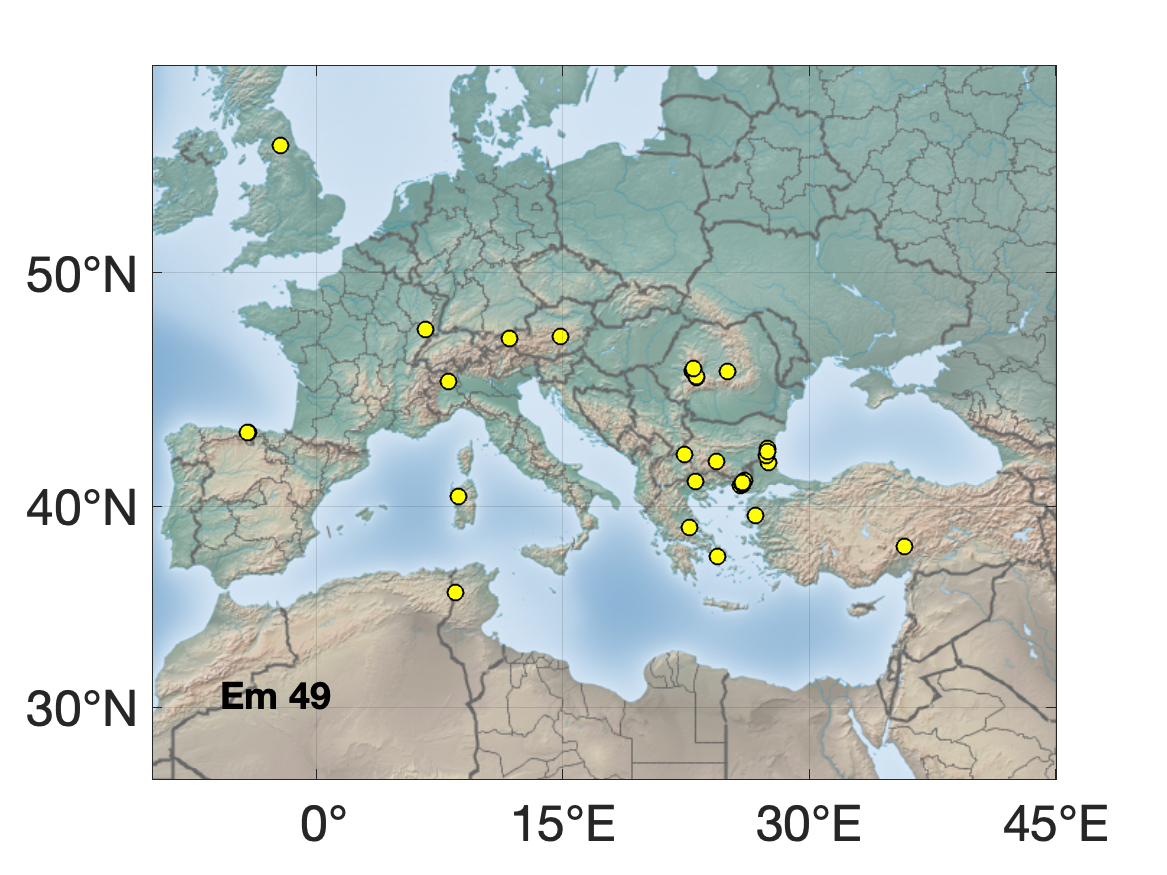

Supplement: Supplementary file 1 — Supplementary file1 (ZIP 46197 KB) [file 12520_2025_2229_MOESM1_ESM.zip › Em 49_map_jittered.png]

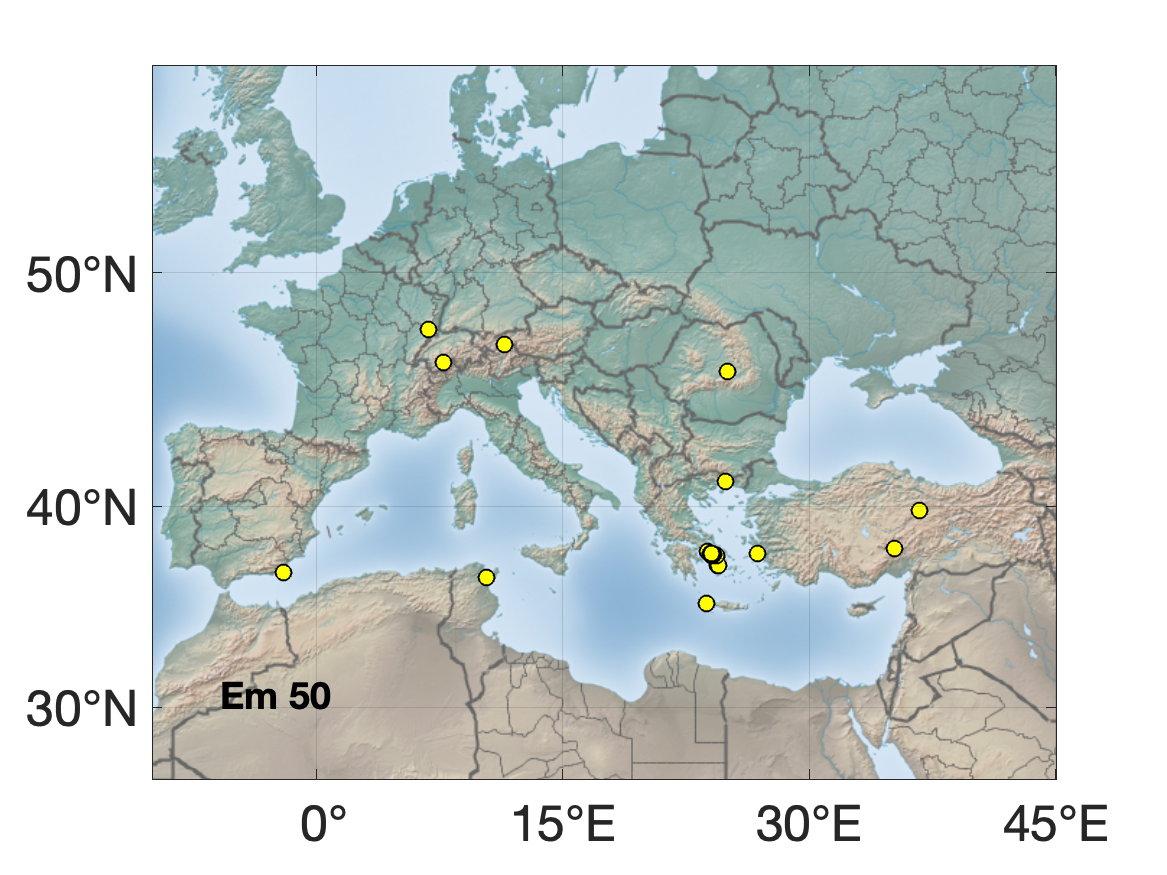

Supplement: Supplementary file 1 — Supplementary file1 (ZIP 46197 KB) [file 12520_2025_2229_MOESM1_ESM.zip › Em 50_map_jittered.png]

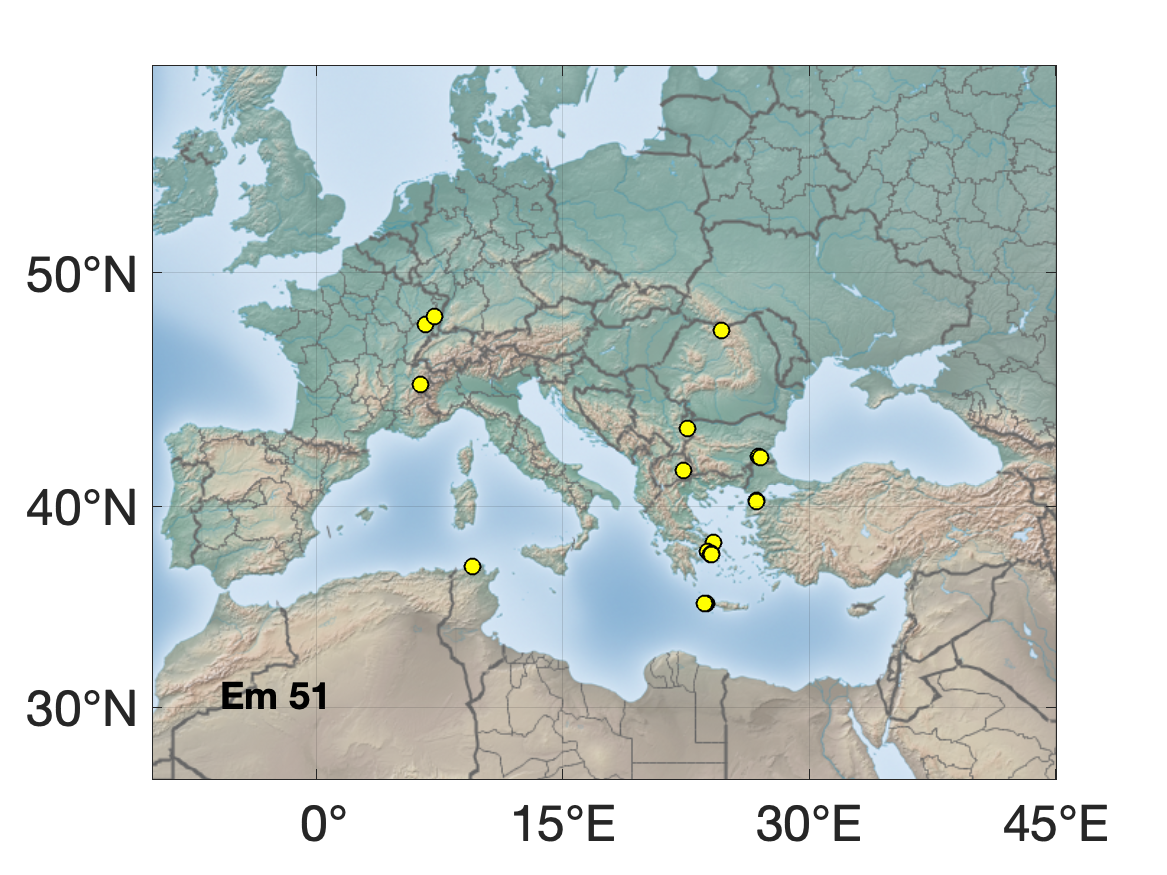

Supplement: Supplementary file 1 — Supplementary file1 (ZIP 46197 KB) [file 12520_2025_2229_MOESM1_ESM.zip › Em 51_map_jittered.png]

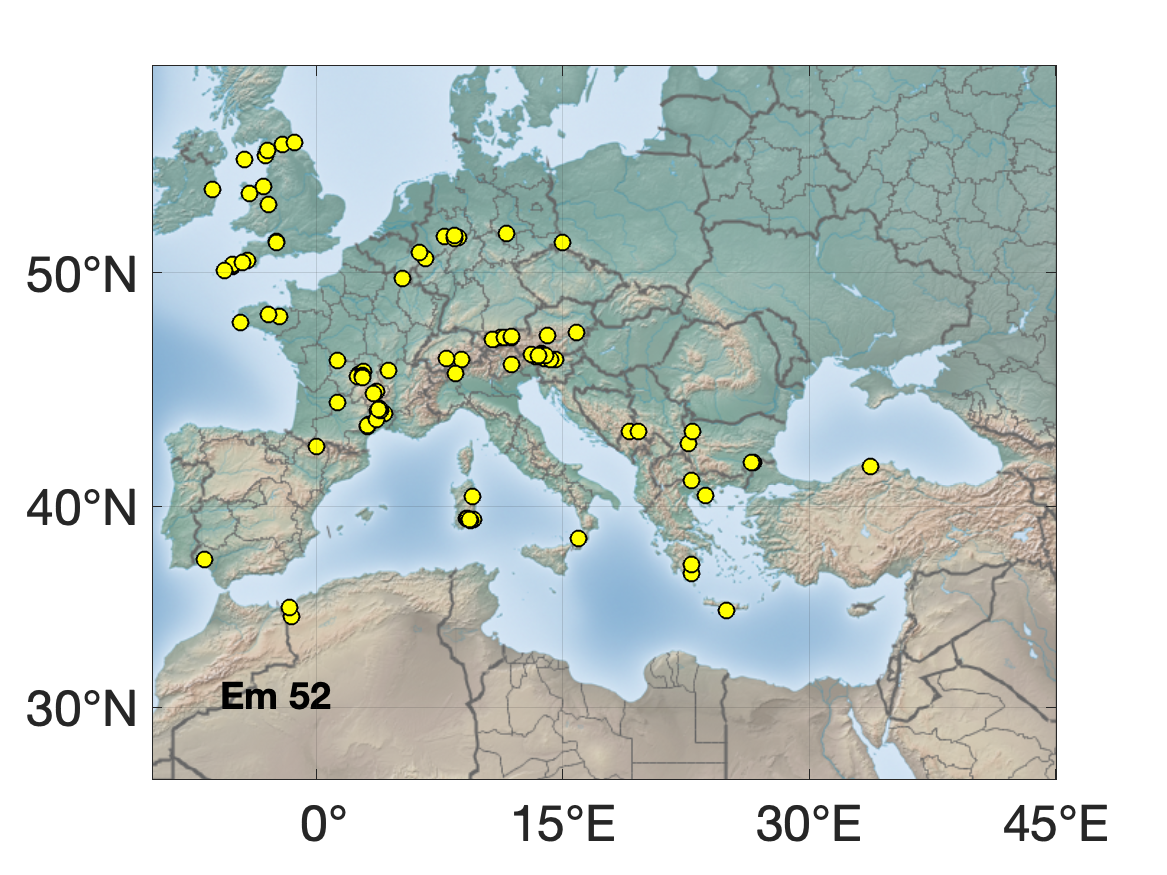

Supplement: Supplementary file 1 — Supplementary file1 (ZIP 46197 KB) [file 12520_2025_2229_MOESM1_ESM.zip › Em 52_map_jittered.png]

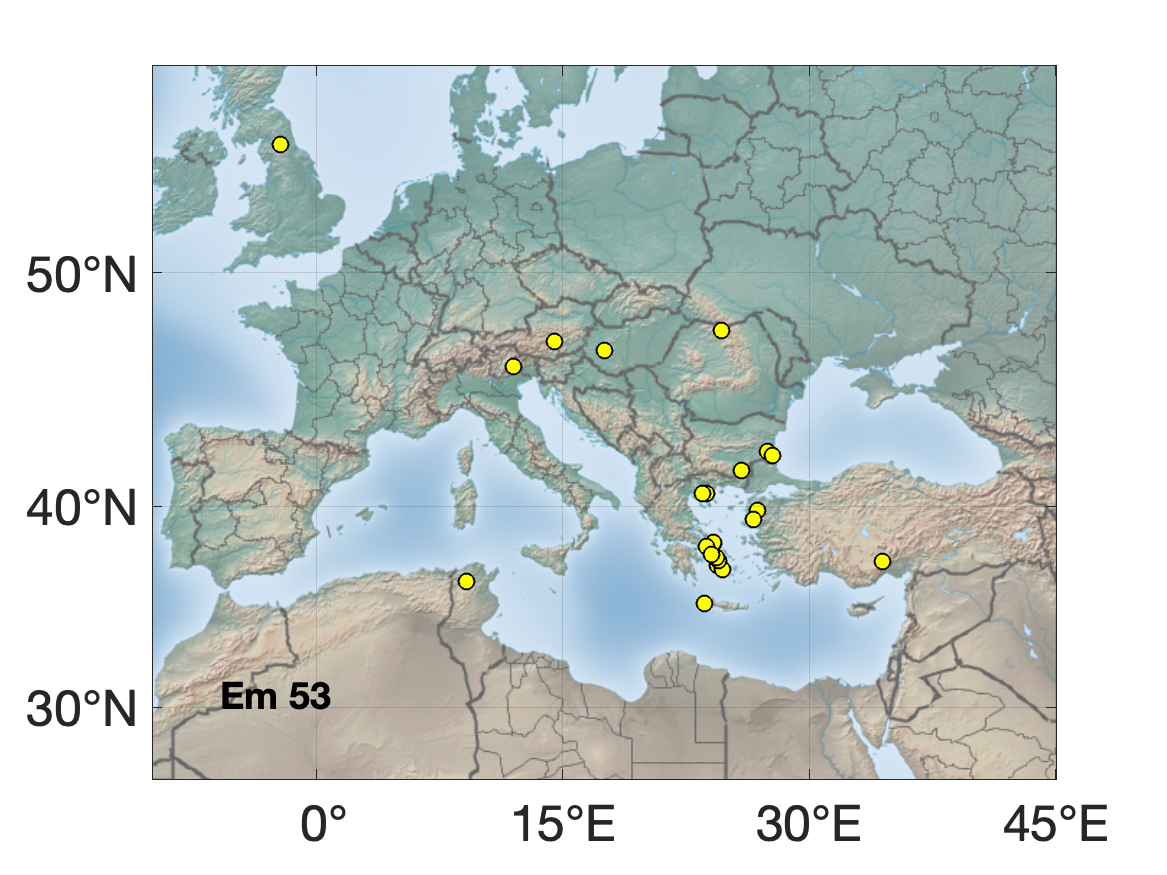

Supplement: Supplementary file 1 — Supplementary file1 (ZIP 46197 KB) [file 12520_2025_2229_MOESM1_ESM.zip › Em 53_map_jittered.png]

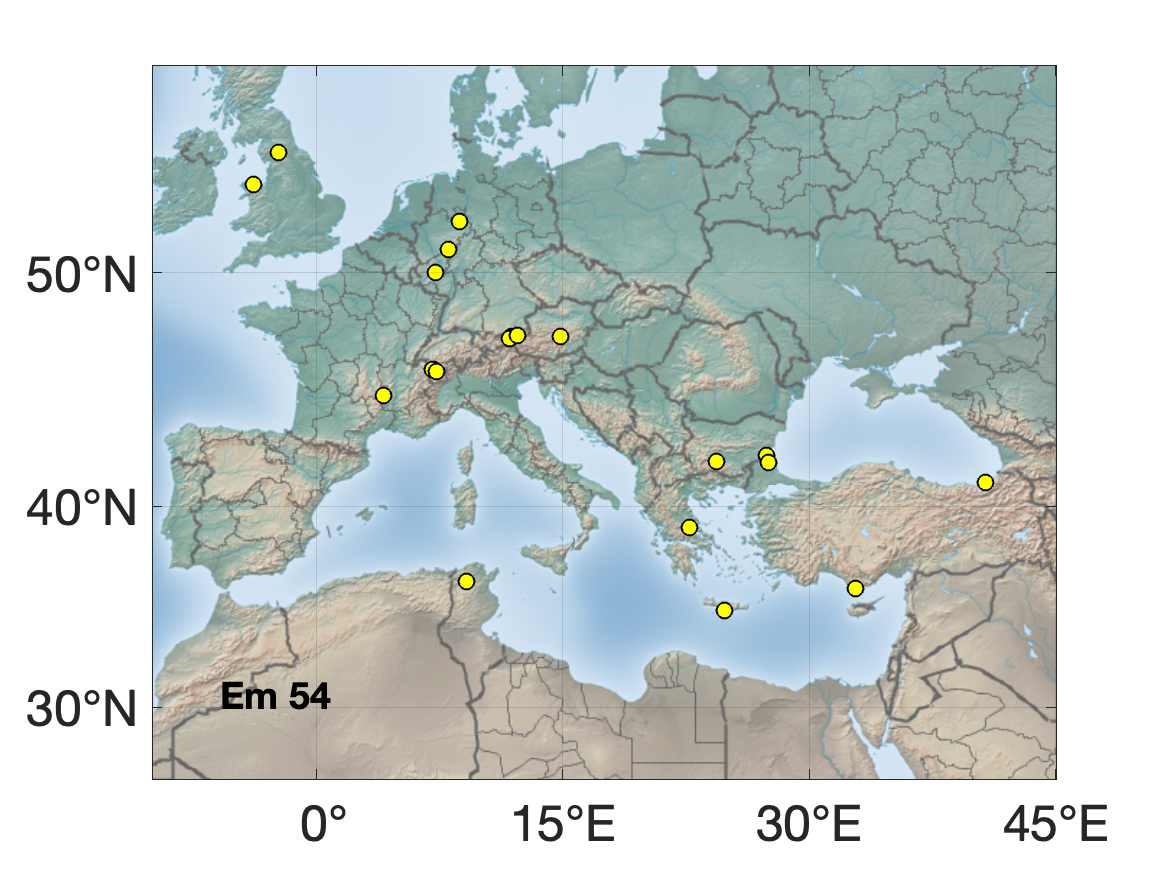

Supplement: Supplementary file 1 — Supplementary file1 (ZIP 46197 KB) [file 12520_2025_2229_MOESM1_ESM.zip › Em 54_map_jittered.png]

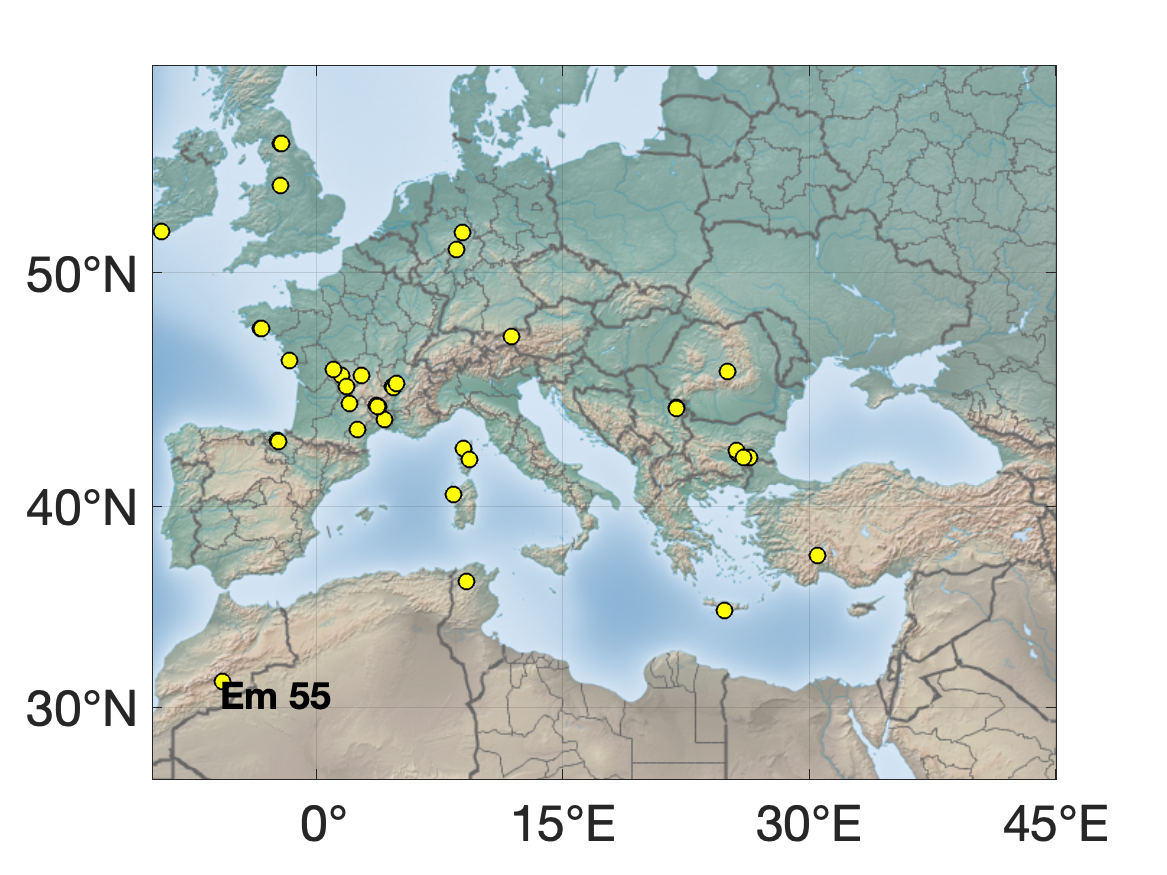

Supplement: Supplementary file 1 — Supplementary file1 (ZIP 46197 KB) [file 12520_2025_2229_MOESM1_ESM.zip › Em 55_map_jittered.png]

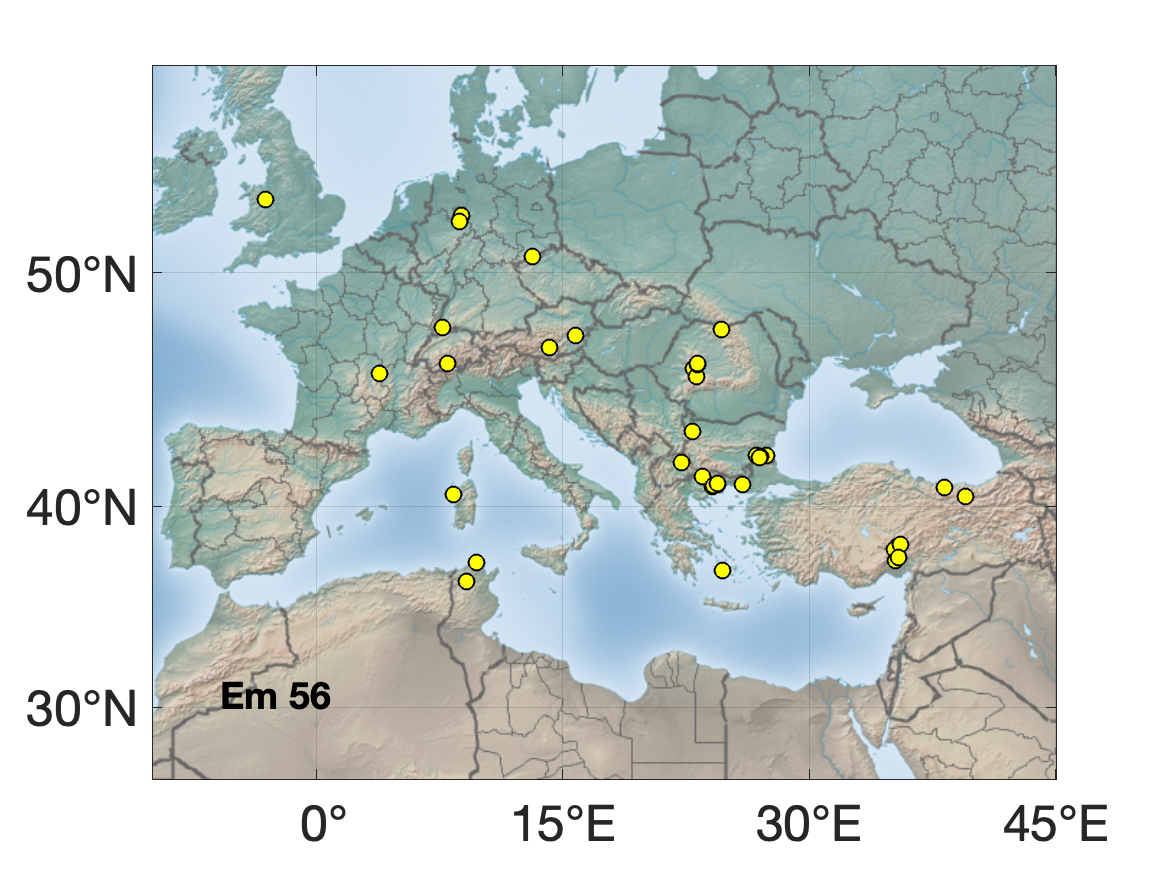

Supplement: Supplementary file 1 — Supplementary file1 (ZIP 46197 KB) [file 12520_2025_2229_MOESM1_ESM.zip › Em 56_map_jittered.png]

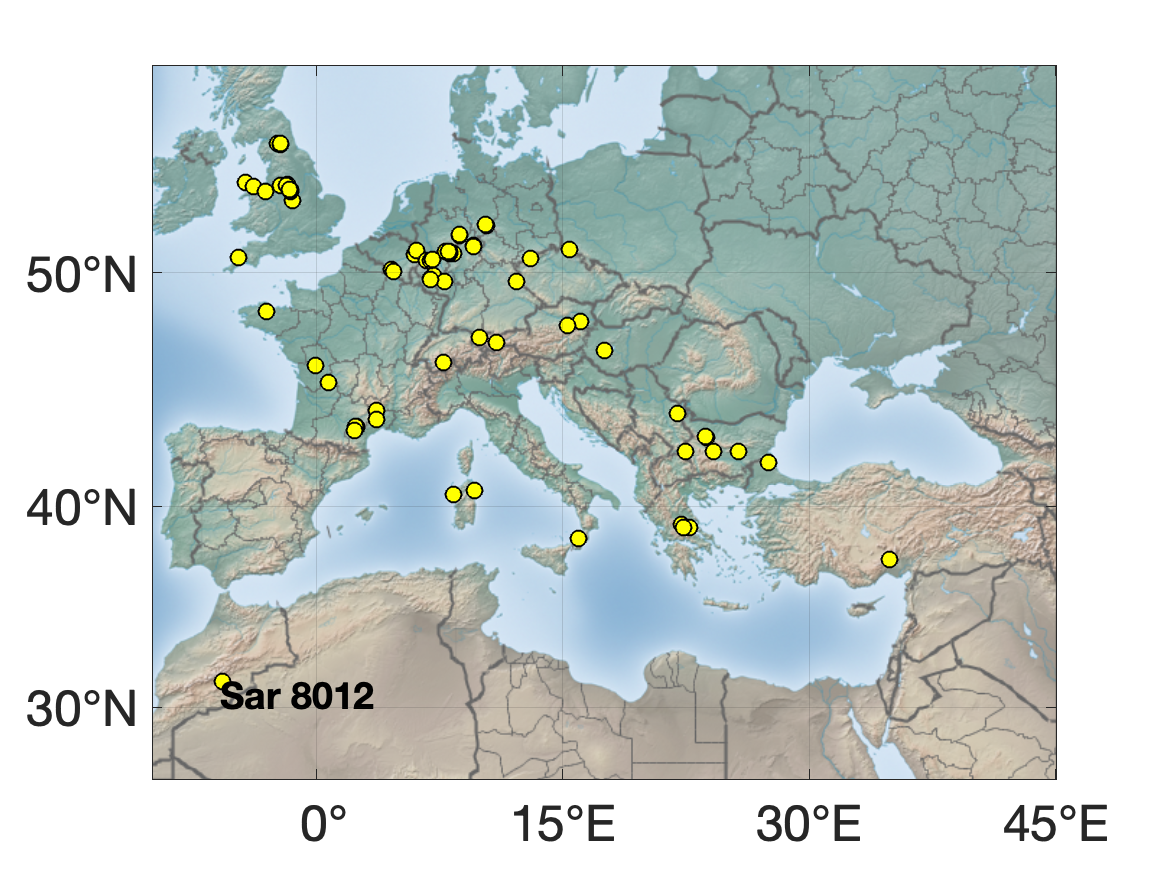

Supplement: Supplementary file 1 — Supplementary file1 (ZIP 46197 KB) [file 12520_2025_2229_MOESM1_ESM.zip › Sar 8012_map_jittered.png]

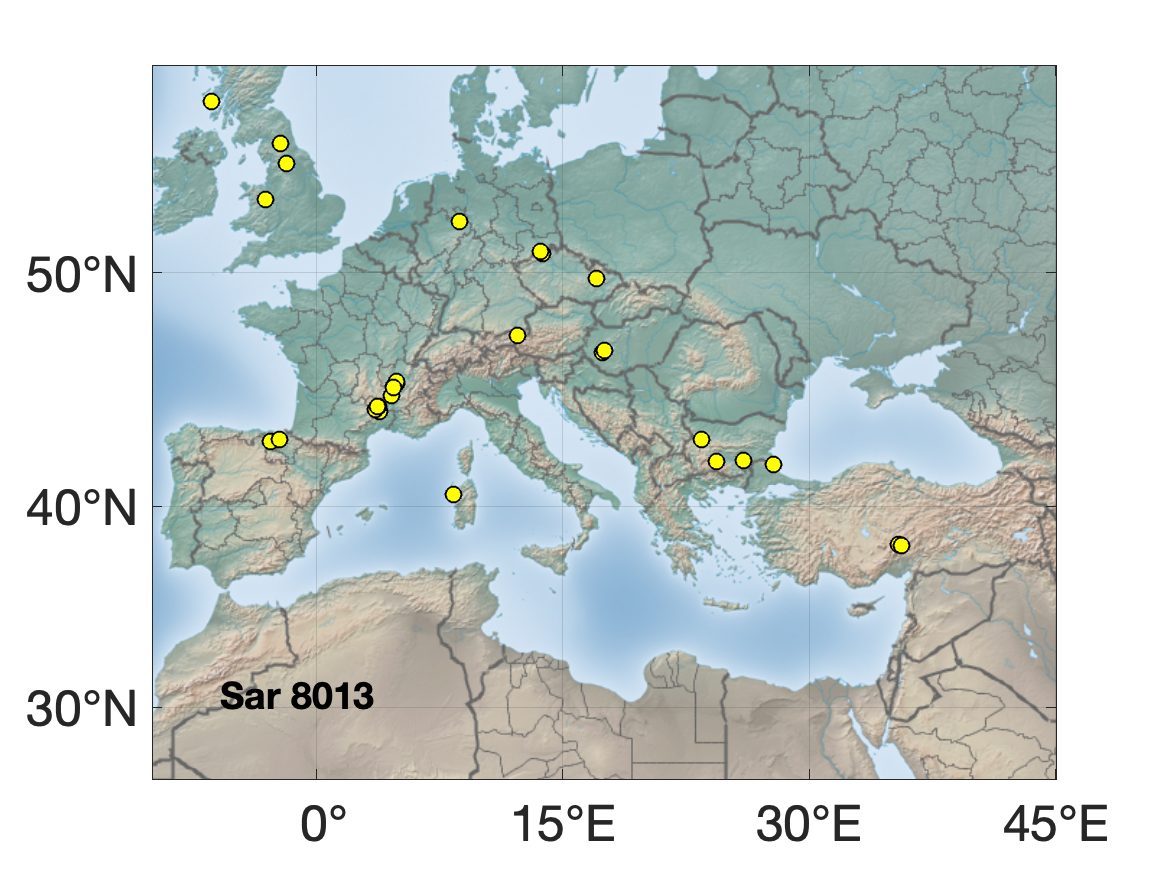

Supplement: Supplementary file 1 — Supplementary file1 (ZIP 46197 KB) [file 12520_2025_2229_MOESM1_ESM.zip › Sar 8013_map_jittered.png]

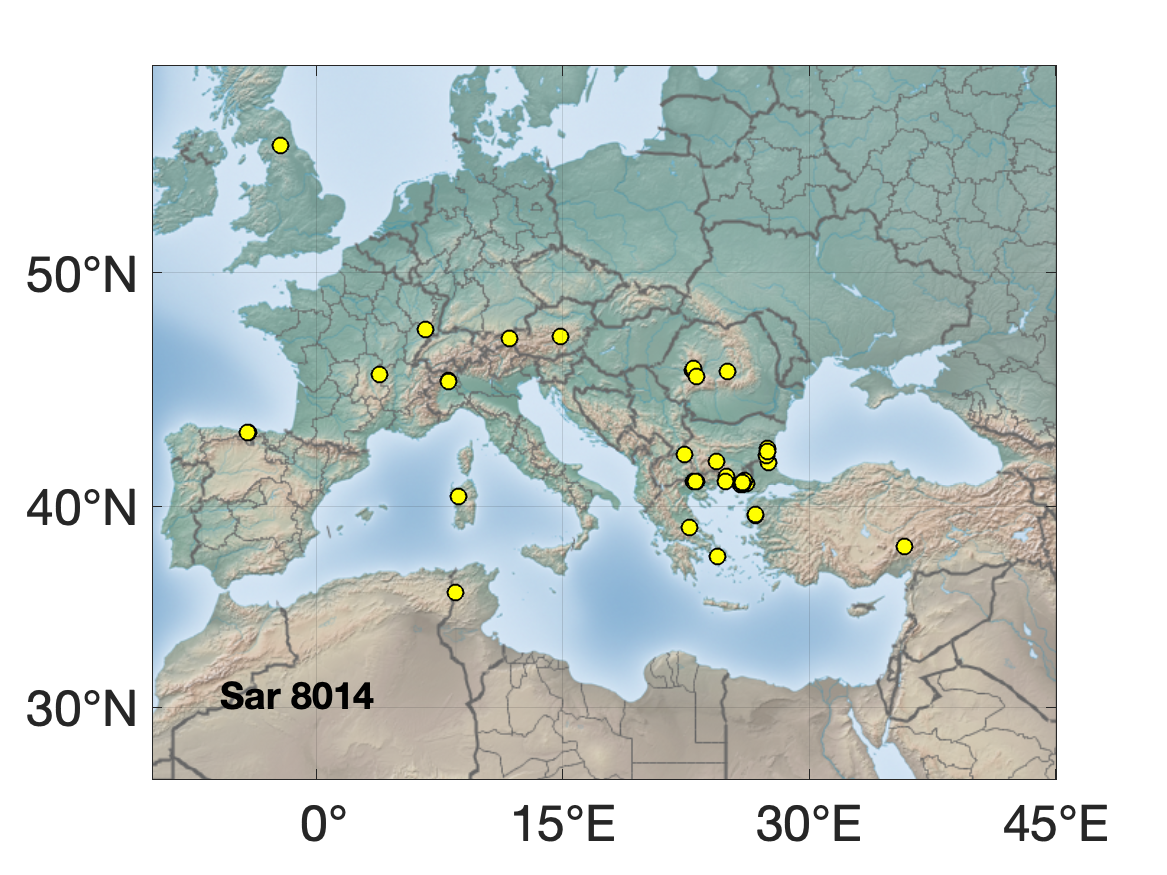

Supplement: Supplementary file 1 — Supplementary file1 (ZIP 46197 KB) [file 12520_2025_2229_MOESM1_ESM.zip › Sar 8014_map_jittered.png]

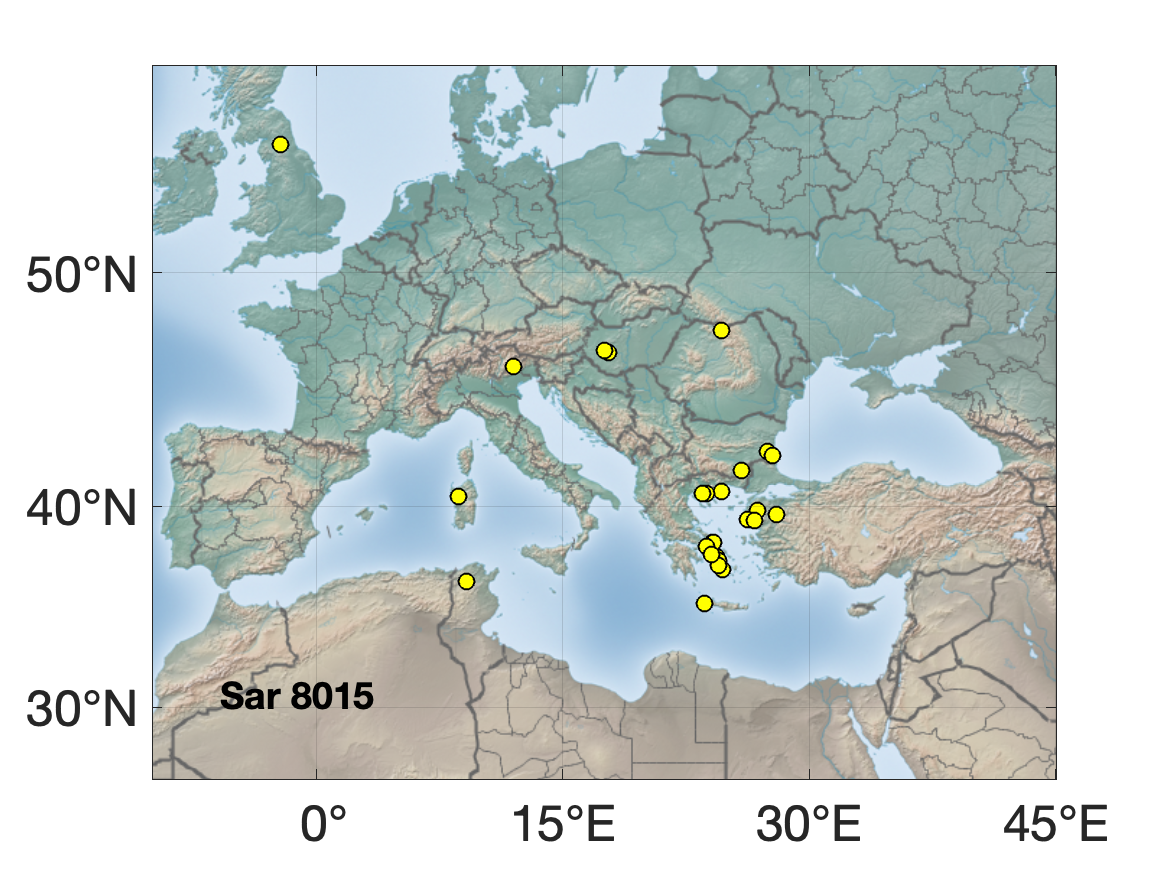

Supplement: Supplementary file 1 — Supplementary file1 (ZIP 46197 KB) [file 12520_2025_2229_MOESM1_ESM.zip › Sar 8015_map_jittered.png]

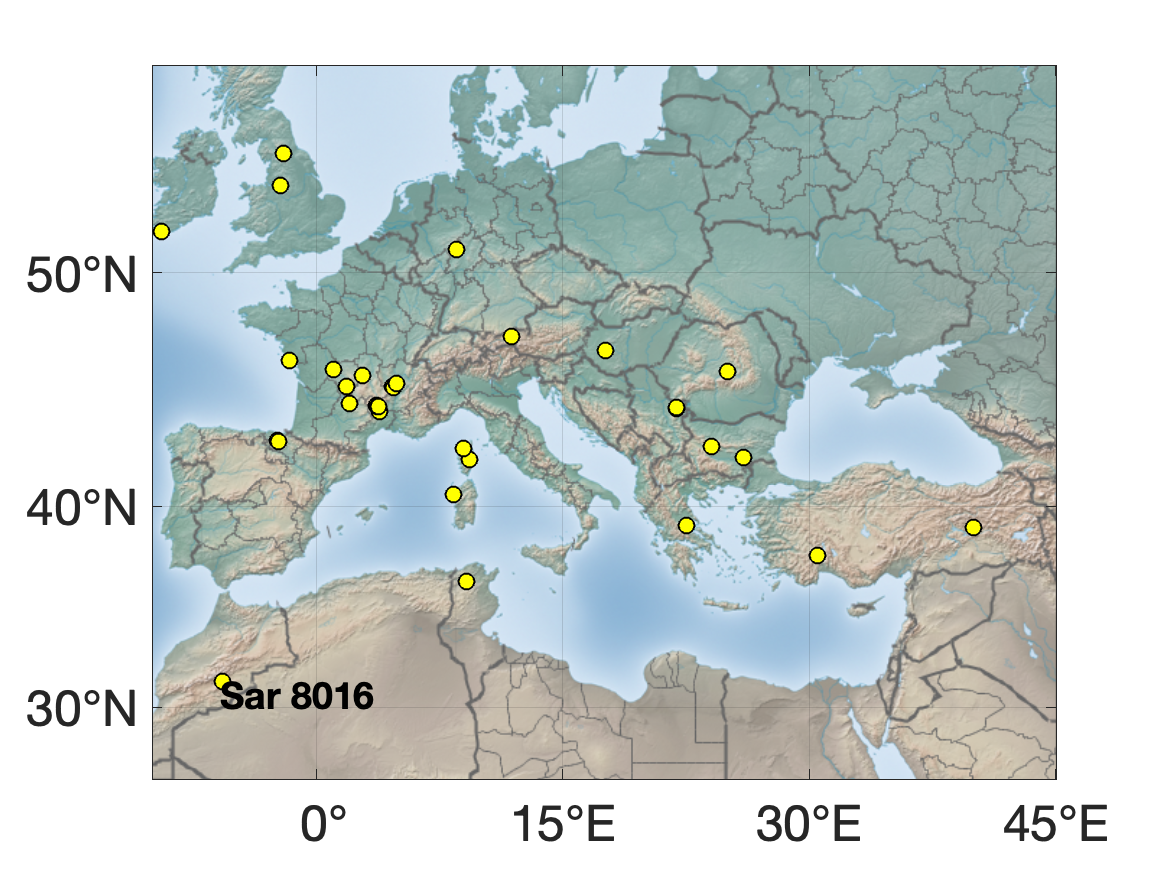

Supplement: Supplementary file 1 — Supplementary file1 (ZIP 46197 KB) [file 12520_2025_2229_MOESM1_ESM.zip › Sar 8016_map_jittered.png]

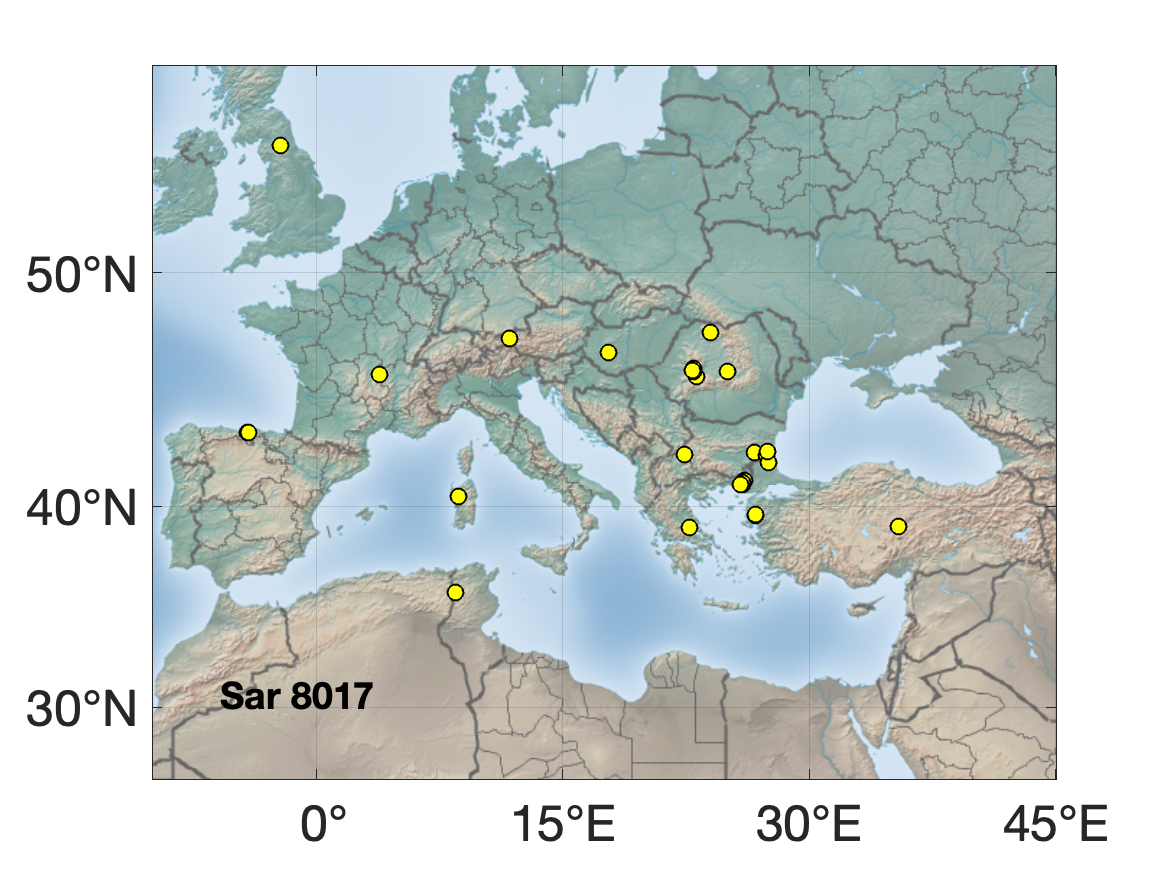

Supplement: Supplementary file 1 — Supplementary file1 (ZIP 46197 KB) [file 12520_2025_2229_MOESM1_ESM.zip › Sar 8017_map_jittered.png]

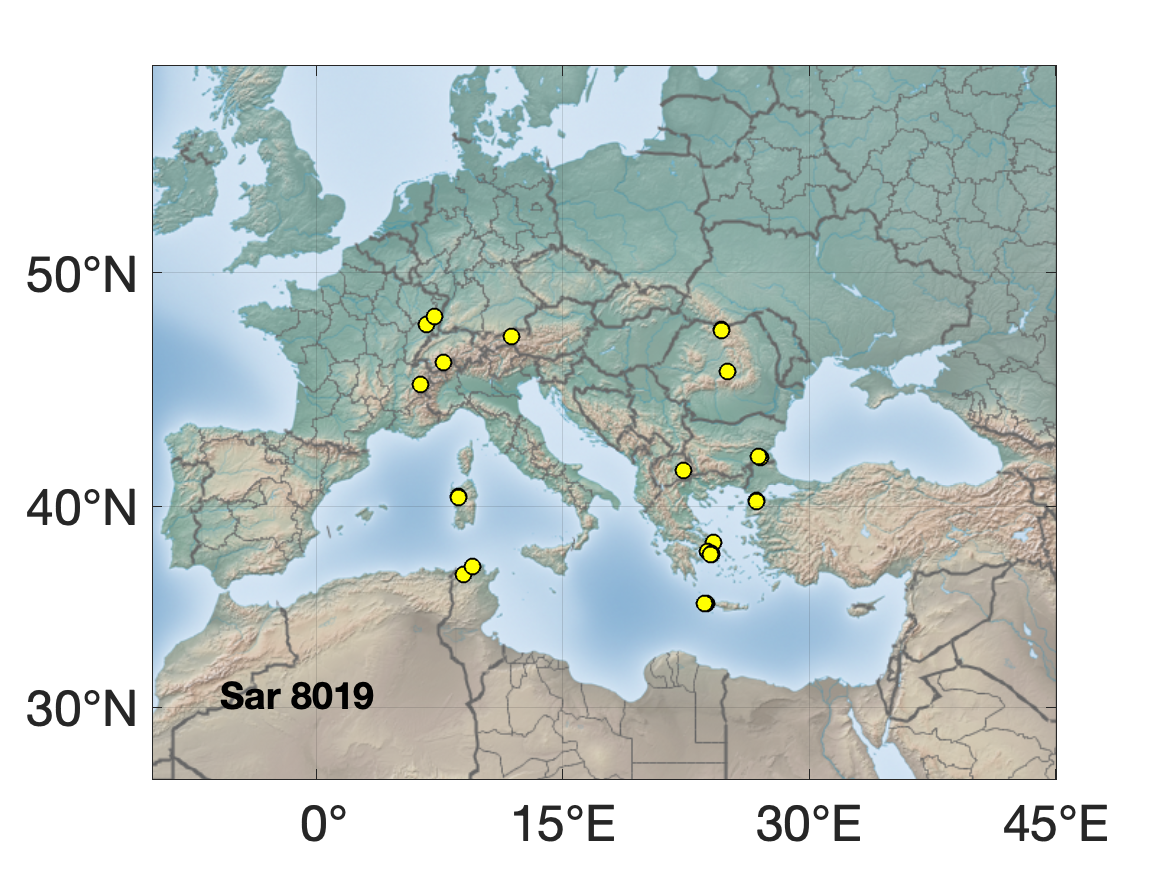

Supplement: Supplementary file 1 — Supplementary file1 (ZIP 46197 KB) [file 12520_2025_2229_MOESM1_ESM.zip › Sar 8019_map_jittered.png]

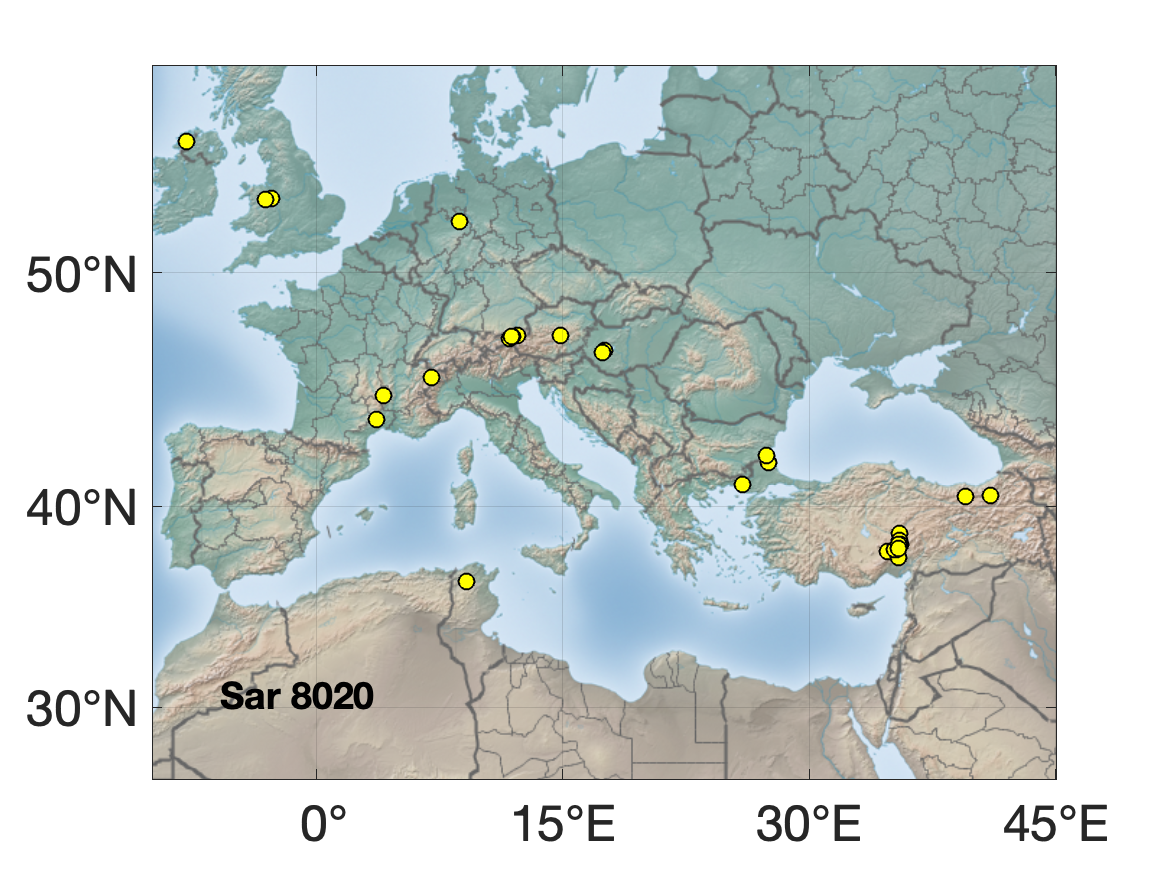

Supplement: Supplementary file 1 — Supplementary file1 (ZIP 46197 KB) [file 12520_2025_2229_MOESM1_ESM.zip › Sar 8020_map_jittered.png]

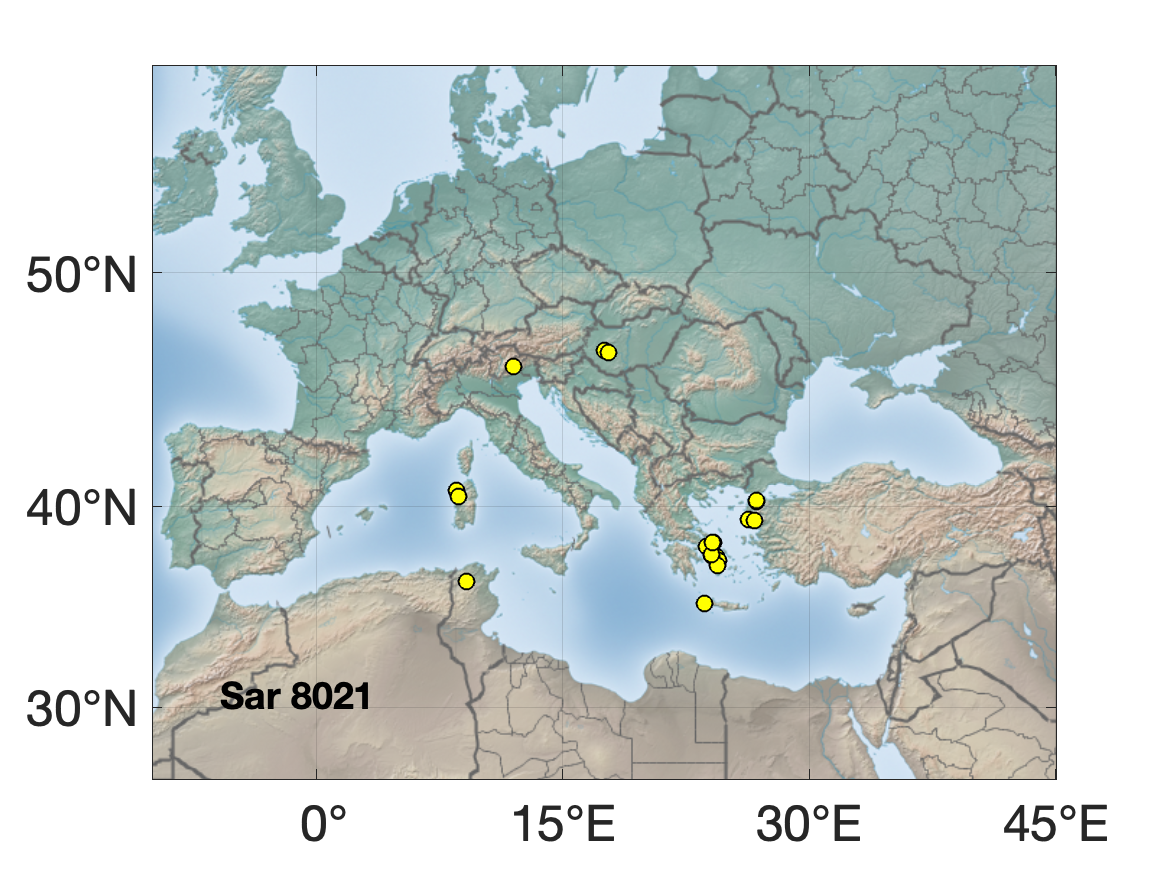

Supplement: Supplementary file 1 — Supplementary file1 (ZIP 46197 KB) [file 12520_2025_2229_MOESM1_ESM.zip › Sar 8021_map_jittered.png]

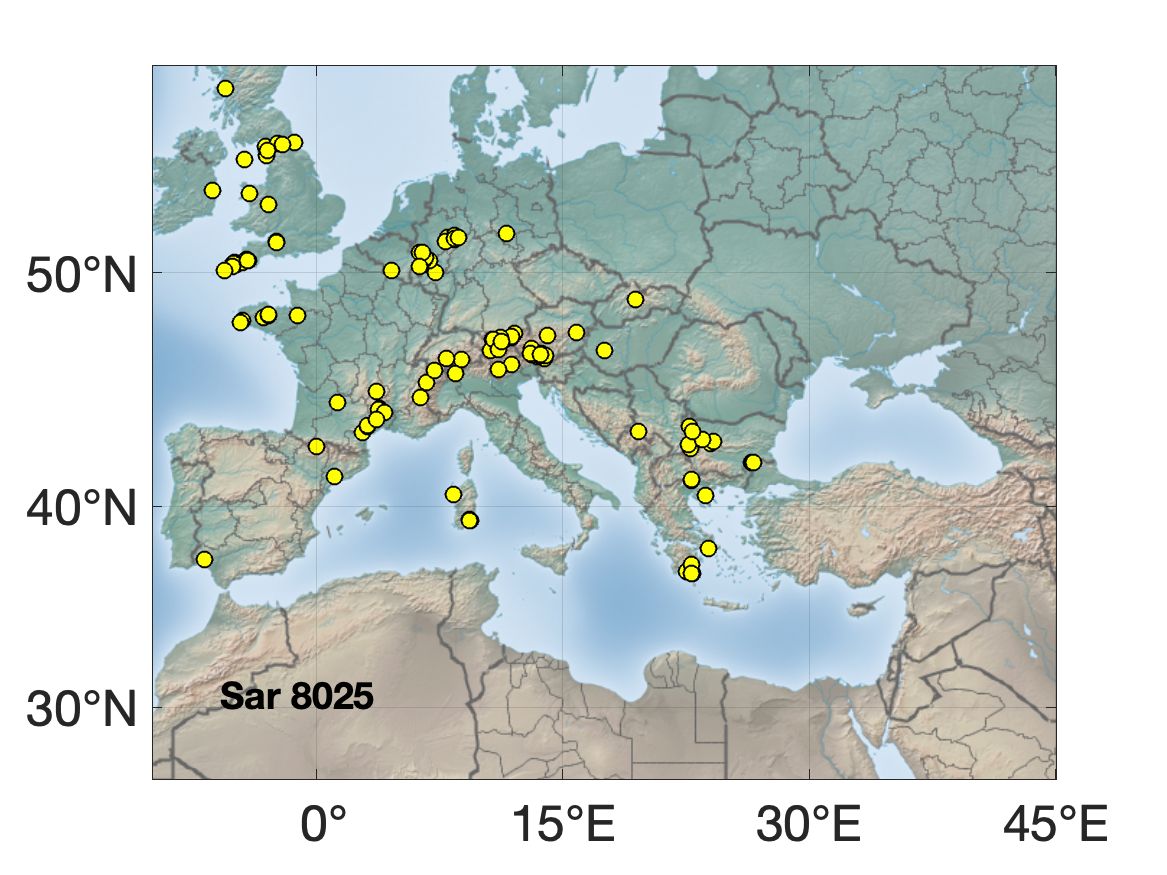

Supplement: Supplementary file 1 — Supplementary file1 (ZIP 46197 KB) [file 12520_2025_2229_MOESM1_ESM.zip › Sar 8025_map_jittered.png]

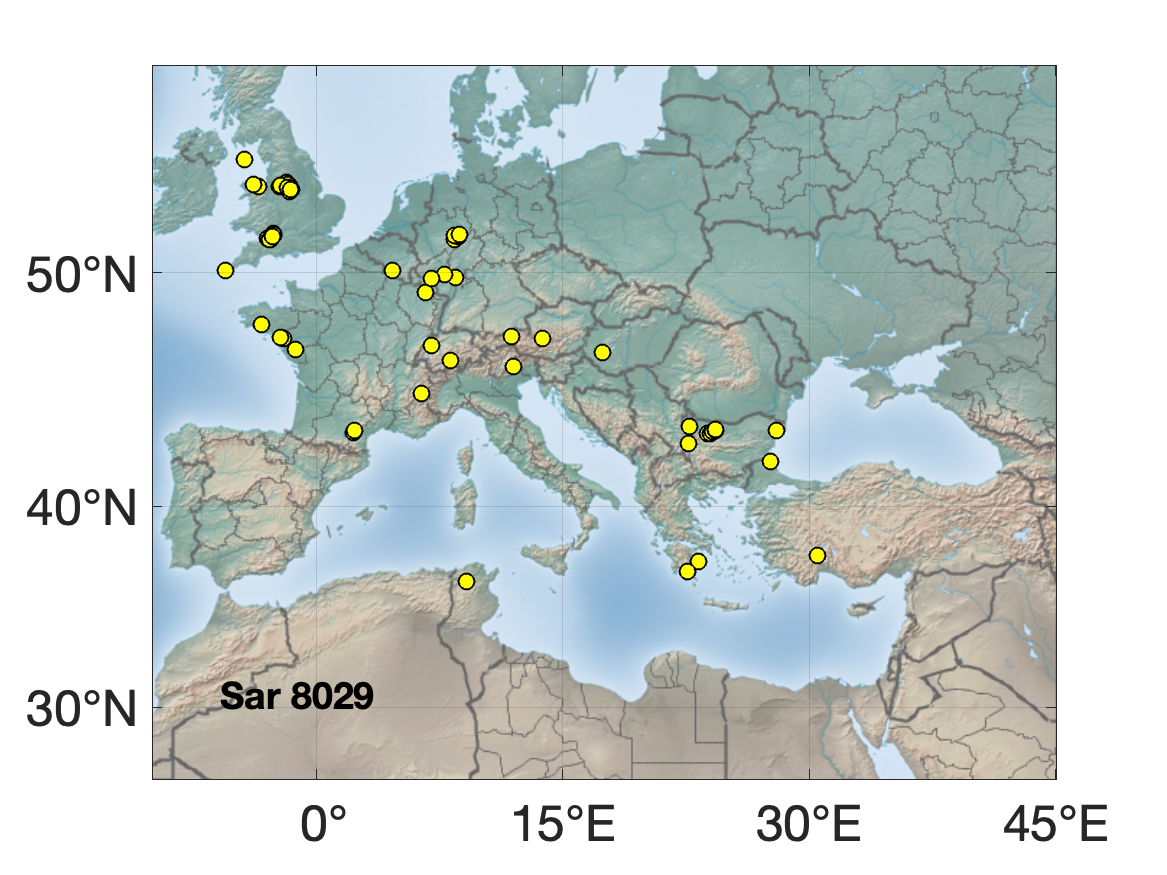

Supplement: Supplementary file 1 — Supplementary file1 (ZIP 46197 KB) [file 12520_2025_2229_MOESM1_ESM.zip › Sar 8029_map_jittered.png]

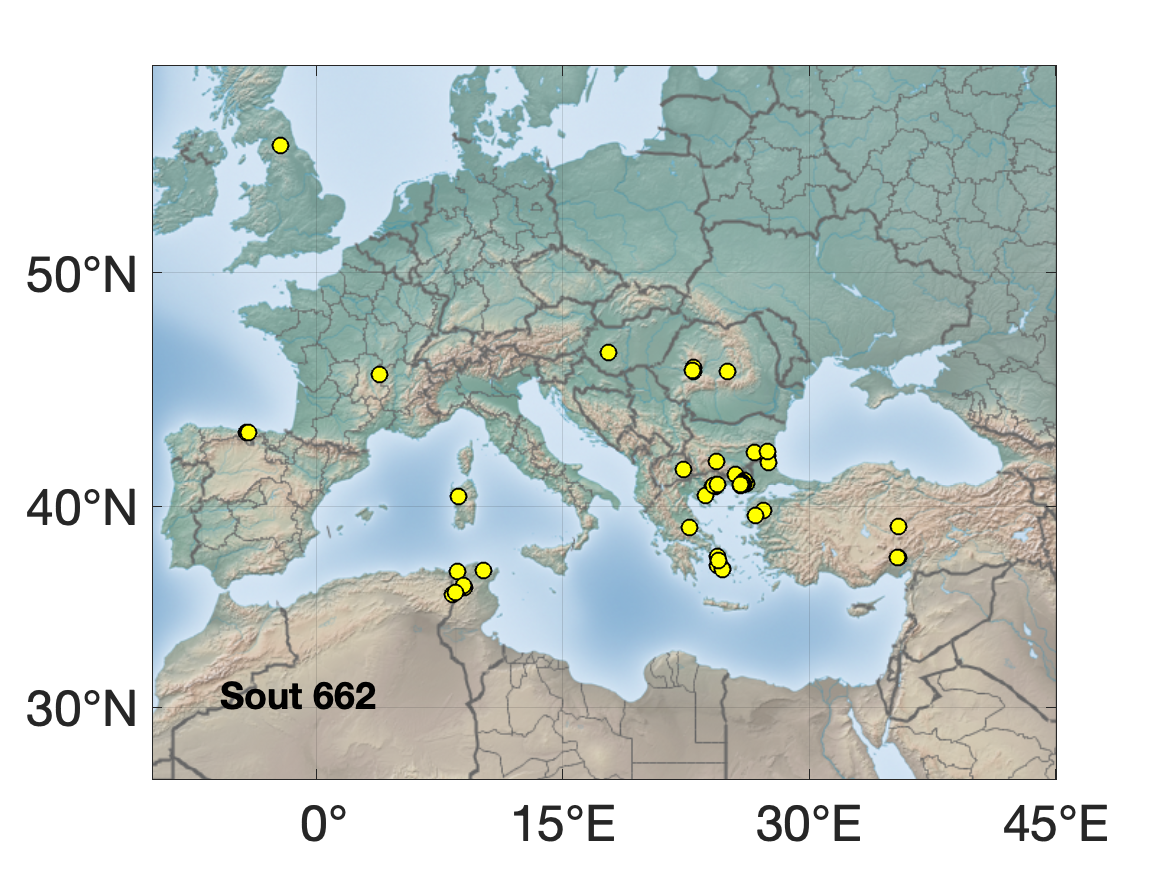

Supplement: Supplementary file 1 — Supplementary file1 (ZIP 46197 KB) [file 12520_2025_2229_MOESM1_ESM.zip › Sout 662_map_jittered.png]

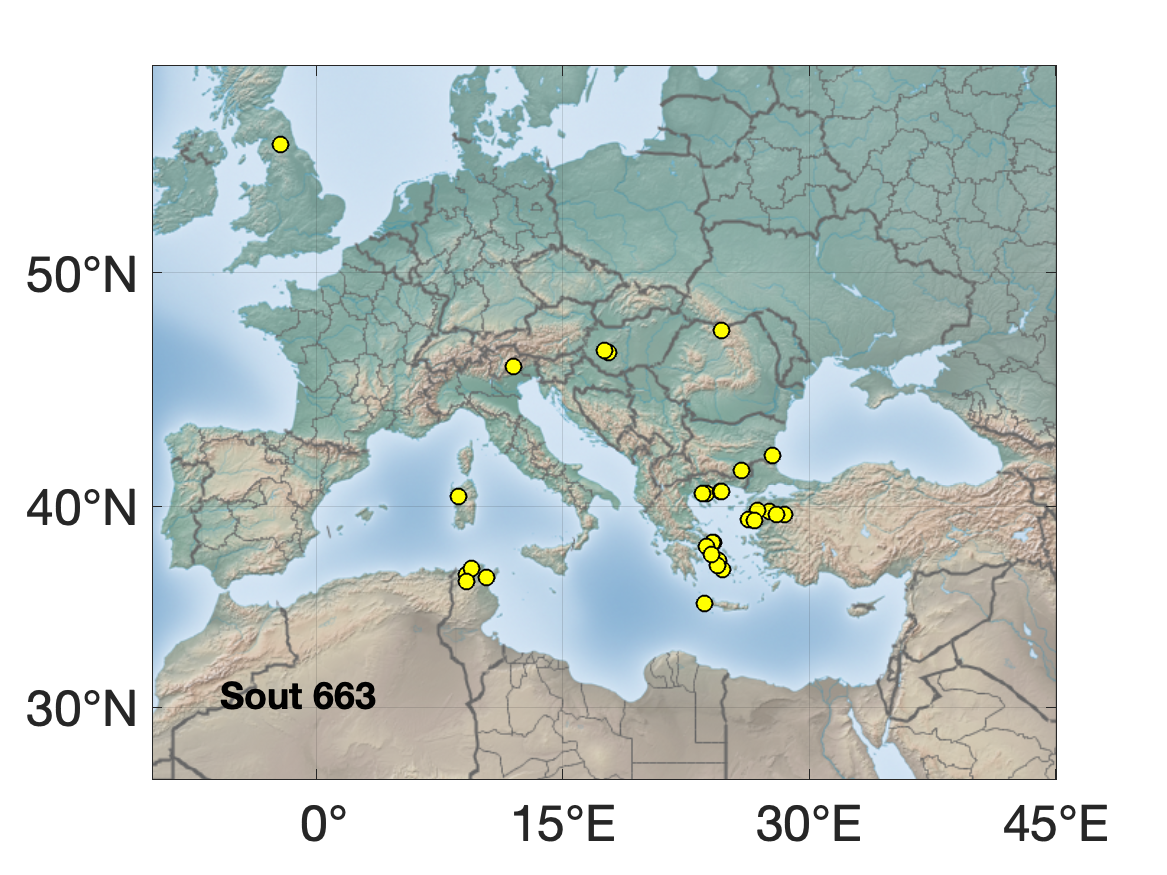

Supplement: Supplementary file 1 — Supplementary file1 (ZIP 46197 KB) [file 12520_2025_2229_MOESM1_ESM.zip › Sout 663_map_jittered.png]
